# Acceptability of a high-protein Mediterranean-style diet and resistance exercise protocol for cardiac rehabilitation patients: Involving service users in intervention design using a mixed-methods participatory approach

**DOI:** 10.3389/fnut.2023.1043391

**Published:** 2023-02-14

**Authors:** Richard Kirwan, Lisa Newson, Deaglan McCullough, Tom Butler, Ian G. Davies, Fatima Perez de Heredia

**Affiliations:** ^1^School of Biological and Environmental Sciences, Liverpool John Moores University, Liverpool, United Kingdom; ^2^Research Institute for Sport and Exercise Sciences, Liverpool John Moores University, Liverpool, United Kingdom; ^3^The Institute for Health Research, Liverpool John Moores University, Liverpool, United Kingdom; ^4^School of Psychology, Liverpool John Moores University, Liverpool, United Kingdom; ^5^Carnegie School of Sport, Leeds Beckett University, Leeds, United Kingdom; ^6^Faculty of Health, Social Care and Medicine, Edge Hill University, Ormskirk, United Kingdom; ^7^Cardiorespiratory Research Centre, Edge Hill University, Ormskirk, United Kingdom

**Keywords:** cardiovascular disease, resistance exercise, Mediterranean diet, high-protein diet, sarcopenia, sarcopenic obesity, cardiometabolic disease, mixed method analysis

## Abstract

**Background:**

Current cardiac rehabilitation (CR) practices focus on aerobic-style exercise with minimal nutrition advice. This approach may not be optimal for CR patients with reduced muscle mass and elevated fat mass. Higher protein, Mediterranean-style diets combined with resistance exercise (RE) may improve muscle mass and reduce the risk of future cardiovascular events, although such an approach is yet to be trialed in a CR population.

**Objective:**

We explored patient perspectives on the proposed design of a feasibility study. Patients reflected on the acceptability of a proposed high-protein Mediterranean-style diet and RE protocol, emphasizing research methodology and the acceptability of the proposed recipes and exercises.

**Design:**

We applied quantitative and qualitative (mixed methods) approaches. The quantitative approach involved an online questionnaire (*n* = 40) regarding the proposed study methodology and relevance. A subset of participants (*n* = 12) received proposed recipe guides and were asked to prepare several dishes and complete an online questionnaire regarding their experience. Another subset (*n* = 18) received links to videos of the proposed RE and completed a questionnaire regarding their impressions of them. Finally, semi-structured interviews (*n* = 7) were carried out to explore participants’ impressions of the proposed diet and exercise intervention.

**Results:**

Quantitative data indicated a high level of understanding of the intervention protocol and its importance within the context of this research. There was a high degree of willingness to participate in all aspects of the proposed study (>90%). The trialed recipes were enjoyed and found to be easy to make by a majority of participants (79 and 92.1%, respectively). For the proposed exercises 96.5% of responses agreed they would be willing to perform them and, 75.8% of responses agreed they would enjoy them. Qualitative analysis revealed that participants viewed the research proposal, diet, and exercise protocol in a positive light. The research materials were considered appropriate and well explained. Participants suggested practical recommendations for improving recipe guides and requested more individual-focused exercise recommendations, and more information on the specific health benefits of the diet and exercise protocols.

**Conclusion:**

The study methodology and the specific dietary intervention and exercise protocol were found to be generally acceptable with some suggested refinements.

## 1. Introduction

Cardiovascular disease (CVD) is the leading cause of death worldwide with almost 19.1 million deaths reported in 2020 (1). Individuals who experience or are at high risk of, a cardiac event can be referred to cardiac rehabilitation (CR) (2), a lifestyle intervention program predominantly focusing on aerobic exercise, and may involve other components such as advice on diet quality and weight management, smoking cessation, and stress reduction (3). The objective of CR is to reduce the risk of future cardiac events and improve quality of life, and considerable evidence points to its efficacy (4–6).

Sarcopenic obesity (SO), the combination of reduced muscle mass and function associated with aging (sarcopenia) and excessively elevated body fat (obesity) (7), has been observed to contribute to a greater risk of CVD (8, 9). Indeed, SO may at least partially explain the phenomenon known as the obesity paradox, whereby lower body mass index (BMI) in cardiac populations is associated with increased mortality (10–12). The increased risk of CVD in those with SO may result from higher levels of pro-inflammatory cytokines produced in visceral adipose tissue (VAT), known to be elevated in individuals with SO (13). These cytokines can contribute to detrimental changes in cardiometabolic (CM) risk factors such as insulin resistance and dyslipidaemia (14–16). These pro-inflammatory molecules may further contribute to the progression of SO through their association with reduced muscle mass and strength (17). Reduced muscle mass and function may also contribute to reductions in physical activity levels in adults (18), which can reduce cardiorespiratory fitness and lead to an increased risk of CVD (19, 20).

Accordingly, increasing muscle mass may be an appropriate target in CR patients presenting with SO. Resistance exercise (RE) and increased protein intake are widely used strategies for increasing muscle mass and strength in older adults (21). However, while RE may be incorporated into some CR programmes, there is a clear emphasis on aerobic exercise-based CR (22–25). Similarly, while Mediterranean diets are recommended to reduce CVD risk (26–32), there is little evidence for adherence to such dietary advice in current CR practices, particularly in non-Mediterranean populations (33, 34). A randomized controlled trial (RCT) trialing a high-protein, Mediterranean-style diet combined with RE in a CR population would contribute evidence for the efficacy of such an approach.

Patient and public involvement (PPI) in the early stages of developing a feasibility study for such an intervention is recognized as good practice and can greatly contribute to the acceptability of such an intervention (35). Both quantitative and qualitative methods can be employed to identify potential barriers to change, determine understanding of the relevance of specific interventions, and help to refine the proposed methodology, thereby potentially improving engagement and adherence prior to the implementation of an RCT (35, 36).

The current study was designed to assess and refine the proposed methodology for a high-protein Mediterranean-style diet and Resistance Exercise in cardiac Rehabilitation (PRiMER) (37). Therefore, to develop a more comprehensive understanding of CVD patient impressions of the proposed intervention particularly on proposed exercises and recipe acceptability, we conducted a mixed methods study involving both qualitative and quantitative research methods (38).

## 2. Materials and methods

### 2.1. Study design

The study consisted of (i) a cross-sectional, online questionnaire and (ii) a phone interview, conducted amongst individuals with a diagnosis, history or elevated risk of CVD, with questions focused on a proposed high-protein Mediterranean-style diet and RE intervention for CR patients (37). This methodology was used due to COVID-19-related social distancing restrictions implemented in the UK at the time of data collection.

The principles of a “person-based” approach were used to help refine and evaluate the proposed intervention. Such a person-based approach may help those designing the intervention to better understand how potential participants, as individuals, react to the proposed methodology and identify which aspects may need to be refined for a more feasible implementation (36). Core-elements of such an approach include (i) intervention planning, (ii) design, and (iii) evaluation of acceptability (36). The initial planning and design of the proposed intervention were carried out in 2019 with the assistance of the Liverpool Heart and Chest Hospital (LHCH) Service Users Research Endeavour (SURE) group^1^, a PPI group, and CR staff from LHCH Knowsley Community Cardiovascular Service (KCCS).

### 2.2. Recruitment

**FIGURE 1:**
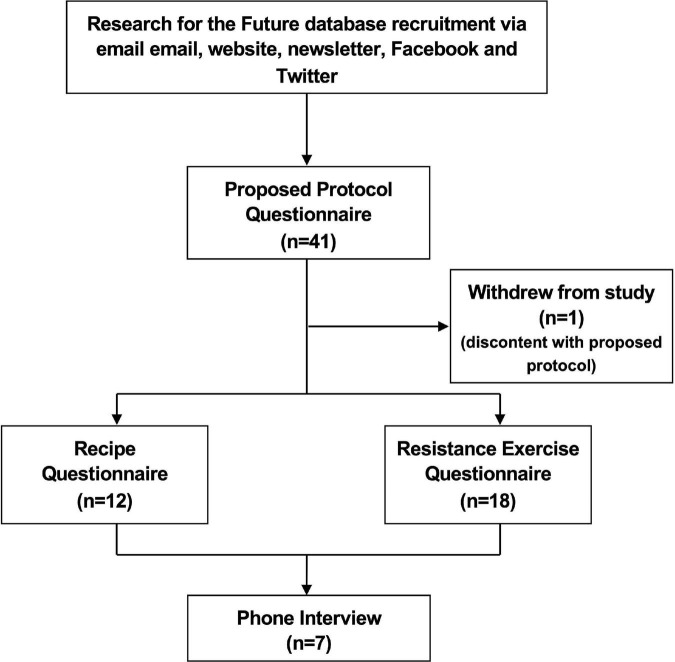
Study flow diagram.

Individuals registered as having a diagnosis or history of CVD or type 2 diabetes (T2D) in the Research for the Future (RftF) database were presented with a research survey link *via* a combination of email and announcements on the RftF website, newsletter, Facebook, and Twitter accounts in June and July of 2020. Research for the Future^2^ is an initiative of the National Institute for Health and Care Research Clinical Research Network (NIHR CRN)^3^ to facilitate recruitment to NIHR and other health research studies. Individuals with T2D were included due to the elevated risk of CVD in this population (39). The link included a participant information sheet with information on the study as well as a consent form. Inclusion criteria were: (i) a diagnosis of a cardiovascular condition or diagnosis as high risk for a cardiac condition, and (ii) previous referral to CR. As the inclusion criteria were considered sufficient for a PPI study, no exclusion criteria were used. A study flow diagram is presented in Figure 1.

### 2.3. Ethical approval

Ethical approval for the intervention study was granted by the National Health Service North West–Greater Manchester East Research Ethics Committee (IRAS ID: 256927, REC reference: 19/NW/0762). The study was conducted according to the ethical principles of the Declaration of Helsinki (40), and online informed consent was obtained from all participants before participation.

### 2.4. Online and telephone questionnaires

The questionnaires were administered through JISC Online Surveys (Bristol, UK^4^) and took approximately 30 min to complete. For the initial online questionnaire, after completing questions on demographics, participants were asked to read the proposed research plan (Supplementary Figure 1) and were told that all the following questions would relate to this plan. Briefly, the research plan included information on the relevance of the research in relation to heart disease, muscle mass, and CR. The plan also included a brief description of the proposed research intervention including:

• Inclusion/exclusion criteria
• Proposed intervention arms
• Proposed dietary requirements, including information on the characteristics and benefits of a Mediterranean-style diet
• Proposed exercise modality
• A study research plan (Supplementary Figure 1)

Participants then completed questions related to their understanding and thoughts regarding the proposed protocol, as well as questions regarding their previous knowledge about the Mediterranean diet and its relation to health. The two final questions asked participants if they would be willing to participate in further evaluation. The first asked participants to refer to a digital recipe book to try the healthy recipes from the study and to reflect on their use, acceptability, and feasibility. Those who agreed were provided with a PDF containing recipes for the proposed intervention (Supplementary File 1). Participants were asked to try as many recipes as they liked and reply to an online questionnaire regarding the preparation of the recipes and their impression of the finished meals. These participants were also invited to engage in a semi-structured interview concerning their thoughts, opinions and recommendations regarding the proposed intervention and recipes.

The final question asked participants to complete a follow-up questionnaire related to the proposed RE protocol (Supplementary File 2). This questionnaire explored how the proposed exercise protocol would be carried out and linked to videos of the RE to be included in the proposed intervention. Participants were asked to answer questions related to the protocol, as well as their willingness to perform, and impressions of, each of the exercises.

### 2.5. Quantitative analysis

Descriptive statistics were analyzed in R [Version 1.4.1717, R Core Team, (41)]. All quantitative data are presented as frequency of responses and percentage of total response with no further statistical analysis.

### 2.6. Qualitative analysis

For the convenience of the participants, interviews were conducted by telephone. Interviews were digitally audio recorded and later transcribed *verbatim* by the interviewer and first author (RK). Reflective notes were made post-interview, and interview data and analytical notes were discussed between analytical authors (RK, LN) during the analytical process. The interview transcripts were analyzed based on the interpretive-descriptive method (42) to enable the development of themes. The analysis was approached by asking, “what is important to the participants here?” and “what are we learning about the participants’ experiences?” The main themes of the collected data were developed *via* a constant comparative method of data analysis (43).

We acknowledge that using interpretative-descriptive method, the analysis and subsequent themes were influenced by the research team’s subjective interpretations of the data. However, throughout the analytical process, researcher reflexivity and audited discussions (44) occurred aiding researcher triangulation (45) which ensured rigor in the quality of qualitative analysis conducted (46). The qualitative data collection and analysis was conducted by the lead author a white male, early career researcher with specific interests in nutrition in cardiac rehabilitation (RK). The qualitative analysis was led by a female Reader in Applied Health Psychology and a Registered Health Psychologist with expertise in qualitative methodology and long-term conditions (LN). The final version of the qualitative analysis was discussed further with the research team: a white female Senior Lecturer in Physiology with expertise in adipose tissue physiology (FP), a white male Reader in Nutrition with expertise in nutrition and lipidology (ID), a white male Senior Lecturer in Nutrition and Dietetics and a Registered Dietitian with expertise in cardiac rehabilitation (TB), and a white male, early career researcher with expertise in nutrition and exercise physiology (DM).

Direct quotes from a range of participants, which we felt would be transparent in context (46), acted as evidence to support commentary. The authors confirm that the raw data examples supporting this study’s findings are available within the article (see Tables 3–5). Due to the nature of this qualitative research, in line with legal and ethical processes, participants of this study did not agree for their full transcripts to be shared publicly, so supporting data beyond the sample quotation extracts is not feasible. Post-quotes, P1–7 indicates from which participant the *verbatim* quote has been selected. In addition, written feedback from the open-response selection of the questionnaires has also been incorporated into the analysis, to support the validation of findings for each theme; these quotes display (open response) afterwards.

## 3. Results

### 3.1. Demographics

A total of 41 people with a history of CVD, T2D or both, participated in the quantitative questionnaire. One participant asked to be withdrawn from the study citing discontent with the proposed protocol. Demographic details of the sample are presented in Table 1. Fifty percent of participants were female, and 90% were of White British ethnicity; the majority of participants (72%) presented with overweight or obesity, over half of the participants (58%) reported high blood pressure, and 43% reported high cholesterol.

**TABLE 1:** Demographic characteristics of all participants in initial protocol online questionnaire (*n* = 40).

Characteristic	All samples (*n* = 40)	Male (*n* = 20)	Females (*n* = 20)
Age (years)	64.7 ± 13.5	65.6 ± 11.4	63.9 ± 15.6
Age range (years)	21–85	32–85	21–84
Body mass index (kg/m^2^)	30.0 ± 6.7	30.5 ± 6.0	29.5 ± 7.5
*n* Normal (18.5–24.9)	11 (27.5%)	4 (10.0%)	7 (17.5%)
*n* Overweight (25–29.9)	13 (32.5%)	7 (17.5%)	6 (15.0%)
*n* Obese (30<)	16 (40.0%)	9 (22.5%)	7 (17.5%)
White ethnicity (%)	36 (90%)	17 (42.5%)	19 (47.5%)
Do you have high blood pressure? (yes%)	23 (58%)	12 (30.0%)	11 (27.5%)
Do you have high cholesterol? (yes%)	17 (43%)	8 (20.0%)	9 (22.5%)

Data presented as mean ± standard deviation or number (percentage of total population).

### 3.2. Quantitative results

#### 3.2.1. Proposed protocol

##### 3.2.1.1. Importance and willingness to participate in the proposed intervention

**FIGURE 2:**
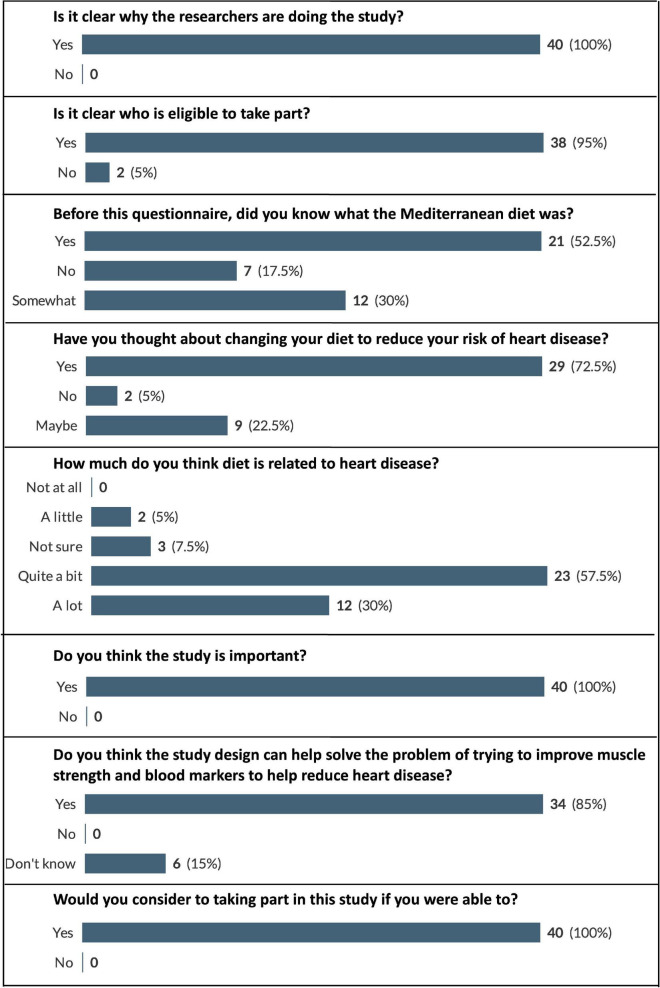
Participant responses to questions relating to intervention importance and willingness to participate. Displayed as number and (percentage) of respondents who selected each answer option (e.g., 100% would represent that all this question’s respondents chose that option).

Data related to participants’ views on the importance of the intervention, the understanding of its relevance and their willingness to participate in such an intervention is displayed in Figure 2. All participants stated that the objectives of the study were clear, that they thought that the intervention was important and that they would be willing to participate in it. Furthermore, 85% of participants replied that they thought the intervention design could to improve muscle strength and blood markers to help reduce heart disease risk.

##### 3.2.1.2. Protocol requirements

Data related to participants’ willingness to undertake the interventions dietary and exercise requirements, and their willingness to undertake the required laboratory procedures is displayed in Table 2. The majority of participants (90% and above) were willing to participate in all aspects of the proposed intervention.

**TABLE 2:** Participant responses to questions relating to intervention protocol requirements.

If you were eligible for this study would you be willing to	Yes	No
Come to 2 face-to-face appointments each lasting 1 h?	40 (100%)	0
Come to the appointments after fasting for 12 h (only have water)?	40 (100%)	0
Allow a blood sample be taken by trained staff?	40 (100%)	0
Have your blood pressure measured by trained staff?	40 (100%)	0
Have your body muscle and fat measured by trained staff?	40 (100%)	0
Have your grip strength tested by trained staff?	39 (97.5%)	1 (2.5%)
Answer questionnaires/interviews based on your experiences?	40 (100%)	0
Have all the above tests measured by a man or woman?	40 (100%)	0
Be randomly put into any of the groups described in the study design for 12 weeks?	40 (100%)	0
Travel to a local gym to exercise 3 days per week (This would be supervised by a trained person)?	36 (90%)	4 (10%)
Change your diet to a healthier diet (high-protein Mediterranean style as described)?	39 (97.5%)	1 (2.5%)
Eat 2 high protein yogurts per day?	39 (97.5%)	1 (2.5%)
Fill out a food diary for 3 days to record what you have eaten?	40 (100%)	0
Have regular (weekly) contact by telephone or text message with a member of the research team to talk about how your exercise and diet is going?	40 (100%)	0

Data displayed as number and (percentage) of respondents who selected each answer option (e.g., 100% would represent that all this question’s respondents chose that option).

##### 3.2.1.3. Proposed recipes

**FIGURE 3:**
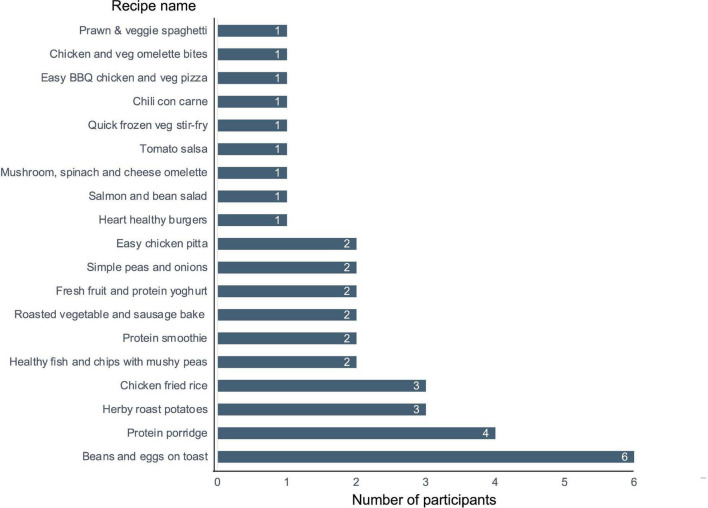
Recipes trialed by participants. Numbers presented represent the number of participants that trialed a specific recipe.

**FIGURE 4:**
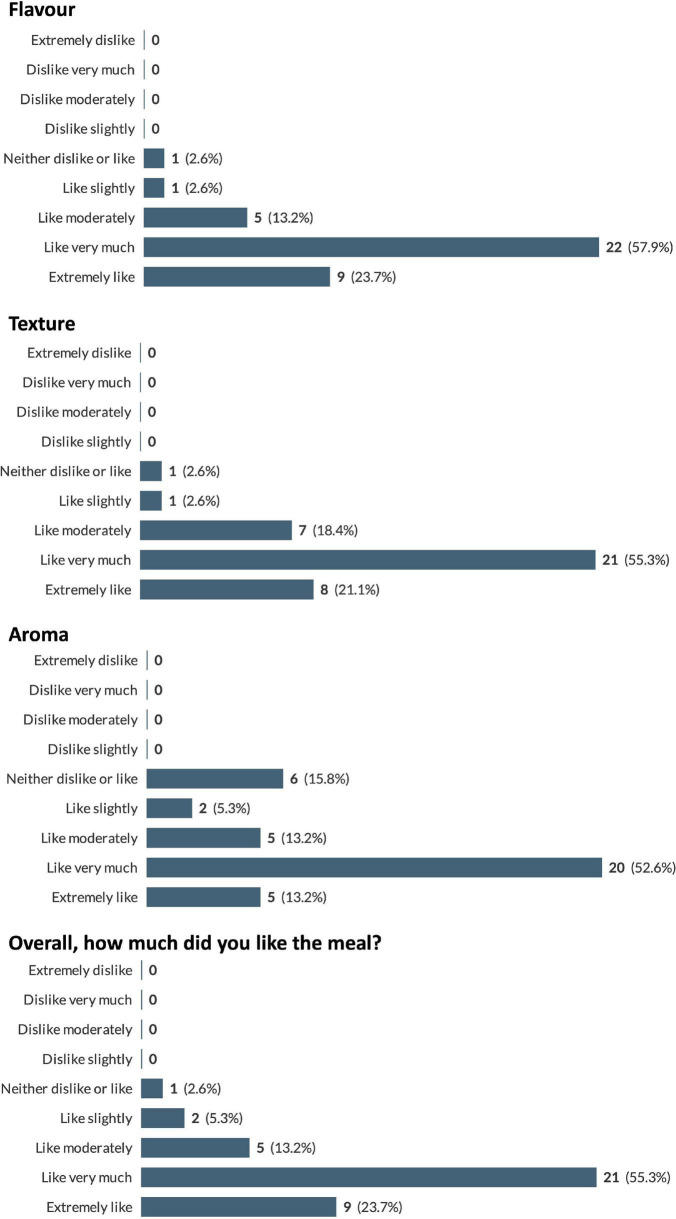
Pooled participant responses to questions relating to gustatory impressions of the proposed recipes. Displayed as number and (percentage) of responses to each answer option (e.g., 100% would represent that all this question’s responses chose that option).

**FIGURE 5:**
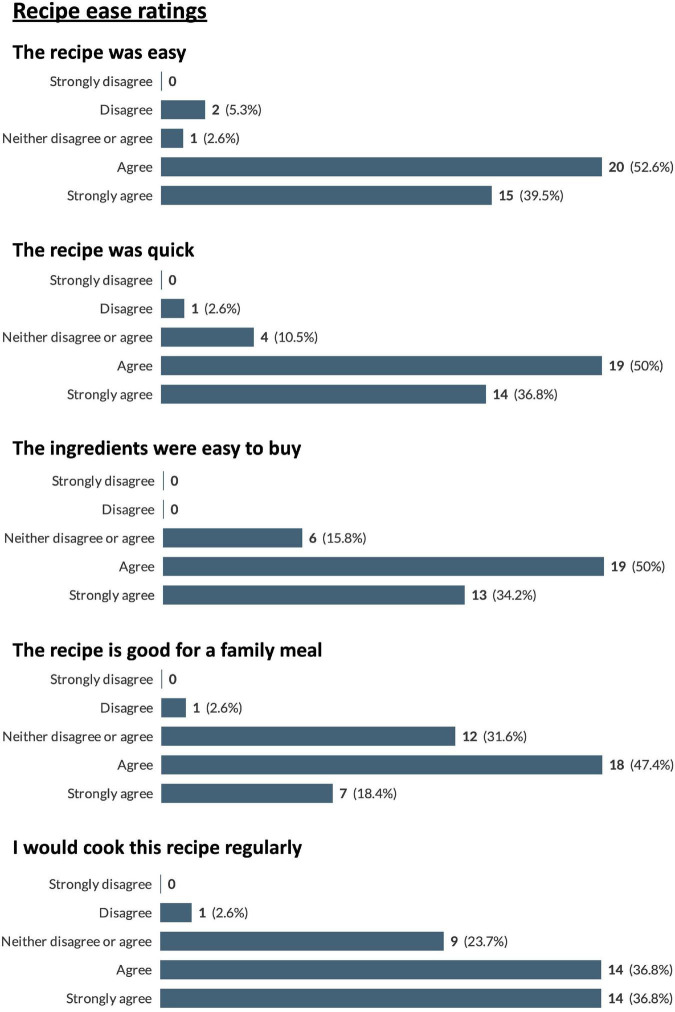
Pooled participant responses to questions relating to ease of preparation of the proposed recipes. Displayed as number and (percentage) of responses to each answer option (e.g., 100% would represent that all this question’s responses chose that option).

A total of 12 participants completed the recipe-related questionnaires. As each participant was instructed to try and provide feedback on as many recipes as they wished, a total of 38 responses were received. The recipes trialed, and the frequency of use of each recipe are presented in Figure 3. Due to the large number of recipes trialed by the participants it was decided to pool the results from the recipe-related questionnaires to give an overview of participants’ impressions of all the recipes trialed. A breakdown of participants’ gustatory ratings of the recipes can be seen in Figure 4. In general, the recipes were well received by the participants with 79% stating that, overall, they “liked very much” or “extremely liked” the recipes they trialed (Figure 4). Participants’ ratings of the ease/convenience of making the recipes are displayed in Figure 5. In general, 73.6% of respondents either agreed or strongly agreed that they would regularly make the recipe(s) they trialed.

##### 3.2.1.4. Proposed exercises

**FIGURE 6:**
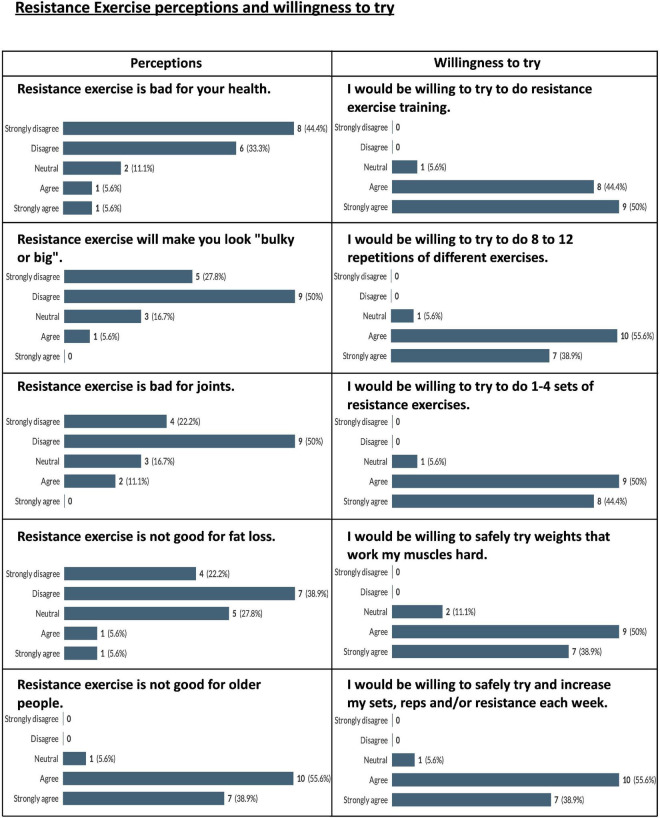
Pooled participant responses to questions relating to perceptions of and willingness to try resistance exercise. Displayed as number and (percentage) of responses to each answer option (e.g., 100% would represent that all this question’s responses chose that option).

A total of 18 participants completed the RE-related questionnaires. Results for participants’ perceptions of RE, in general are displayed in Figure 6. The majority of participants (61.1% and above) disagreed or strongly disagreed with a number of common negative perceptions of RE such as “resistance exercise will make you look bulky or big” and “resistance exercise is bad for joints” (Figure 6). However, 94.5% of participants agreed or strongly agreed that “Resistance exercise is not good for older people” (Figure 6). Participants responded positively to statements about their willingness to participate in the various aspects of the proposed RE intervention with 88.9% of participants agreeing or strongly agreeing with the various participation questions (Figure 6).

**FIGURE 7:**
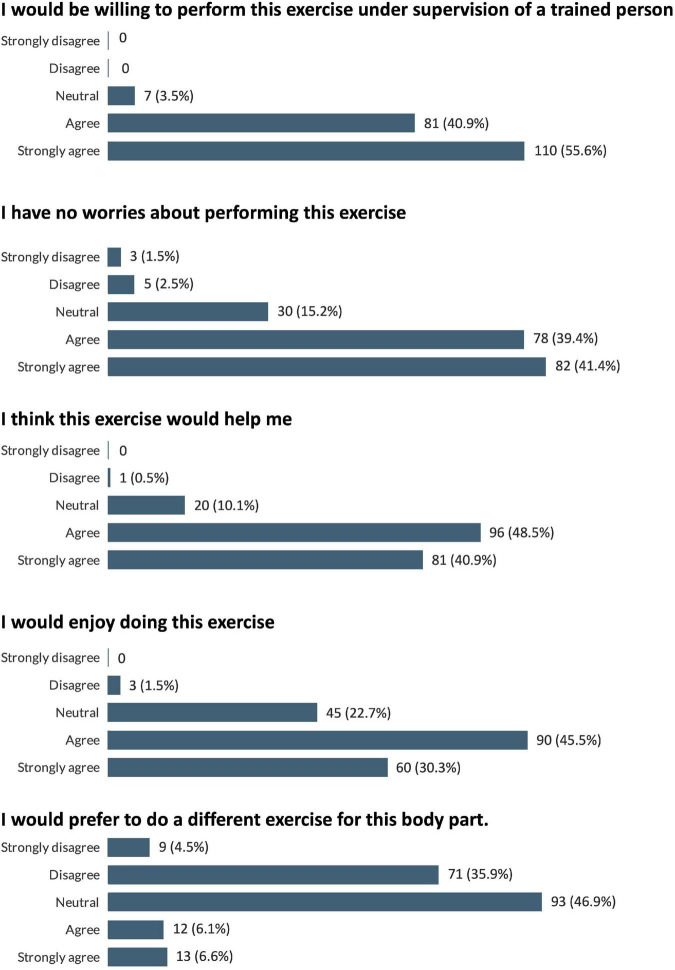
Pooled participant responses to questions relating to impressions of the proposed resistance exercises. Displayed as number and (percentage) of responses to each answer option (e.g., 100% would represent that all this question’s responses chose that option).

Participants also watched videos of the individual REs for the proposed intervention (leg press, Smith machine deadlift, machine chest press, machine row, machine shoulder press, lat pulldown, leg extension, hamstring curl, chest fly machine, horizontal cable row, and shoulder press machine). Due to the large number of exercise videos watched (*n* = 11) by the participants it was decided to pool the results from the RE-related questionnaires to give an overview of participants’ impressions of all the exercises viewed, and the results are displayed in Figure 7. In general, the exercises were well received by the participants with 96.5% of responses agreeing or strongly agreeing that they would be willing to perform the exercises under trained supervision, and 75.8% of responses agreeing or strongly agreeing that they would enjoy the exercises.

### 3.3. Qualitative results

#### 3.3.1. Proposed protocol

##### 3.3.1.1. Theme 1. “Pleasantly surprised”: Support for research and practical recommendations for improvements

Overall participants welcomed the concept of this research proposal. The research materials were considered appropriate and well explained. The resources were generally seen as a supportive reminder to prioritize their health.

A few practical recommendations for information formatting and providing hard copies of resources as opposed to digital versions, were suggested. Finally, some participants requested further information on the recommendations for heart health and how it relates to this to dietary requirements (Table 3).

**TABLE 3:** Quotes to support theme 1.

Supportive of research	Practical recommendations
*“Anything that refreshes your memory and helps put you in the right direction doing the right stuff is always good” (P1)*	*“Improved somewhat by perhaps a little bit more of an index, perhaps by adding a bit more nutritional information. And if it’s from my point of view as a standalone book, these sort of references to the rest of the cardiac rehab thing” (P6)*
*“Like many overweight people I have a history of dieting and trying different things. And so, my ideal scenario based on past experience*… *it sounds like that combined approach sounds very attractive” (P7)*	*“It was alright. It was simple. There was nothing difficult, well the things that I did. There was nothing difficult. I did change one or two things put in a little bit here and there to make it more personal.” (P3)*
*“I thought the instructions were easy to follow couldn’t fault this at all” (P5)*	*“On the front page, it says food and exercise for a healthier heart. But there’s nothing about exercise in the recipe book. So it is part of the prime trial, that’s fine. But as a standalone, it’s, it’s a bit confusing.” (P6)*
*“If you can come up with the ideal diet and exercise programme to help people recover. Yeah.” (P1)*	*“The introduction, it says the idea of this recipe book is to give you an idea of blah blah blah by following this particular way of eating, but it doesn’t actually tell you what this particular way of eating is, gives you lots of recipes. So I think, I don’t know how you’re going to use it” (P6)*
*“I was pleasantly surprised” (P7)*	*“Some inconsistency with some of the text in the recipe book” (P4)*
*“My point of view, which is from the point of view of a non-expert cook. It was it was very good” (P6)*	*“And sometimes I struggled to for me being an older person perhaps it might have been handy to have (a hard copy)” (P4)*
*“People need to know and find out how exercise, how diet affects an unhealthy or a healthy heart, and to do that you need to do all the things that you’re going to do (in the study)” (P3)*	
*“Mediterranean diet is admired for improving people’s wellbeing and longevity, and I haven’t selected that type of foods most of the time. Yeah.” (P4)*	

P, Participant.

##### 3.3.1.2. Theme 2: “I definitely would eat that” evaluation of the dietary approach

This theme is explained by an overall positive response to the proposed dietary intervention. Participants typically considered they were aware of, or already engaged in similar healthy eating practices, or had aspirations to do similar. Participants described how they made tweaks to personalize recipes, but overall recipes were “not difficult,” considered easy to follow, and often used “everyday” readily available ingredients. The adapted Mediterranean dietary intervention proposed was therefore considered acceptable and appropriate to recommend to this patient population.

In addition, this theme outlines participants’ queries and recognizes that participants offered practical recommendations particularly in relation to the dietary element of the intervention. For example, there were recommendations that the recipes should use European measurements as opposed to American cup measurements for the ingredients and the recipes could include more nutritional information (e.g., the calorie and macronutrient information). It is noteworthy that participants made “tweaks” to recipes to increase perceived “healthiness,” such as reducing the amount of fat or oil used within the ingredients list. These amendments related to their (mis)understanding of the dietary approach, and it might be helpful to offer participants further information on the development and background evidence-base upon which these dietary recipes are based (Table 4).

**TABLE 4:** Quotes to support theme 2.

Supportive of dietary approach and recipe book	Queries and amendments to dietary approach
*“Master some of these recipes. They were well written and simple to follow*…. *There was nothing in it that I was frightened of, or I wouldn’t have tried” (P6)*	*“It listed on there, sort of how many calories there were in each dish, because I’m diabetic as well. And I’d like to keep checking the calories. So that would you know if you pick up a packet in the supermarket, it tells you how many calories you’re eating.” (P6)*
*“The Pictures were very appealing. So I like to see what I’m supposed to be making I like to see what it’s supposed to look like. So having the picture and the one page instructions were an easy thing to do” (P7)*	*“I find confusing it just seems to me the recipes maybe originated in the States. So that a lot of the measurements. And I find that confusing because everybody’s got different cups” (P4)*
*“Was something that we would have normally done with a little bit of a twist on it.” (P3)*	*“I would prefer measurements of the tomatoes to be quantified. I don’t like the American cup measurements. It would be beneficial for me to have carbohydrate value too” (open response)*
*“Super easy, super easy. I’m always on the lookout for new recipes but these were most of the stuff we already had in, you know the basics, just normal stuff you have in the cupboard. I liked the way everything was on one page so you just print out the page” (P7)*	*“It seemed more than necessary and tasted a bit ‘greasy’ to me. Probably too much food and too much time for a breakfast meal, at least for me! Maybe better for dinner!” (open response)*
*“Most appealing to me, but also, probably, they seem like an easy thing to do. you could do it quickly” (P1)*	*“We might have tried that but we cut down on one or two things because they’re on the higher fat side. So most of most of the stuff we have is chicken- based. I like Fish but my wife doesn’t so we don’t have as much as we should do.” (P2)*
*“They weren’t expensive meals. Which I think is important.” (P5)*	*“More oily fish dishes might be appropriate.” (P4)*
*“And I try and eat relatively healthy. So it was interesting for me to see some of the things that I thought Oh, yes, I definitely would eat that.” (P4)*	*“Getting used to doing these things. I mean, they’re not terribly complex. But I found with trying to eat the right food. Now I have a problem with the high cholesterol” (P1)*
*“I tried a few of the recipes, I should go on using the recipes.” (P6)*	*“If it (recipe) says it takes 10- 15 minutes. You know it’s gonna be double that.” (P2)*
	*“A lot of people just don’t have the time these days for food prep. You know, especially on a day like this, it’s gorgeous over here in Manchester so you don’t want to spend like 3 hours in the kitchen.” (P2)*
	*“I was a bit concerned about how many calories would be in the sausages. So I left that one out.” (P6)*

P, Participant; open responses are from the quantitative questionnaires and cannot be attributed to a specific participant.

##### 3.3.1.3. Theme 3. “Finding that right balance” exercise at home

Participants acknowledged that home-based exercise was feasible though most suggested that they were not motivated or did not regularly engage in physical activity at home. There was a sense that participants typically referred to other people doing or being able to exercise at home, but little acknowledgment that this was relevant to them as individuals. There was a call for more detailed information on the benefits and need of RE in this patient group.

Participants also queried the concept of home-based exercise and typically considered an external “trip to the gym” as more motivational as it offers structure and social support. There were concerns raised regarding the safety of RE at home, with thoughts that having professionals who can monitor and offer instruction, being more appropriate. References to the exercise videos were positive overall, with reference to clear instructions, although again, respondents expressed a need for confirmation that a particular exercise was suitable to their individual health status. Further consideration on the practical “how to do” and “what to do” is warranted, alongside consideration for the role of social and motivational support for a future research trial in this patient group (Table 5).

**TABLE 5:** Quotes to support theme 3.

Sample evidence quotations for “Finding that right balance” exercise at home
*“More detail on the sort of exercise you’re talking about?” (P1)*
*“That’s a motivation thing. I mean you’ve got to be motivated to go to the gym anyway, its having the motivation after a hard day’s work, you got to come in and then find the motivation to do exercise at home. So unless you can find a way of motivating people, and the NHS have things like the couch potato to five k, where you can join in on social media to find other groups support to support you.” (P3)*
*“from a motivation perspective, actually going to the gym is better” (P3)*
*“I have a treadmill at home that I occasionally use when I’m too. I prefer to go out”” (P6)*
*“I go a couple of times a week to exercise in the gym only cardiovascular stuff, only static cycling, a few bits of weights. But I like to do it away from. I prefer doing it away from home. Rather than at home. I regard doing it at home as better than not doing it. but I’d rather to go out. it makes it a bit more special and gets me out of the door. But I do I do try to exercise.” (P6)*
*“If you’ve not got an instructor or, or somebody else in the gym that knows what you’re doing. So he can say, hey mate, you need to do this to improve this or that. If you’re doing it at home and doing it wrong. You could end up doing yourself more damage.” (P3)*
*“I think it’s hard for me to do that at home. But as I’m not going to the gym at the moment, I should be doing something.” (P4)*
*“I think one one commits better in a group than as an individual, you know, you think oh, I’ll just do five minutes.” (P5)*
*“Looks like a simple exercise and not overly difficult” (open response)*
*“Only concern is I could damage my back. It sounds as if this has been thought about!” (open response)*
*“Having had open heart surgery last August I would be a bit apprehensive of this exercise if the weight was too heavy as I wouldn’t want to exert too much pressure on the internal wound” (open response)*
*“I have some slight shoulder pain on my left side when extending my arm in this way. I’m happy to do the exercise but would take advice on whether it’s appropriate for me” (open response)*

P, Participant; open responses are from the quantitative questionnaires and cannot be attributed to a specific participant.

## 4. Discussion

To our knowledge, this is the first mixed-methods study to determine the acceptability of using a high-protein Mediterranean-style diet and RE protocol to improve lean mass, strength and cardiometabolic risk in a UK CR population. Both our quantitative and qualitative analyses highlight the recognition of the importance of, acceptability of and willingness to participate in the research protocol presented to the participants. However, a desire for clarification on certain aspects of the protocol’s diet and exercise components and requests for more personalized guidance relating to these components were also highlighted.

The proposed research protocol presented to participants was developed in collaboration with CR practitioners and a hospital-based service-users group (LHCH SURE group) to ensure the research proposal was both understandable and applicable to the end users. This was intended not only to make the research more acceptable but to improve potential participants “health literacy” in relation to the aims and methods of the protocol. Health literacy has been described as “the cognitive and social skills which determine the motivation and ability of individuals to gain access to, understand and use information in ways which promote and maintain good health” (47). According to the American Medical Association, “health literacy entails more than a patient being able to read written instructions; it requires the ability to comprehend and apply the information ascertained” (48). As such, ensuring the materials provided to participants improve health literacy related to an intervention should be considered a vital aspect of intervention design. The qualitative research presented here has highlighted several areas where the proposed intervention can be improved. The formatting/presentation of study materials has been highlighted with participants requesting, for example, inclusion of an index, provision of more nutritional information for recipes (calories, carbohydrate content etc.) for recipes and inclusion of more information related to the dietary pattern recommended in the intervention. Other suggestions included changes to/standardization of the measurements used for the recipes, particularly focused on avoiding the use of cup-measures (which are not commonly used in the UK).

Of particular note were comments from participants related to the quantity of oil used in some of the recipes with some participants reluctant to use so much oil when cooking. The Mediterranean dietary pattern is characterized by its use of olive oil as the primary culinary oil, which is believed to be partially responsible for some of the noted health benefits of this way of eating (49, 50). The inclusion of educational material explaining the potential health benefits of olive oil (and other aspects of the Mediterranean dietary pattern) may be useful to assuage any concerns participants may have regarding the use of olive oil. It should also be noted that the participants in this study only received the recipe booklet and not the full dietary guide for the proposed research intervention, which does contain such information. Of further note is the quantitative result that almost half of the participants in this study did not know or only somewhat knew what a “Mediterranean” diet was. The provision of such information in the participant guides/materials should be considered for future iterations of the intervention.

While 88.9% of participants agreed or strongly agreed with the various participation questions related to the RE intervention, in contrast, 94.5% of participants agreed or strongly agreed that “Resistance exercise is not good for older people.” This is another potential educational aspect that is worth elaborating on in future versions of the protocol. Resistance exercise has been shown to have multiple benefits for older adults including improving cardiometabolic risk markers, reducing measures of frailty and improving quality of life (51–53). It should also be noted that appropriately instructed and monitored RE is safe in older adults and even those with CVD (54). The provision of such information in an easy-to-understand format may be useful in encouraging participation in such interventions.

Participants also commented that the act of going to a gym to perform exercise may be more beneficial and is a concept worthy of further exploration. Performing in-person/gym-based exercise may increase the likelihood of vicarious experiences (observing others be successful) and verbal persuasion (verbal cues and/or feedback that may encourage success) (55), which may lead to greater self-efficacy. Self-efficacy theory proposes that a favorable impression of one’s results can help to encourage individuals to adhere to endeavors such as exercise (56). As such, the perception that results of in-person/gym-based exercise may be more beneficial may help individuals adhere to exercise programs such as CR (57), and accordingly, the benefits of such exercise should be elaborated on in any material/instruction provided to participants. Providing of such information along with contact with peers and CR exercise providers in in-person/gym-based settings might help encourage self-efficacy (58, 59) and exercise maintenance.

### 4.1. Strengths and limitations

This study presents a number of strengths and limitations. A particular strength of this study is the high proportion of female participants (50%), which is notably higher than the proportion of female CR attendance in England, which ranges from only 15 to 38% (60). Another strength of this study is the use of the mixed methodology approach to seeking feedback and engagement for this intervention. This offers a safe forum for participants to express their experiences and not be biased by researcher expectations. As such, feedback and analysis can be considered more reflective of participants own perceptions. The general agreement of both quantitative and qualitative results in terms of the acceptability of the proposed intervention is also a strength of this research.

The majority of participants were of White British ethnicity (90%), and this is broadly considered a representative sample of CR participants in the UK, based on a recently published report of CR demographics that reported 83.8% of participants as white (60). However, it is noteworthy that the findings may not be representative of the diverse ethnic population of the UK as a whole or globally and as such further engagement and exploration of the intervention in a more diverse patient population group is warranted. It should be noted that this study does not have data on the socio-economic status or household income of the participants. Without such information these data cannot determine if the proposed recipes and exercises would be acceptable in different socio-economic groups and as such, further research is warranted in these population groups. Furthermore, as the majority of participants had class 1 obesity research with larger numbers of participants in more diverse BMI classifications may be beneficial for tailoring the diet and exercise guidelines.

A further limitation is that participants had the freedom to choose which recipes to make, which may have biased the results of their ratings of the recipes, as participants would naturally choose recipes they expect to agree with their palate and personal tastes. Finally, participants were recruited from RftF who are likely to be a subset of people/patients very willing to participate in research and may not be representative of the wider clinical population in the UK.

## 5. Conclusion

This mixed-methods study found that the proposed high-protein, Mediterranean-style diet, and resistance exercise protocols for cardiac rehabilitation participants were generally found to be acceptable, with a high degree of willingness to participate from potentially eligible participants. Several potential areas of improvement were highlighted, particularly in regards to clarification around the benefits of the diet and exercise components and provision of more comprehensive information in participant-facing guides/documents. This information will be vital for improving future iterations of the proposed intervention protocol to help ensure acceptance and compliance in the target population, helping to increase the likelihood of positive health outcomes.

## Data availability statement

The datasets generated in this study are not publicly available due to privacy and ethical reasons but are available from the corresponding author on reasonable request. Raw qualitative data have been included as evidence via extracted quotes from verbatim transcripts as samples of evidence. Full transcript release has not received ethical approval or participant consent. For further study details, please contact the corresponding author. The authors confirm that the data supporting the findings of this study are available within the article.

## Ethics statement

The studies involving human participants were reviewed and approved by National Health Service North West–Greater Manchester East Research Ethics Committee. The patients/participants provided their written informed consent to participate in this study.

## Author contributions

RK, DM, ID, FP, and TB conceived and designed the study. RK and DM carried out data collection. RK and LN performed data analysis and wrote the first draft of the manuscript. All authors critically revised all versions of the manuscript, read, and approved the final manuscript.

## Footnotes

1
https://www.lhch.nhs.uk/research/sure-group/

2
https://www.researchforthefuture.org/

3
https://www.nihr.ac.uk/explore-nihr/support/clinical-research-network.htm

4
https://www.onlinesurveys.ac.uk/

## Supplementary material

Supplementary Figure 1 Study research plan.

## References

[1] Tsao C Aday A Almarzooq Z Alonso A Beaton A Bittencourt M Heart disease and stroke statistics—2022 update: a report from the American heart association. *Circulation.* (2022) 145:e153–639. 35078371 10.1161/CIR.0000000000001052

[2] National Institute for Health and Care Excellence. *Myocardial infarction: cardiac rehabilitation and prevention of further cardiovascular disease.* London: National Institute for Health and Care Excellence (2013). 31891465

[3] British Association for Cardiovascular Prevention and Rehabilitation,. BACPR standards and core components for cardiovascular disease prevention and rehabilitation. Heart. (2017).

[4] Anderson L Thompson D Oldridge N Zwisler A Rees K Martin N Exercise-based cardiac rehabilitation for coronary heart disease. *Cochrane Database Syst Rev.* (2016) 2016:Cd001800. 10.1002/14651858.CD001800.pub3 26730878 PMC6491180

[5] Sagar V Davies E Briscoe S Coats A Dalal H Lough F Exercise-based rehabilitation for heart failure: systematic review and meta-analysis. *Open Heart.* (2015) 2:e000163. 10.1136/openhrt-2014-000163 25685361 PMC4316592

[6] Lavie C Milani R. Adverse psychological and coronary risk profiles in young patients with coronary artery disease and benefits of formal cardiac rehabilitation. *Arch Intern Med.* (2006) 166:1878–83. 10.1001/archinte.166.17.1878 17000945

[7] Prado C Wells J Smith S Stephan B Siervo M. Sarcopenic obesity: a critical appraisal of the current evidence. *Clin Nutr.* (2012) 31:583–601. 10.1016/j.clnu.2012.06.010 22809635

[8] Atkins J Whincup P Morris R Lennon L Papacosta O Wannamethee S. Sarcopenic Obesity and risk of cardiovascular disease and mortality: a population-based cohort study of older men. *J Am Geriatr Soc.* (2014) 62:253–60. 10.1111/jgs.12652 24428349 PMC4234002

[9] Gusmao-Sena M Curvello-Silva K Barreto-Medeiros J Da-Cunha-Daltro C. Association between sarcopenic obesity and cardiovascular risk: where are we? *Nutr Hosp.* (2016) 33:592. 10.20960/nh.592 27759996

[10] Lavie C De Schutter A Patel D Romero-Corral A Artham S Milani R. Body composition and survival in stable coronary heart disease: impact of lean mass index and body fat in the “obesity paradox”. *J Am Coll Cardiol.* (2012) 60:1374–80. 10.1016/j.jacc.2012.05.037 22958953

[11] Lavie C Osman A Milani R Mehra M. Body composition and prognosis in chronic systolic heart failure: the obesity paradox. *Am J Cardiol.* (2003) 91:891–4. 10.1016/S0002-9149(03)00031-6 12667583

[12] Romero-Corral A Montori V Somers V Korinek J Thomas R Allison T Association of bodyweight with total mortality and with cardiovascular events in coronary artery disease: a systematic review of cohort studies. *Lancet.* (2006) 368:666–78. 10.1016/s0140-6736(06)69251-9 16920472

[13] Schrager M Metter E Simonsick E Ble A Bandinelli S Lauretani F Sarcopenic obesity and inflammation in the inchianti study. *J Appl Physiol.* (2007) 102:919–25. 10.1152/japplphysiol.00627.2006 17095641 PMC2645665

[14] Fox C Massaro J Hoffmann U Pou K Maurovich-Horvat P Liu C Abdominal visceral and subcutaneous adipose tissue compartments: association with metabolic risk factors in the framingham heart study. *Circulation.* (2007) 116:39–48. 10.1161/circulationaha.106.675355 17576866

[15] Medina-Urrutia A Posadas-Romero C Posadas-Sanchez R Jorge-Galarza E Villarreal-Molina T Gonzalez-Salazar Mdel C Role of adiponectin and free fatty acids on the association between abdominal visceral fat and insulin resistance. *Cardiovasc Diabetol.* (2015) 14:20. 10.1186/s12933-015-0184-5 25849597 PMC4332423

[16] Ebbert J Jensen M. Fat depots, free fatty acids, and dyslipidemia. *Nutrients.* (2013) 5:498–508. 10.3390/nu5020498 23434905 PMC3635208

[17] Rubio-Ruiz M Guarner-Lans V Pérez-Torres I Soto M. Mechanisms underlying metabolic syndrome-related sarcopenia and possible therapeutic measures. *Int J Mol Sci.* (2019) 20:647. 10.3390/ijms20030647 30717377 PMC6387003

[18] Lee S Tung H Liu C Chen L. Physical activity and sarcopenia in the geriatric population: a systematic review. *J Am Med Dir Assoc.* (2018) 19:378–83. 10.1016/j.jamda.2018.02.003 29580886

[19] Steell L Ho F Sillars A Petermann-Rocha F Li H Lyall D Dose-response associations of cardiorespiratory fitness with all-cause mortality and incidence and mortality of cancer and cardiovascular and respiratory diseases: the Uk biobank cohort study. *Br J Sports Med.* (2019) 53:1371–8. 10.1136/bjsports-2018-099093 30796106

[20] Sillars A Celis-Morales C Ho F Petermann F Welsh P Iliodromiti S Association of fitness and grip strength with heart failure: findings from the Uk biobank population-based study. *Mayo Clin Proc.* (2019) 94:2230–40. 10.1016/j.mayocp.2019.04.041 31685151

[21] HouLLeiYLiXHuoCJiaXYangJ Effect of protein supplementation combined with resistance training on muscle mass, strength and function in the elderly: a systematic review and meta-analysis. *J Nutr Health Aging.* (2019) 23:451–8. 10.1007/s12603-019-1181-2 31021362

[22] Vanhees L Rauch B Piepoli M van Buuren F Takken T Borjesson M Importance of characteristics and modalities of physical activity and exercise in the management of cardiovascular health in individuals with cardiovascular disease (Part Iii). *Eur J Prev Cardiol.* (2012) 19:1333–56. 10.1177/2047487312437063 22637740

[23] Piepoli M Hoes A Agewall S Albus C Brotons C Catapano A 2016 European guidelines on cardiovascular disease prevention in clinical practice: the sixth joint task force of the European society of cardiology and other societies on cardiovascular disease prevention in clinical practice (constituted by representatives of 10 societies and by invited experts)developed with the special contribution of the European association for cardiovascular prevention & rehabilitation (Eacpr). *Eur Heart J.* (2016) 37:2315–81. 10.1093/eurheartj/ehw106 27222591 PMC4986030

[24] Price K Gordon B Bird S Benson A. A review of guidelines for cardiac rehabilitation exercise programmes: is there an international consensus? *Eur J Prev Cardiol.* (2016) 23:1715–33. 10.1177/2047487316657669 27353128

[25] Khadanga S Savage P Ades P. Resistance training for older adults in cardiac rehabilitation. *Clin Geriatr Med.* (2019) 35:459–68. 10.1016/j.cger.2019.07.005 31543178 PMC8237336

[26] Deanfield J Simpson I Bradbury K Fox K Boon N Winocour P Joint British societies’ consensus recommendations for the prevention of cardiovascular disease (Jbs3). *Heart.* (2014) 100:1–67. 10.1136/heartjnl-2014-305693 24667225

[27] National Institute for Health Care Excellence. *Myocardial infarction: cardiac rehabilitation and prevention of further cardiovascular disease.* London: National Institute for Health Care Excellence (2013). 31891465

[28] Iestra J Knoops K Kromhout D de Groot L Grobbee D van Staveren W. Lifestyle, mediterranean diet and survival in european post-myocardial infarction patients. *Eur J Cardiovasc Prev Rehabil.* (2006) 13:894–900. 10.1097/01.hjr.0000201517.36214.ba 17143120

[29] Panagiotakos D Notara V Kouvari M Pitsavos C. The mediterranean and other dietary patterns in secondary cardiovascular disease prevention: a review. *Curr Vasc Pharmacol.* (2016) 14:442–51. 10.2174/1570161114999160719104731 27456104

[30] Trichopoulou A Bamia C Norat T Overvad K Schmidt E Tjonneland A Modified mediterranean diet and survival after myocardial infarction: the epic-elderly study. *Eur J Epidemiol.* (2007) 22:871–81. 10.1007/s10654-007-9190-6 17926134

[31] Trichopoulou A Bamia C Trichopoulos D. Mediterranean diet and survival among patients with coronary heart disease in Greece. *Arch Intern Med.* (2005) 165:929–35. 10.1001/archinte.165.8.929 15851646

[32] de Lorgeril M Salen P Martin J Monjaud I Delaye J Mamelle N. Mediterranean diet, traditional risk factors, and the rate of cardiovascular complications after myocardial infarction – final report of the lyon diet heart study. *Circulation.* (1999) 99:779–85. 10.1161/01.Cir.99.6.779 9989963

[33] Linan Pinto M Pinto R Charneca S Vasques J Lemos Pires M Borges M Body composition, lipid profile and mediterranean diet adherence in cardiovascular disease patients attending a long-term exercise-based cardiac rehabilitation program during covid-19 pandemic. *Eur J Prev Cardiol.* (2021) 28(Suppl. 1):zwab061.187. 10.1093/eurjpc/zwab061.187

[34] Vanzella L Rouse V Ajwani F Deilami N Pokosh M Oh P Barriers and facilitators to participant adherence of dietary recommendations within comprehensive cardiac rehabilitation programmes: a systematic review. *Public Health Nutr.* (2021) 24:4823–39. 10.1017/S1368980021002962 34344495 PMC11082819

[35] Campbell N Murray E Darbyshire J Emery J Farmer A Griffiths F Designing and evaluating complex interventions to improve health care. *BMJ.* (2007) 334:455–9. 10.1136/bmj.39108.379965.BE 17332585 PMC1808182

[36] Yardley L Ainsworth B Arden-Close E Muller I. The person-based approach to enhancing the acceptability and feasibility of interventions. *Pilot Feasibility Stud.* (2015) 1:37. 10.1186/s40814-015-0033-z 27965815 PMC5153673

[37] McCullough D Kirwan R Butler T Perez de Heredia F Thijssen D Lip G Feasibility of a high-protein mediterranean-style diet and resistance exercise in cardiac rehabilitation patients with sarcopenic obesity (Primer): study protocol for a randomised control trial. *Clin Nutr ESPEN.* (2021) 45:492–8. 10.1016/j.clnesp.2021.08.001 34620360

[38] Creswell J Clark V. *Designing and conducting mixed methods research.* Thousand Oaks, CA: Sage publications (2017).

[39] Einarson T Acs A Ludwig C Panton U. Prevalence of cardiovascular disease in type 2 diabetes: a systematic literature review of scientific evidence from across the world in 2007–2017. *Cardiovasc Diabetol.* (2018) 17:83. 10.1186/s12933-018-0728-6 29884191 PMC5994068

[40] World Medical Association. *WMA declaration of Helsinki – Ethical principles for medical researc involving human subjects.* Ferney-Voltaire: World Medical Association (2018).

[41] R Core Team (2021). *R: A language and environment for statistical computing*. Vienna, Austria: R Foundation for Statistical Computing. Available online at: https://www.R-project.org/

[42] Thorne S Kirkham S MacDonald-Emes J. Interpretive description: a noncategorical qualitative alternative for developing nursing knowledge. *Res Nurs Health.* (1997) 20:169–77. 10.1002/(SICI)1098-240X(199704)20:2<169::AID-NUR9>3.0.CO;2-I 9100747

[43] Corbin J Strauss A. *Basics of qualitative research: techniques and procedures for developing grounded theory.* Thousand Oaks, CA: Sage publications (2014).

[44] Noble H Smith J. Issues of validity and reliability in qualitative research. *Evid Based Nurs.* (2015) 18:34–5. 25653237 10.1136/eb-2015-102054

[45] Heale R Forbes D. Understanding triangulation in research. *Evid Based Nurs.* (2013) 16:98. 10.1136/eb-2013-101494 23943076

[46] Reynolds J Kizito J Ezumah N Mangesho P Allen E Chandler C. Quality assurance of qualitative research: a review of the discourse. *Health Res Policy Syst.* (2011) 9:1–10. 22182674 10.1186/1478-4505-9-43 PMC3267652

[47] Nutbeam D. Health literacy as a public health goal: a challenge for contemporary health education and communication strategies into the 21st century. *Health Promot Int.* (2000) 15:259–67.

[48] Parker R Williams M Weiss B Baker D Davis T Doak C Health literacy-report of the council on scientific affairs. *JAMA J Am Med Assoc.* (1999) 281:552–7. 10022112

[49] Trichopoulou A Martínez-González M Tong T Forouhi N Khandelwal S Prabhakaran D Definitions and potential health benefits of the mediterranean diet: views from experts around the world. *BMC Med.* (2014) 12:112. 10.1186/1741-7015-12-112 25055810 PMC4222885

[50] D’Alessandro A De Pergola G. The mediterranean diet: its definition and evaluation of a priori dietary indexes in primary cardiovascular prevention. *Int J Food Sci Nutr.* (2018) 69:647–59. 10.1080/09637486.2017.1417978 29347867

[51] McLeod K Jones M Thom J Parmenter B. Resistance training and high-intensity interval training improve cardiometabolic health in high risk older adults: a systematic review and meta-anaylsis. *Int J Sports Med.* (2022) 43:206–18. 34320660 10.1055/a-1560-6183

[52] Bray N Smart R Jakobi J Jones G. Exercise prescription to reverse frailty. *Appl Physiol Nutr Metab.* (2016) 41:1112–6. 27649859 10.1139/apnm-2016-0226

[53] Hart P Buck D. The effect of resistance training on health-related quality of life in older adults: systematic review and meta-analysis. *Health Promot Perspect.* (2019) 9:1. 10.15171/hpp.2019.01 PMC6377696 30788262

[54] Kirkman D Lee D Carbone S. Resistance exercise for cardiac rehabilitation. *Prog Cardiovasc Dis.* (2022) 70:66–72. 10.1016/j.pcad.2022.01.004 35122871 PMC8930531

[55] Bandura A. Self-Efficacy: toward a unifying theory of behavioral change. *Psychol Rev.* (1977) 84:191. 10.1037//0033-295x.84.2.191 847061

[56] Desharnais R Bouillon J Godin G. Self-efficacy and outcome expectations as determinants of exercise adherence. *Psychol Rep.* (1986) 59:1155–9. 10.2466/pr0.1986.59.3.1155

[57] Kwasnicka D Dombrowski S White M Sniehotta F. Theoretical explanations for maintenance of behaviour change: a systematic review of behaviour theories. *Health Psychol Rev.* (2016) 10:277–96. 10.1080/17437199.2016.1151372 26854092 PMC4975085

[58] Jackson D. How personal trainers can use self-efficacy theory to enhance exercise behavior in beginning exercisers. *Strength Cond J.* (2010) 32:67–71.

[59] McAuley E Jerome G Marquez D Elavsky S Blissmer B. Exercise self-efficacy in older adults: social, affective, and behavioral influences. *Ann Behav Med.* (2003) 25:1. 10.1207/S15324796ABM2501_01 12581930

[60] Rehabilitation NAoC. *National audit of cardiac rehabilitation (NACR) quality and outcomes report 2019.* British Heart Foundation. (2019).

