# Supplementary material for: Acceptability of a high-protein Mediterranean-style diet and resistance exercise protocol for cardiac rehabilitation patients: Involving service users in intervention design using a mixed-methods participatory approach
Source: Front Nutr. 2023 Feb 14;10:1043391. doi: 10.3389/fnut.2023.1043391 (PMC9970995; doi:10.3389/fnut.2023.1043391)

Research Plan:

Heart disease is responsible for 1 in 4 deaths in the UK. The risk of heart disease can be increased by many causes. After a cardiac event such as a heart attack, people attend cardiac rehab to stop the disease getting worse. Normally for cardiac rehab, patients are helped to lose weight by eating fewer fatty foods and sugar and go to aerobic exercise sessions. The aerobic exercise sessions involve raising your heart rate for 30-45 minutes by doing exercises such as cycling to improve fitness.

Some people who are at a higher risk of death from heart disease have a normal body weight. Some people have less muscle and more fat than normal, and we believe that these patients don't look overweight – this is known as "sarcopenic obesity" (sarcopenia meaning loss of muscle, and obesity, excessive body fat). Loss of muscle is normal as we get older, but it might put patients at further risk of heart attacks.

Because of this it might be better to change to a diet and exercise programme that helps to improve muscle size and strength and blood markers of future risk of heart disease. If we are correct, our research could lead to improvements in current cardiac rehab (CR), meaning people will be less likely to have another cardiac event (like a heart attack).

To do this study, patients will need to meet the following requirements:

- must have been referred to cardiac rehab
- must be willing and able to do resistance exercise (exercise with weights)
- must be willing and able to eat dairy products (like yoghurt)
- must not have chronic kidney disease,
- must not have a congenital (from birth) or drug/alcohol-related heart condition.

The healthy diet we would ask cardiac rehab patients to follow is a a high-protein Mediterranean-style. This just means:

- eating **more** fruit and vegetables,
- **eating fewer** commercial pastries, and refined carbohydrate foods (white bread, white rice, white pasta)
- **eating more** wholegrains (wholegrain bread, rice and pasta),
- **replacing** butter and margarine with **olive oil** in some meals and dishes,
- **reducing** fatty meat and **eating more** lean meat (chicken), fish, and legumes (peas, beans, lentils),
- **eating more** high-protein, low fat foods, such as low-fat dairy (participants will be provided with 2 high-protein yoghurts to eat each day).

A trained person will give out recipes and guidance on how to make these affordable, healthy and tasty meals.

The exercise we would ask people to do is called resistance exercise. This means working your muscles hard to push or pull a light or heavy weight. Machines or weights can be used to do this. This type of exercise helps make muscles stronger and healthier. A trained person would safely guide the participant through the exercises 3 times per week.

To find out of if this diet and resistance is exercise programme is better for health, we would need to measure eligible patients at the start and end of a 12-week period. At the start we will put participants into 1 of 4 groups.

1 – Normal Cardiac Rehab (CR) diet and aerobic exercise (treadmills, rowing machines, elliptical trainers)

2 – High-protein Mediterranean style diet and normal CR aerobic exercise.

3 – Normal CR diet and resistance exercise (machines and weights).

4 – High-protein Mediterranean style diet and resistance exercise.

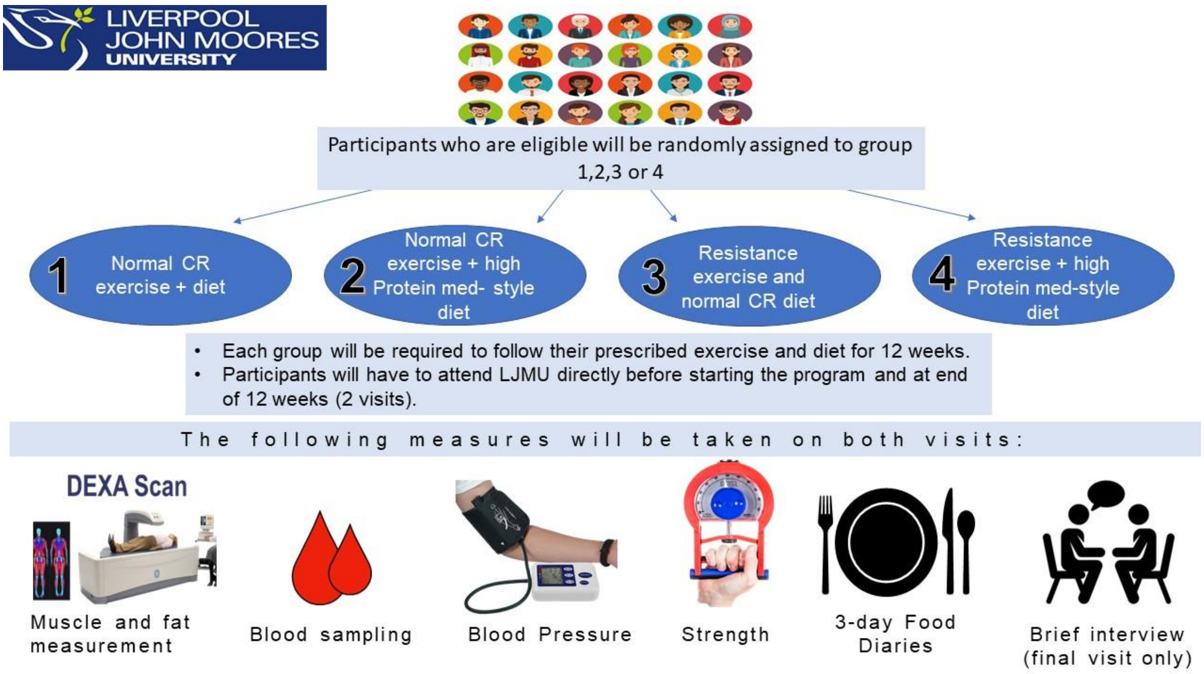

Supplement: Supplementary Figure 1 — Study research plan. [file Data_Sheet_1.zip › Supplementary figure 1 RESEARCH PLAN.pdf]
