# Supplementary material for: Acceptability of a high-protein Mediterranean-style diet and resistance exercise protocol for cardiac rehabilitation patients: Involving service users in intervention design using a mixed-methods participatory approach
Source: Front Nutr. 2023 Feb 14;10:1043391. doi: 10.3389/fnut.2023.1043391 (PMC9970995; doi:10.3389/fnut.2023.1043391)
Supplement: Supplementary Figure 1 — Study research plan. [file Data_Sheet_1.zip › Supplementary file 1 Recipes.pdf]

The PRiME Trial

Food and Exercise for a Healthier Heart!

EASY RECIPES

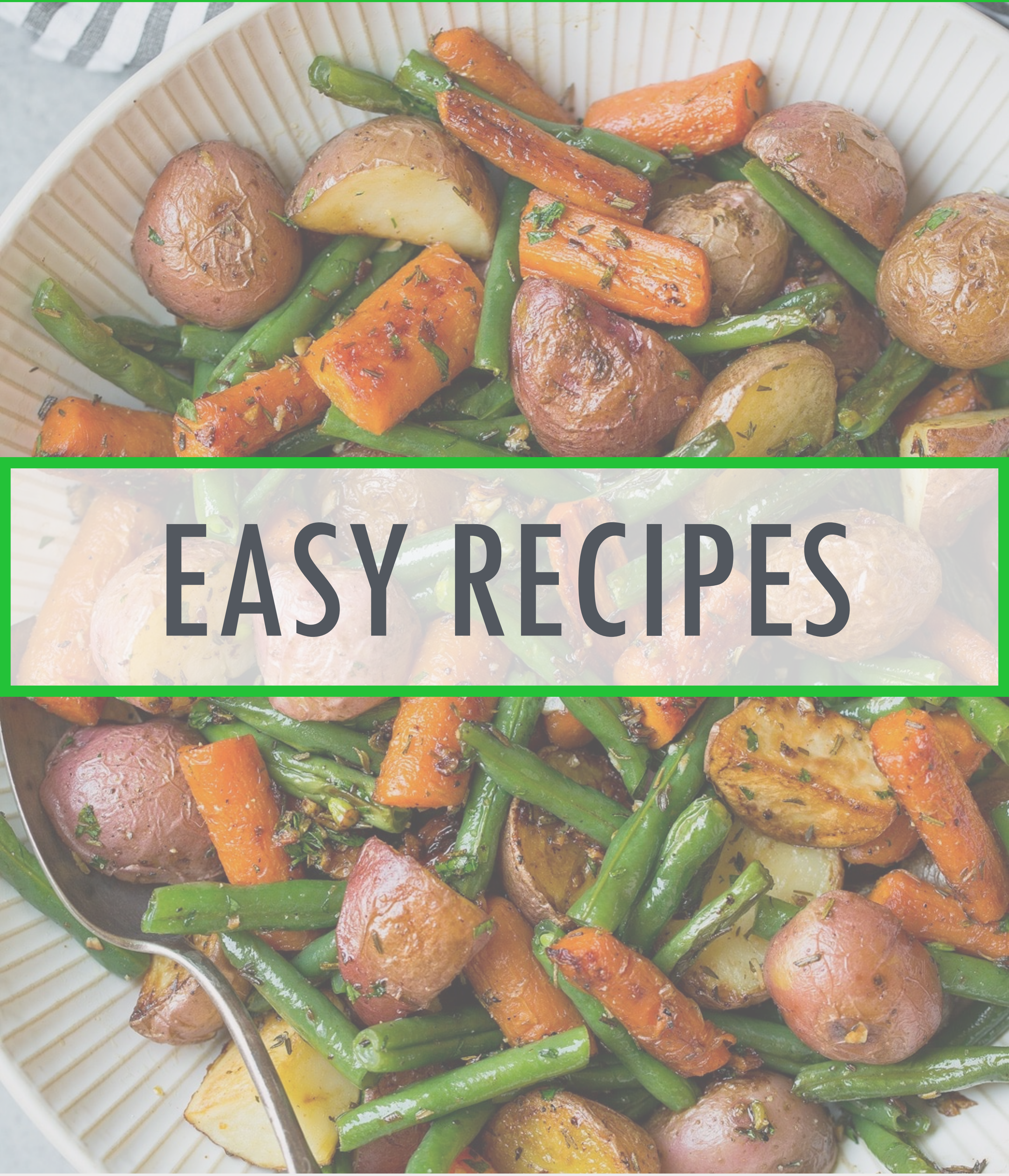

Contents

A word on recipes 1

Breakfast 2

Lunch & Salads 14

Vegetables, Sauces & Sides 22

Fish & Seafood 40

Main Meals 50

Desserts 76

The Ultimate Lazy Meal Plan 82

Sneak More Veggies into Your Meals 84

A WORD ON RECIPES

The idea of this recipe book is to give you an idea of all the amazing foods you can eat by following this particular way of eating.

The recipes are aimed at making it easier for you to follow this way of eating and to do that we don't want you to feel that you have to follow the recipes to the letter. One of the great things about food and cooking is that you can experiment yourself with different combinations to make a recipe that suits your taste buds.

Feel free to swap around different vegetables for vegetables, proteins for proteins and seasonings for seasonings. You could create your own kitchen masterpiece (in which case, let us know and we can pass on the recipe to others).

Above all else, enjoy the experience of cooking and the amazing food you make.

PROTEIN PORRIDGE

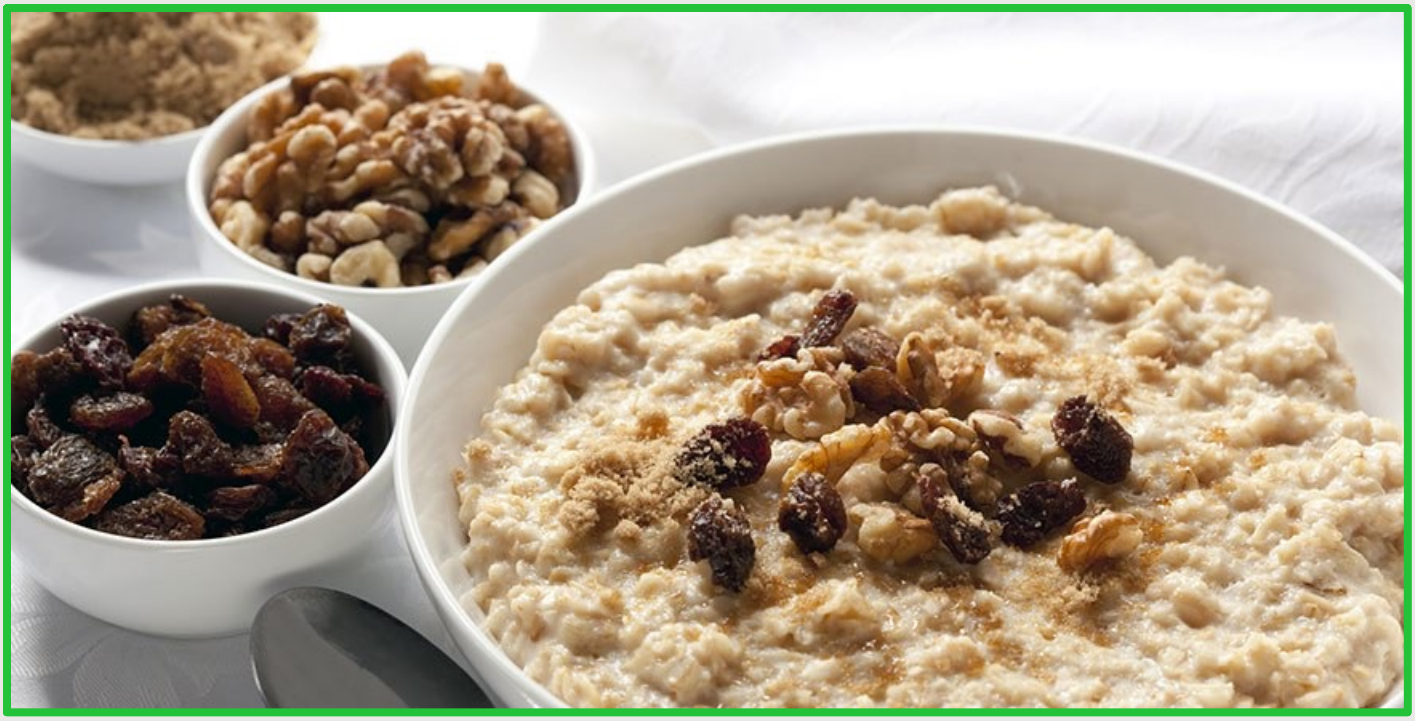

PROTEIN PORRIDGE

INGREDIENTS

- 30g porridge oats
- 200ml semi-skimmed milk
- 125g fat-free quark
- 20g raisins
- 30g almonds (or nuts of choice) (chopped)
- Granulated artificial sweetener (to taste)
- Ground cinnamon or cloves to taste

MAKES 1 SERVING
32g of Protein/serving

INSTRUCTIONS

1. Add the milk, oats and raisins to a microwavable bowl and microwave on high for 2 minutes
2. Once cooked, add the quark, nuts and sweetener and cinnamon. Mix well.
3. Eat straight away.

BEANS & EGGS ON TOAST

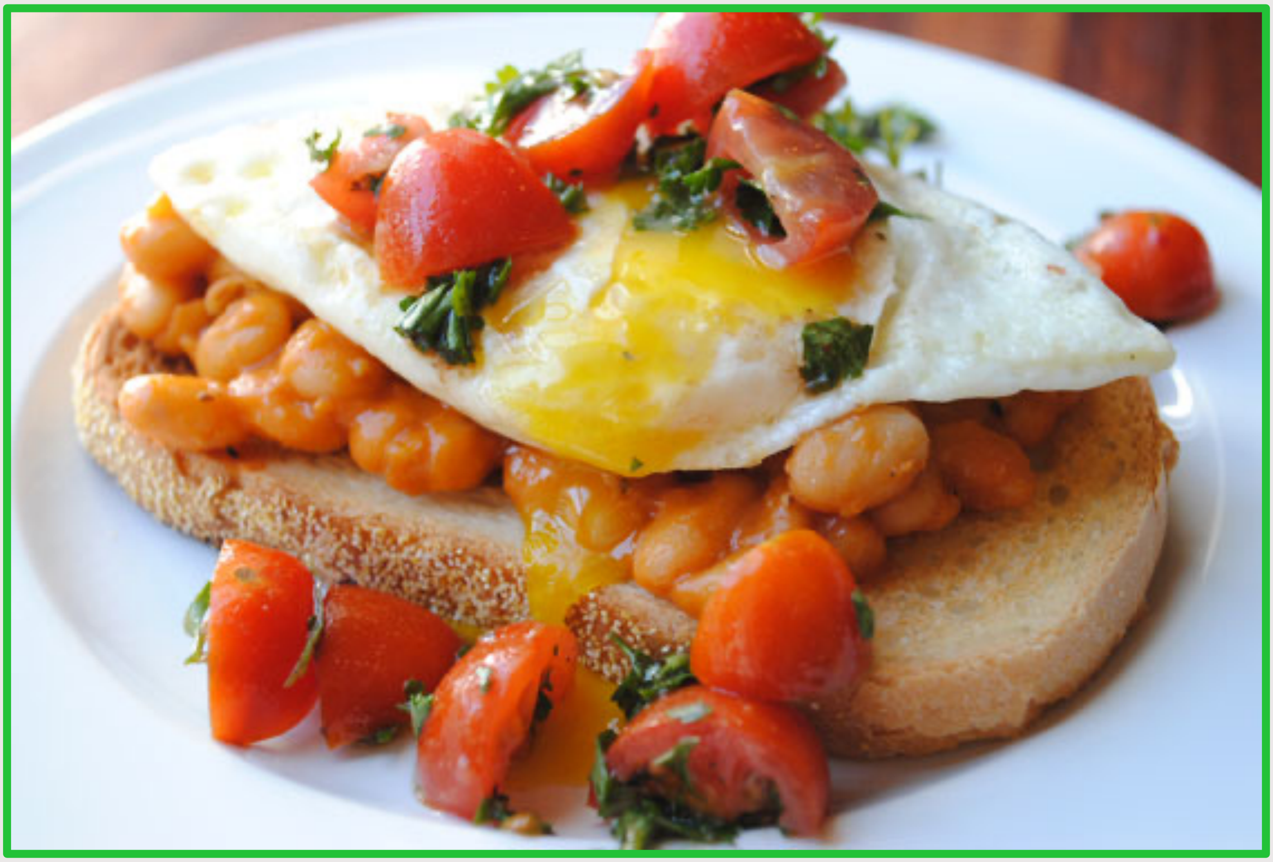

BEANS & EGGS ON TOAST

INGREDIENTS

- 2 large eggs
- ½ a can baked beans in tomato sauce
- 1 tsp. olive oil
- 2 medium tomatoes or 4-5 cherry tomatoes (halved)
- 1 tbsp. extra virgin olive oil
- 1 slice of wholegrain bread
- Garlic powder
- Salt and black pepper

MAKES 1 SERVING

27g of Protein/serving

INSTRUCTIONS

1. Preheat the oven to 200 degrees C
2. In a bowl, mix the tomatoes, olive oil, salt, pepper and garlic powder.
3. Place on an oven tray and bake for 10 minutes until softened
4. Heat the beans in a bowl in the microwave.
5. Fry the eggs in a fry pan with olive oil.
6. Toast the bread.
7. Place the bread onto a plate, top with the beans and then the eggs.
8. Serve the roasted tomatoes on the side.

CHICKEN & VEG OMELETTE BITES

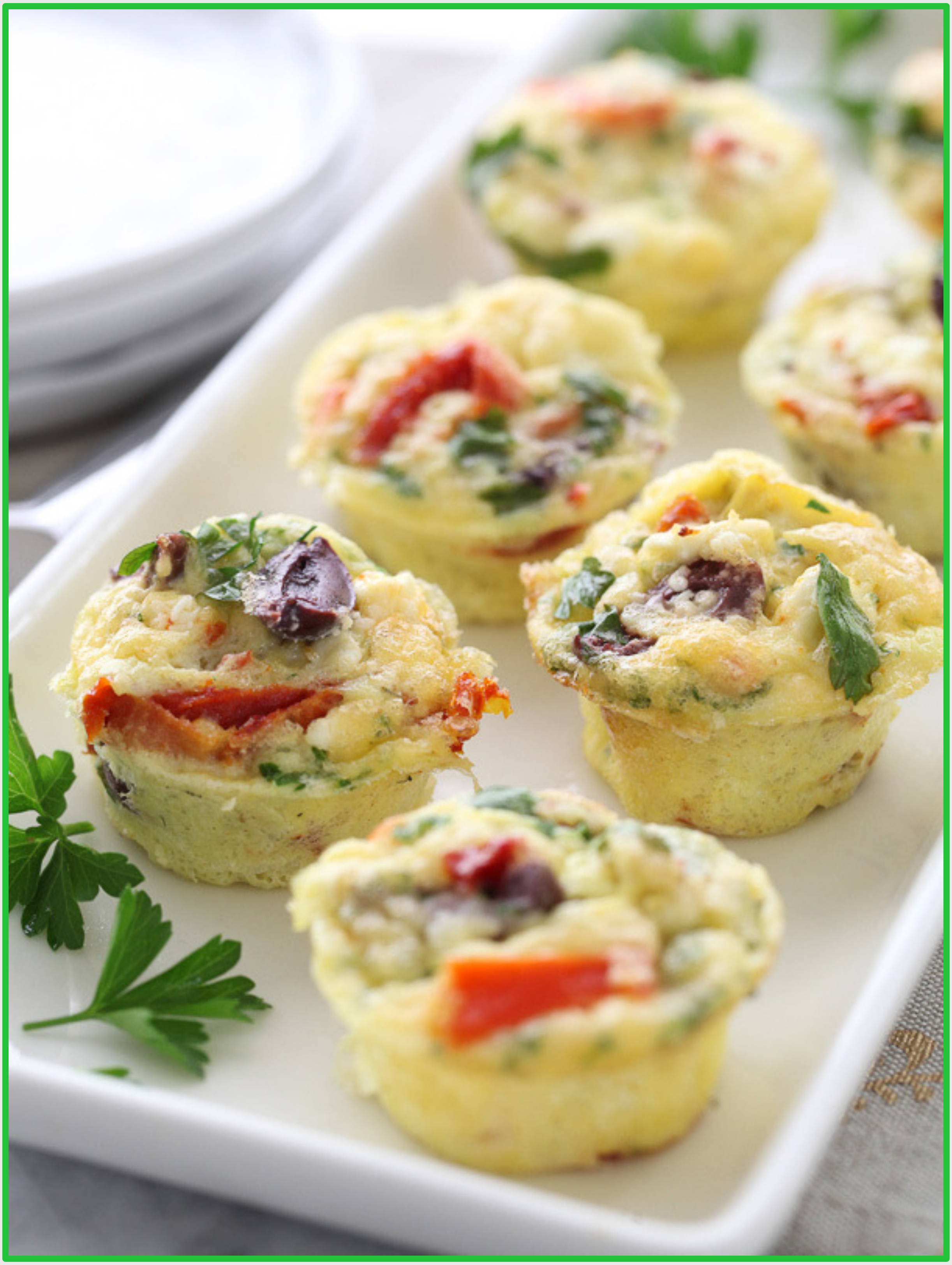

CHICKEN & VEG OMELETTE BITES

INGREDIENTS

- 6 large eggs
- 50ml semi-skimmed milk
- Salt and black pepper
- 1 tbsp. Extra virgin olive oil
- 2 medium spring onions (finely chopped)
- Half a red bell pepper (finely chopped)
- Olive oil (for greasing)
- 1 very large handful of spinach
- 2 tsp of grated parmesan cheese (optional)
- ½ tsp of dried mixed herbs
- 100g cooked chicken breast

MAKES 2 SERVINGS
35g of Protein/serving

INSTRUCTIONS

1. Preheat the oven to 190 degrees C.
2. Grease a bun tin (with 12 cups) lightly with a little olive oil. You can also use a small, roasting tray lined with baking parchment and cut the eggs later.
3. Add the olive oil to a large frying pan and over a medium heat, fry the chopped bell pepper and spring onion.
4. When the veg have softened, add the spinach and chicken and stir until soft.
5. Divide the vegetable mix evenly amongst the bun wells (or pour into the tray)
6. Put eggs, milk, salt, pepper, cheese and herbs in a separate bowl and mix well.
7. Pour the egg mixture evenly over the veg mix in the bun wells (or tray)
8. Bake until the egg is set, about 12-15 minutes. The eggs will deflate somewhat once removed from the oven and as they cool.
9. Run a knife around the edges of the omelette bites to loosen them from the bun tin or tray. Serve immediately or at room temperature.

PROTEIN SMOOTHIE

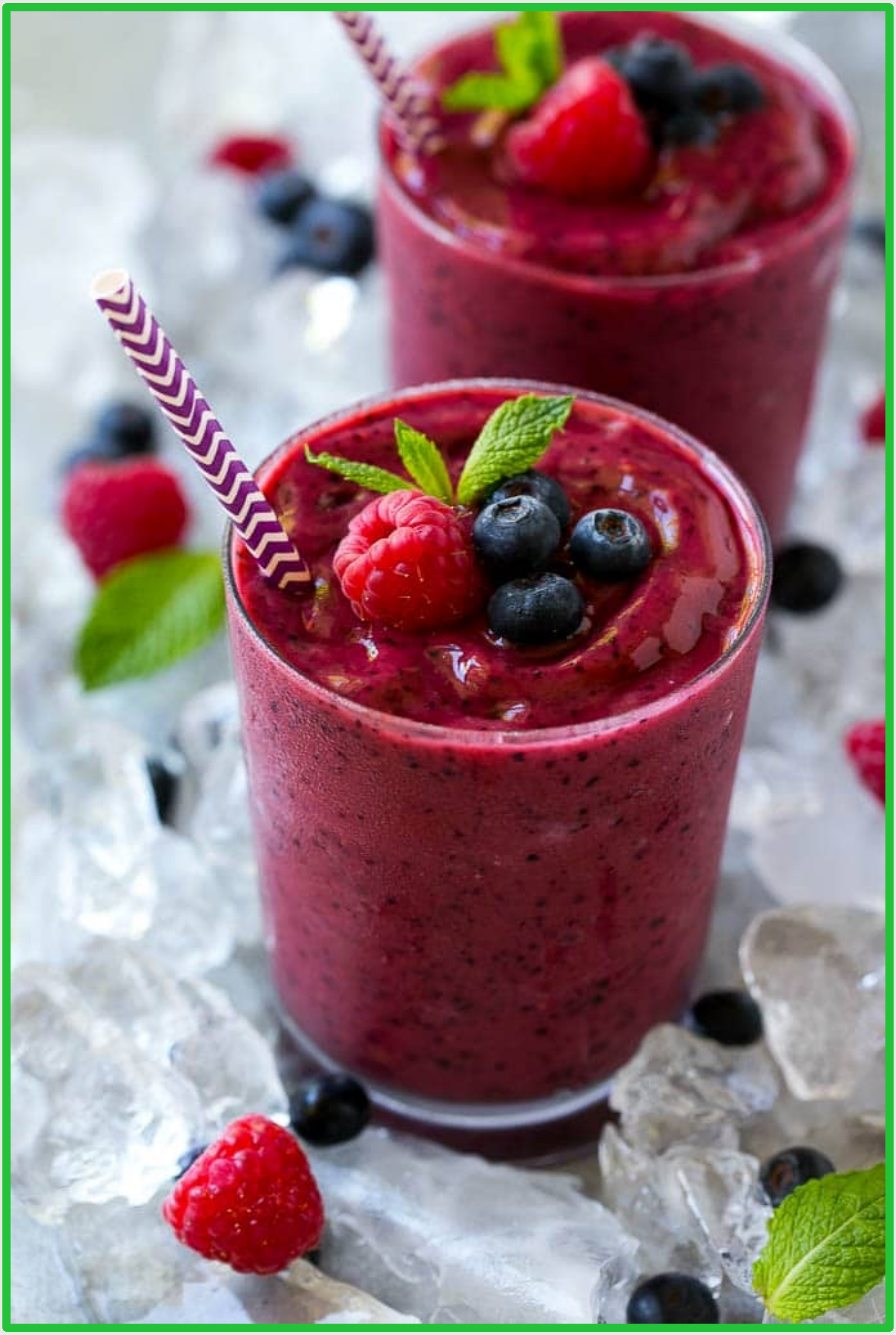

PROTEIN SMOOTHIE

INGREDIENTS

- 250g fat-free Greek yoghurt or quark
- 100g frozen blueberries or raspberries
- 1 banana
- 1 small carrot
- 1 handful of spinach leaves
- Granulated artificial sweetener to taste
- Water (if necessary to thin it out)

MAKES 1 SERVING

30g of Protein/serving

INSTRUCTIONS

1. Add all the ingredients (except the water and sweetener) to a strong blender and blend until smooth
2. Add the water and sweetener to the desired consistency and sweetness.

*Use any combination of fruits and vegetables that you like and feel free to add in spices like cinnamon or fresh herbs like mint.

MUSHROOM, SPINACH & CHEESE OMELETTE

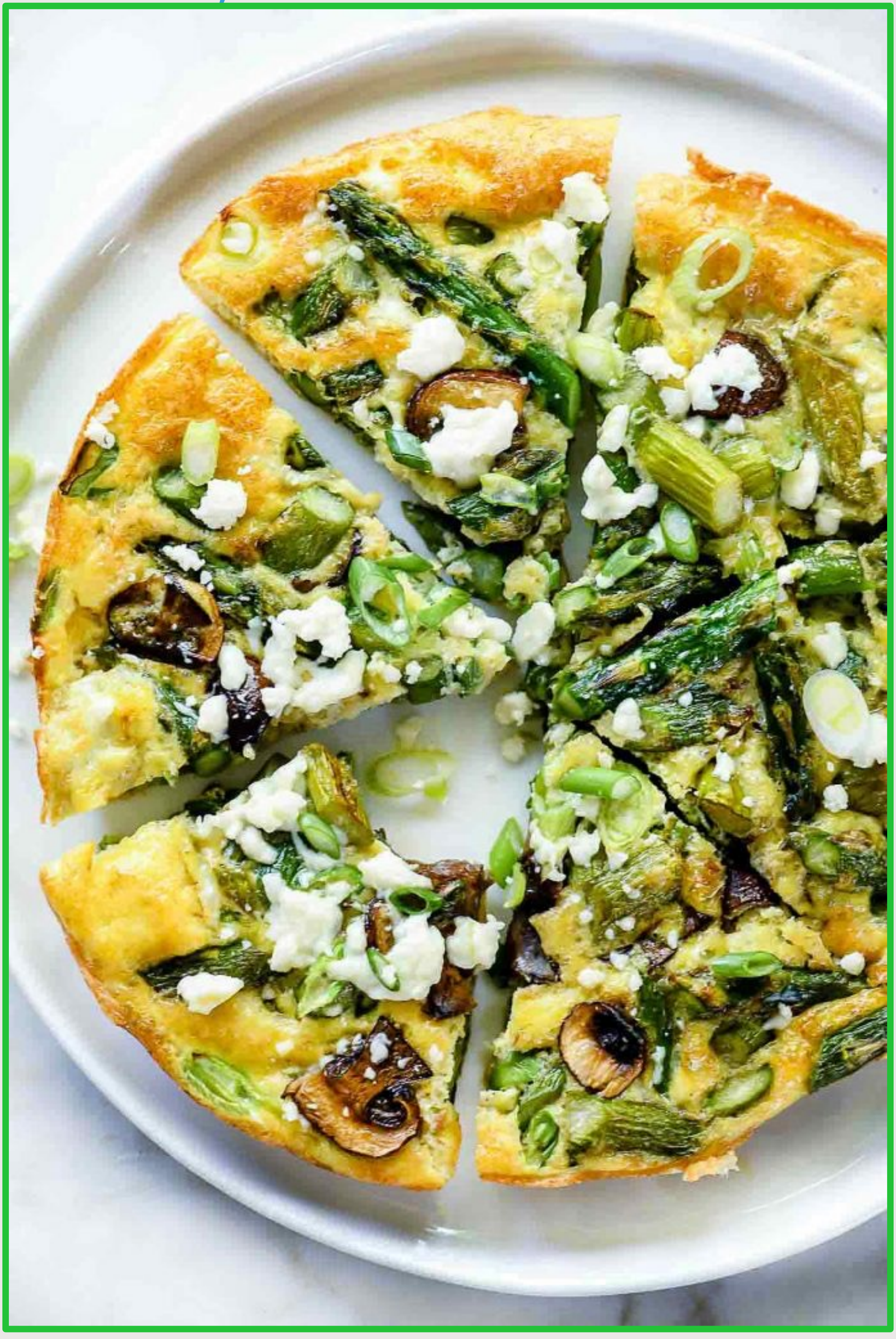

MUSHROOM, SPINACH & CHEESE OMELETTE

INGREDIENTS

- 3 large free range eggs
- Salt and pepper to taste
- 1 tbsp. extra virgin olive oil
- ½ small onion (finely chopped)
- 3-4 large murshrooms (sliced)
- 1 handful of spinach
- 35g of grated reduced-fat cheese
- ½ tsp of dried mixed herbs

MAKES 1 SERVING

33g of Protein/serving

INSTRUCTIONS

1. Heat the oil in a large frying pan and saute the onion and mushroom until softened.
2. Add the spinach and continue to cook until softened.
3. Beat the eggs in a bowl with the seasoning and grated cheese then pour over the mushroom and spinach mixture.
4. Cook the eggs undisturbed until the edges begin to pull away from the edge of the pan and begin to set. If you cover the frying pan with a lid, it will cook much faster.
5. If you have the skills you can flip the omelette or put the fry pan under a hot grill for 2-3 minutes or until the eggs have puffed and have cooked through.
6. Cut into wedges and serve warm or at room temperature

TINNED SARDINES ON TOAST

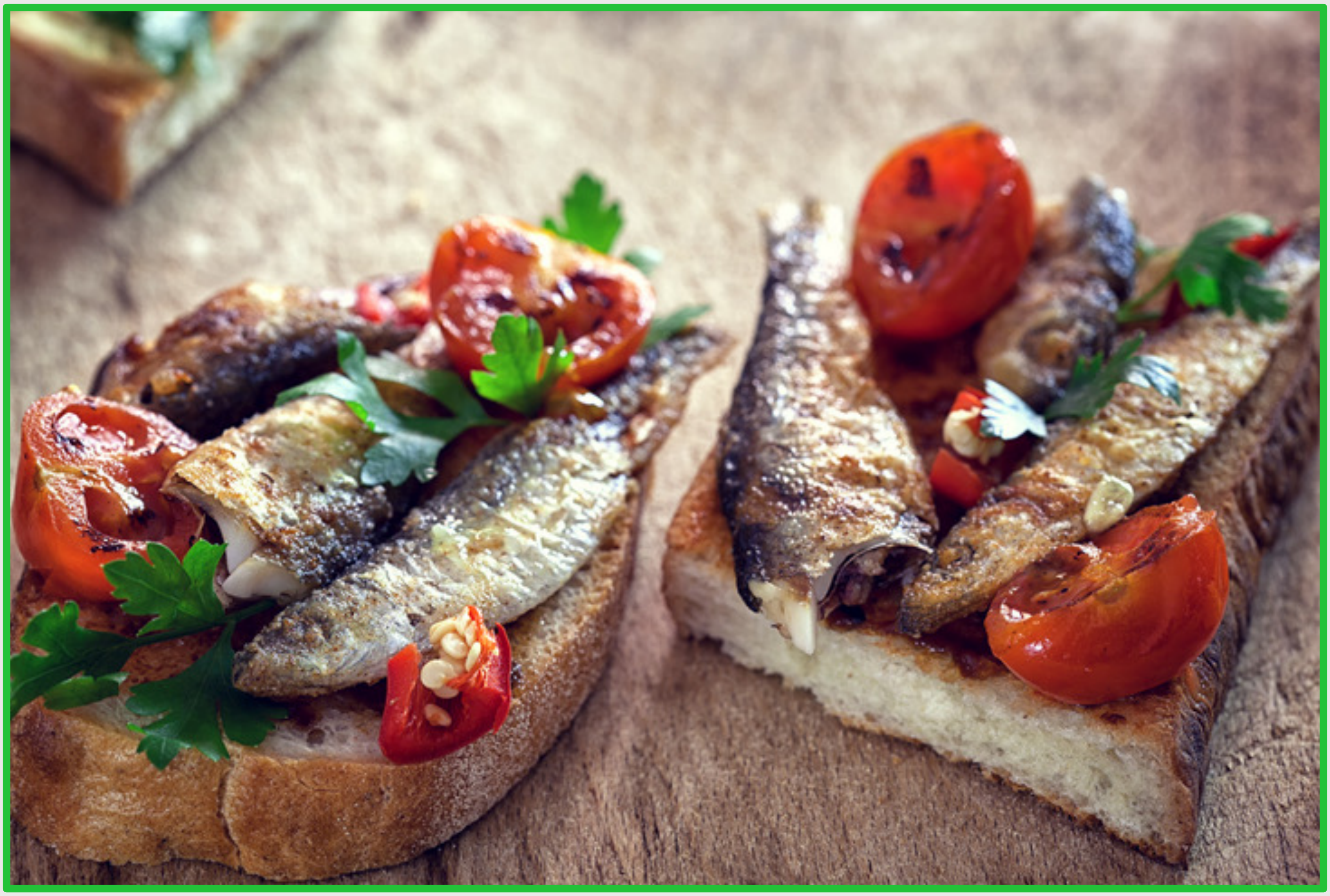

TINNED SARDINES ON TOAST

INGREDIENTS

- 2 tins of sardines in tomato sauce (120g each)
- 3 medium tomatoes or 5-6 cherry tomatoes (halved)
- 2 tbsp. extra virgin olive oil
- 2 slices of wholegrain bread
- Salt and black pepper

MAKES 2 SERVINGS
27g of Protein/serving

INSTRUCTIONS

1. Preheat the oven to 200 degrees C
2. In a bowl, mix the tomatoes, olive oil, salt, pepper and garlic powder.
3. Place on an oven tray and bake for 10 minutes until softened
4. 5 minutes before the tomatoes are done, place the sardines on a tray and into the oven to heat through
5. Toast the bread.
6. Place the bread onto a plate, top with the hot sardines and serve with the roasted tomatoes on the side

SALMON & BEAN SALAD

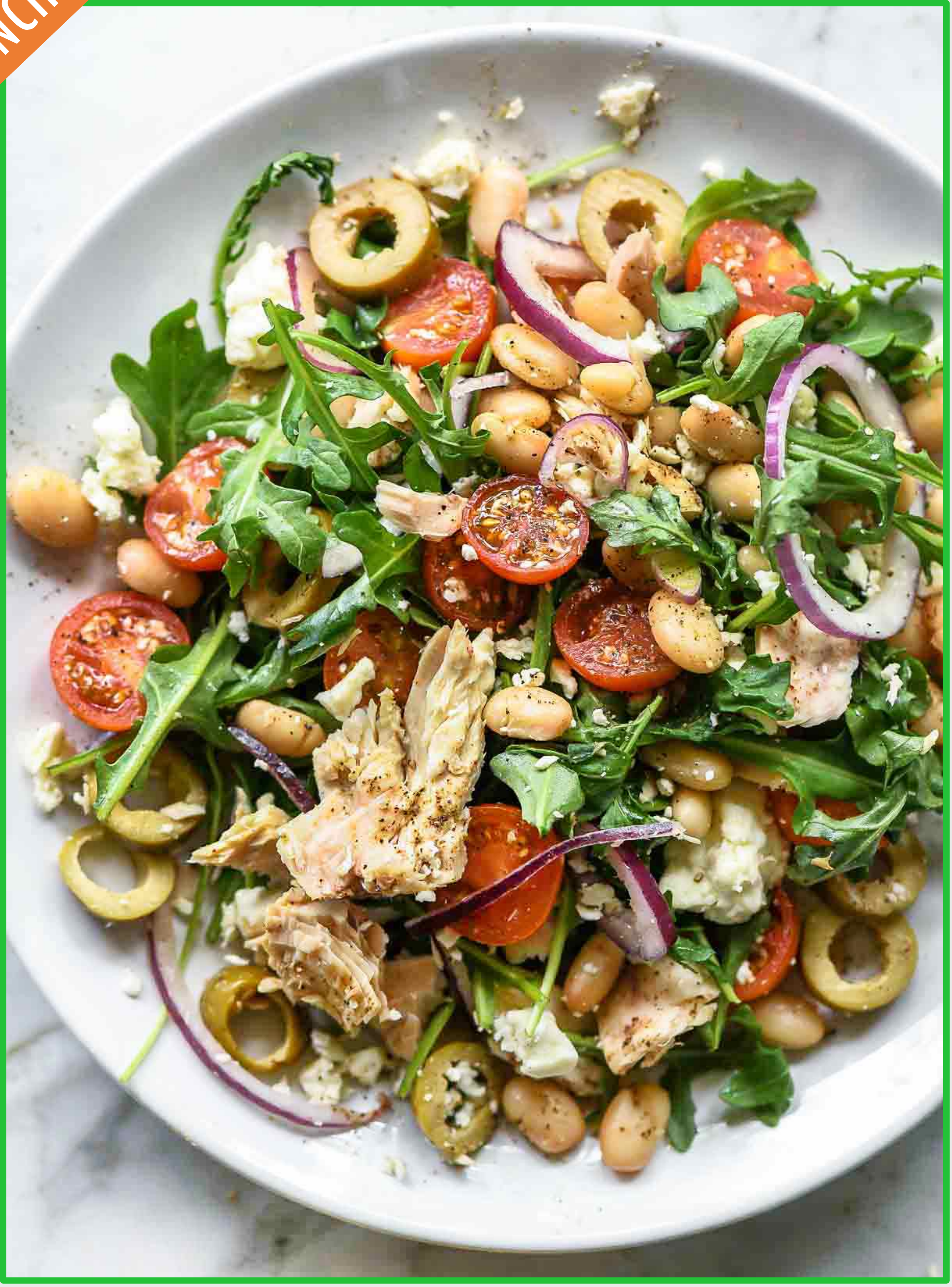

SALMON & BEAN SALAD

INGREDIENTS

- 2 handfuls of mixed leaves or favourite lettuce
- 1 400g can of cannellini/butter/mixed beans rinsed and drained
- 1 large can (213g) red or pink salmon in brine
- 1/2 cup cherry tomatoes halved
- 2 pickled gherkins (sliced)
- 1 small red onion (thinly sliced)
- 2 tablespoons extra virgin olive oil
- 1/2 lemon
- Salt and black pepper

MAKES 2 SERVINGS
29g of Protein/serving

INSTRUCTIONS

1. Slice the onions and place in a bowl of warm water to soak for 5 minutes (this reduces the sharp taste of raw onion)
2. In a large bowl, combine the leaves, beans, salmon, tomatoes, pickles and red onion. Drizzle with the olive oil and the juice from the lemon. Toss to combine.
3. Season to taste with salt and black pepper.

CHICKEN & FETA SALAD

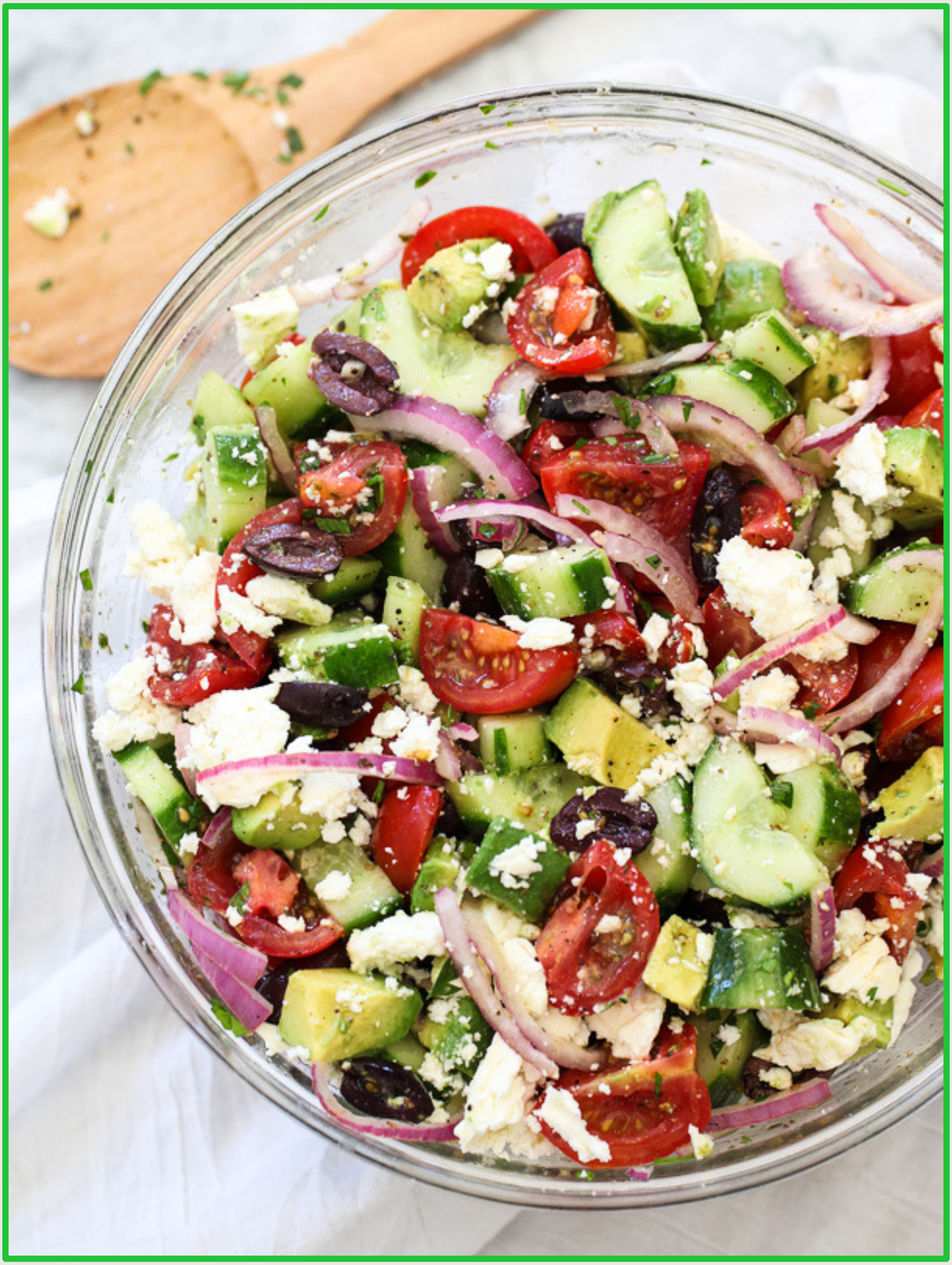

CHICKEN & FETA SALAD

INGREDIENTS

- 1/2 a cucumbers (peeled and cut into 1/2 inch slices)
- 100g small tomatoes (quartered)
- 1/2 red onion (thinly sliced)
- 2 tbsp. olives (or pickled gherkins) (sliced)
- 200g cooked chicken breast (chopped)
- 100g reduced-fat feta-style cheese (broken into large chunks)
- 2 tbsp. extra virgin olive oil
- 2 tbsp. vinegar
- 1 clove garlic (peeled and minced)
- 1 tablespoon dried oregano
- 2 teaspoons artificial sweetener
- Salt and black pepper to taste

MAKES 2 SERVINGS
30g of Protein/serving

INSTRUCTIONS

1. Slice the onions and place in a bowl of warm water to soak for 5 minutes (this reduces the sharp taste of raw onion)
2. In a large serving bowl, combine the cucumbers, tomatoes, red onion and olives.
3. In a cup or glass, combine the olive oil, red wine vinegar, garlic, oregano, sweetener and salt and pepper. Mix well until blended.
4. Pour ¾ of the dressing on the cucumber mixture and toss to coat.
5. Pour the remaining dressing on the chicken and feta and gently mix to coat.
6. Add the chicken and feta to the salad and serve.

BARBECUE BEEF & VEG WRAP

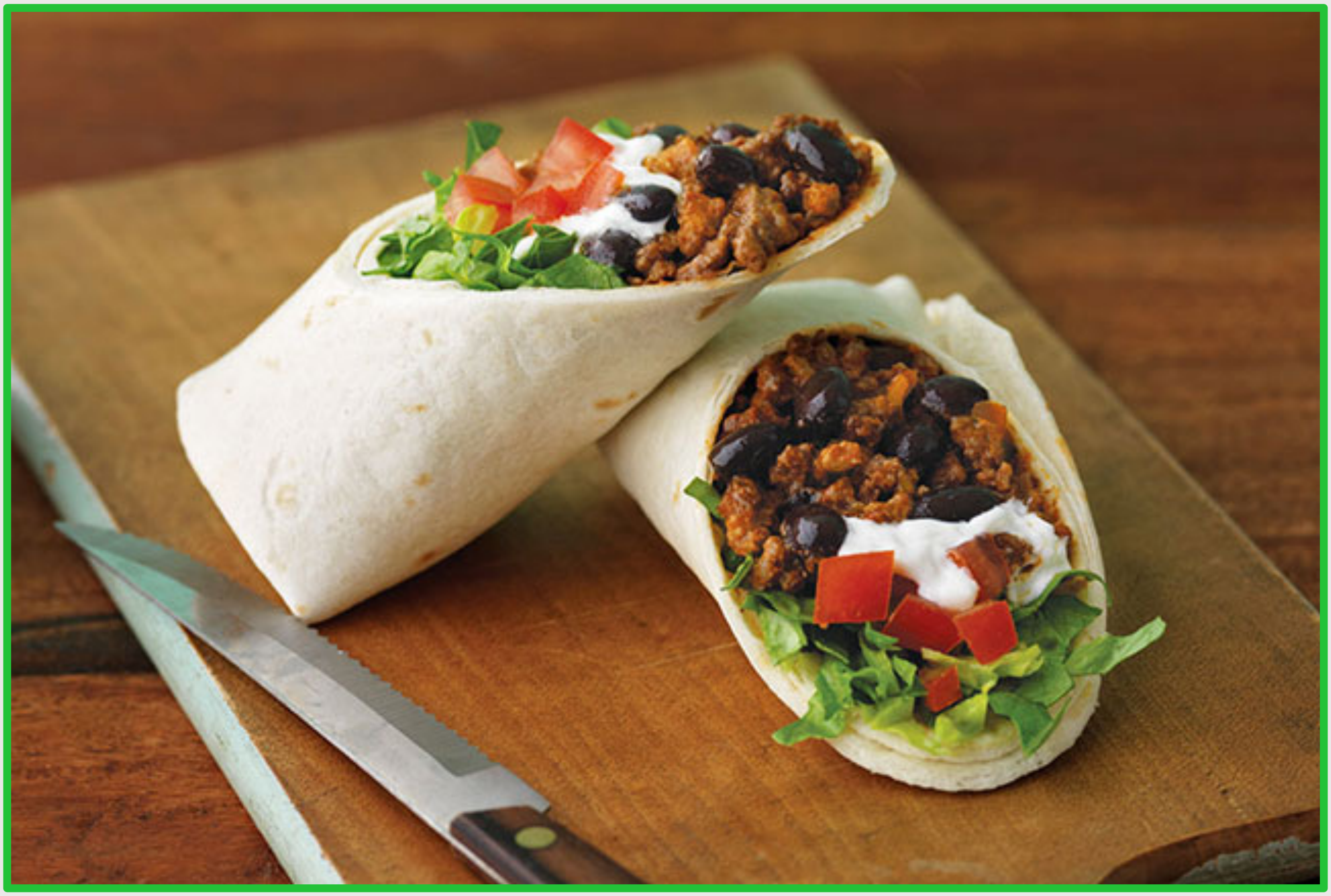

BARBECUE BEEF & VEG WRAP

INGREDIENTS

- 200g very lean beef mince
- ½ red bell pepper (finely chopped)
- ½ small onion (finely chopped)
- ½ can black beans (or favourite beans) (drained)
- Salt and black pepper
- 1 tsp dried mixed herbs
- 2 tbsp. extra virgin olive oil
- 3 tbsp. barbecue sauce
- 2 wholegrain wraps
- 1 handful of mixed salad leaves
- 2 tbsp. fat-free Greek yoghurt (optional)

MAKES 2 SERVINGS
32g of Protein/serving

INSTRUCTIONS

1. Fry the beef and chopped vegetables in the olive oil
2. When the beef is browned add the salt, pepper, mixed herbs and barbecue sauce.
3. Add half the beef mixture to each of the wraps and top each with half the salad leaves.
4. Drizzle over some of the Greek yoghurt (if using). Roll the wraps.
5. Serve immediately

EASY CHICKEN PITA

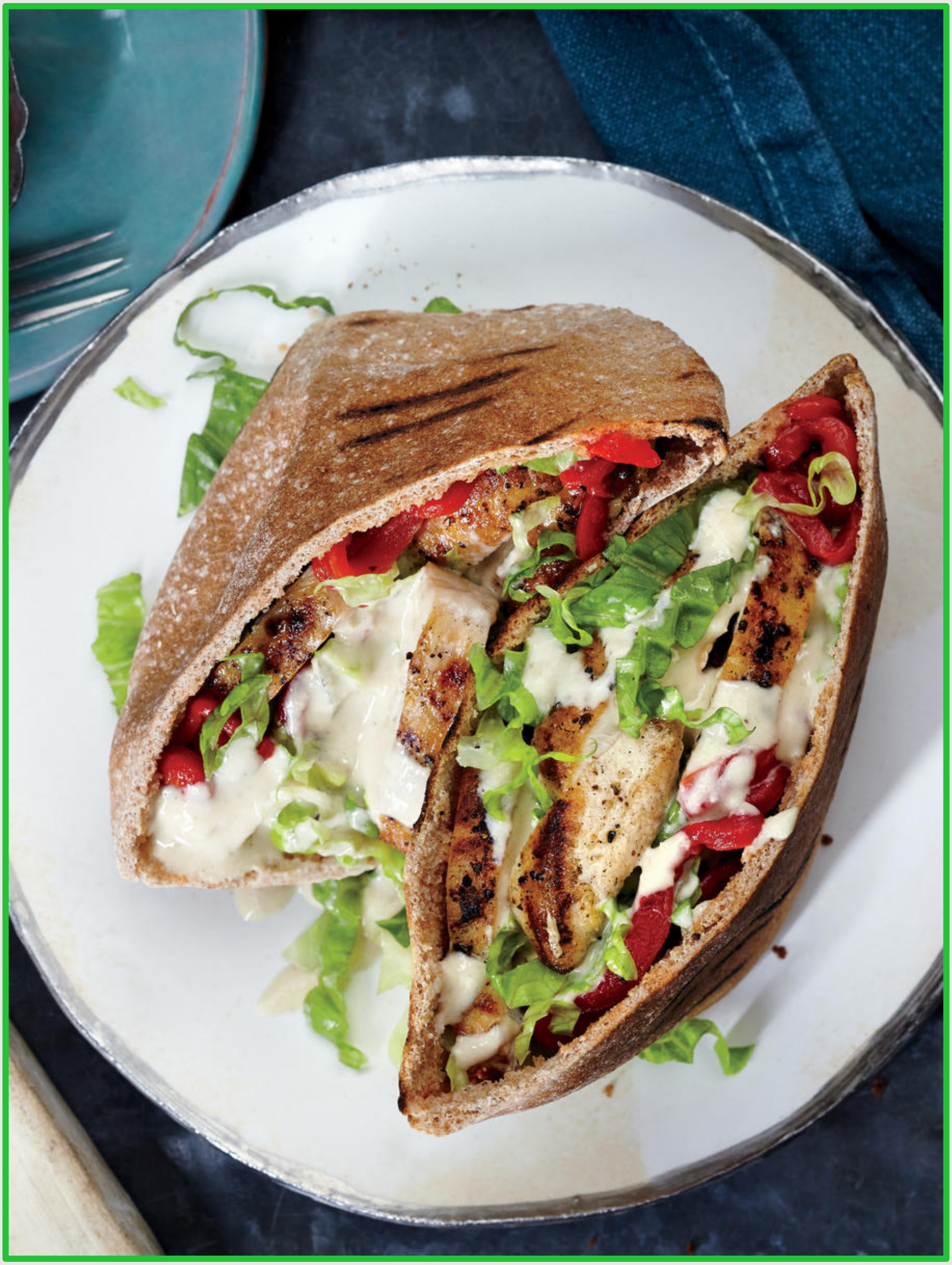

EASY CHICKEN PITA

INGREDIENTS

- 1 wholemeal pita bread
- 130g of raw chicken breast or 120g cooked chicken breast (chopped or pulled) if you buy pre-cooked chicken
- 1 tbsp. extra virgin olive oil
- Salt and black pepper
- Low-fat sauce of choice (ketchup, barbecue, sweet chilli etc.)
- Salad leaves of choice (spinach, lettuce, rocket etc.)
- Salad vegetables of choice (peppers, tomatoes, cucumber etc.) (chopped)

MAKES 1 SERVING

33g of Protein/serving

INSTRUCTIONS

1. If using raw chicken, cut into small chunks, sprinkle with salt and pepper and fry until cooked through
2. Rub a few drops of water over one side of the pita bread and place in the microwave for 20 seconds. This will make the pita puff up and be easier to open.
3. Slice open one side of the pita and fill evenly with leaves, vegetables and drizzle in the olive oil, salt and pepper.
4. Add the chicken and top with your sauces of choice. (You can also toss the chicken in your sauce of choice and microwave for 1 minute before adding it to the pita).
5. Serve immediately

SPINACH & BEAN MASH

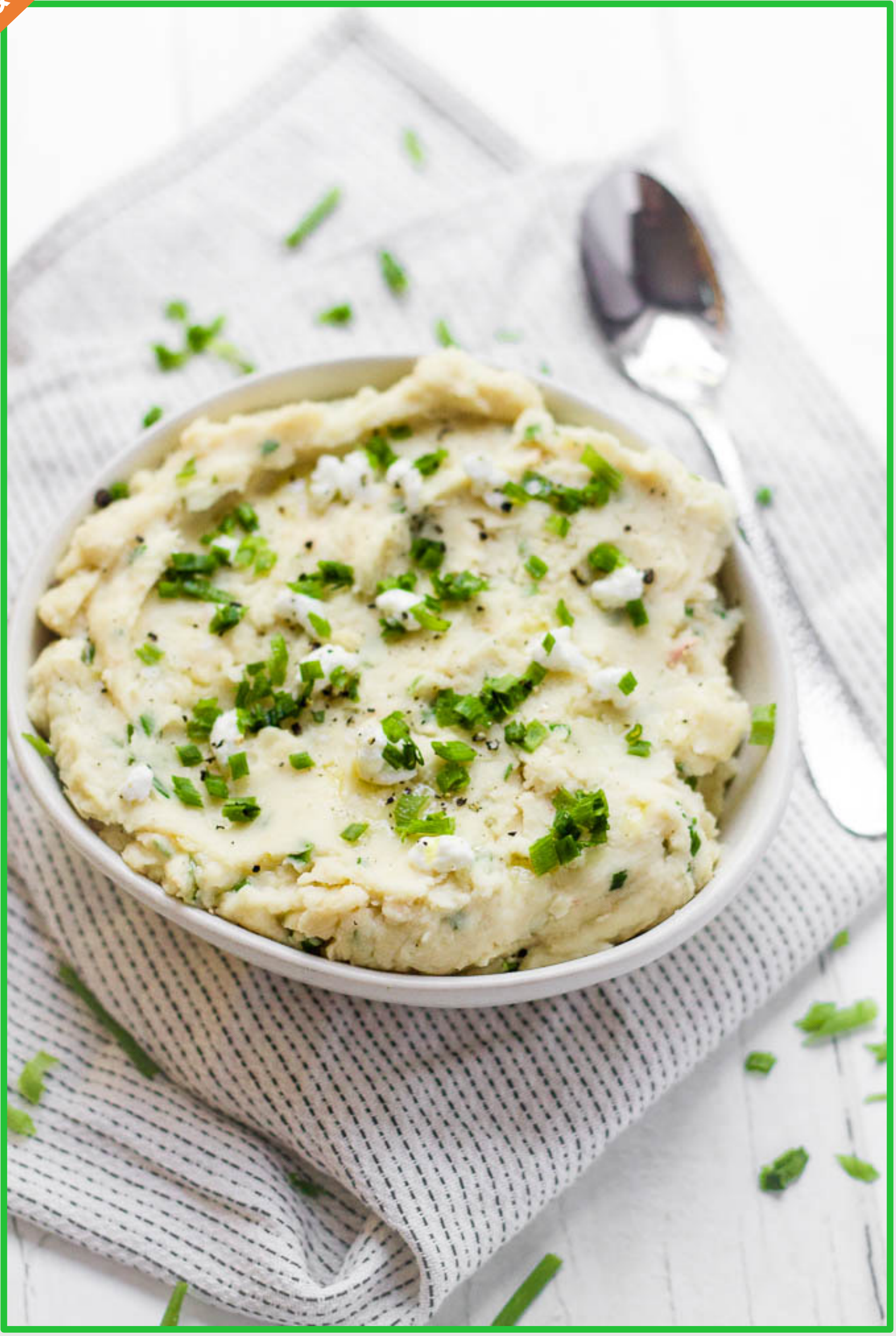

SPINACH & BEAN MASH

INGREDIENTS

- 2 tins of white beans (e.g. butter beans or cannellini) (rinsed and drained)
- 2 cloves of garlic (minced)
- 2 tbsp. olive oil
- ½ a vegetable stock cube dissolved in 100ml of hot water
- Salt and black pepper
- 2 handfuls of spinach (chopped0
- 1 spring onion (finely chopped)

MAKES 4 SERVINGS
8g of Protein/serving

INSTRUCTIONS

1. Heat the oil in a saucepan and cook the garlic and half the spring onion for 1 minute
2. Add the beans and half the stock, mix well and mash. Add more stock if necessary
3. Add the spinach, stir and allow to wilt.
4. Season with salt and pepper to taste.
5. Serve immediately topped with the remaining spring onion.

*This is a fantastic alternative to mashed potato and can be served with many other main dishes

HERBY ROAST POTATOES

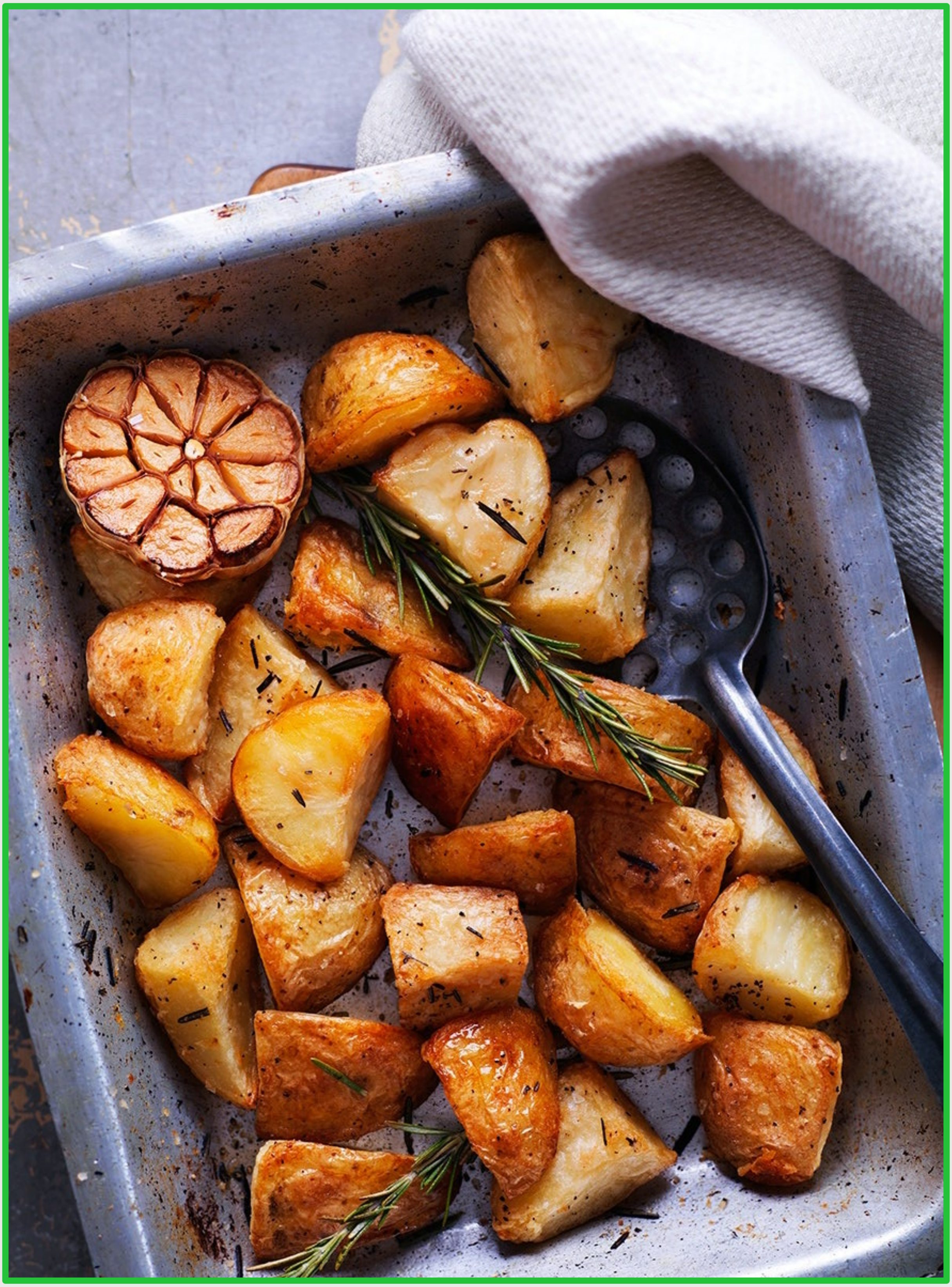

HERBY ROAST POTATOES

INGREDIENTS

- 4 medium potatoes (cut into quarters)
- 2 tbsp. extra virgin olive oil
- Salt and black pepper
- Mixed herbs & garlic powder (optional)

MAKES 2 SERVINGS
0g of Protein/serving

INSTRUCTIONS

1. Preheat the oven to 200 degrees C.
2. Pierce the potato quarters with a fork and place in a bowl and microwave on high for 10 minutes (or until starting to soften).
3. Once slightly softened, add the olive oil, salt, pepper and herbs & garlic if using and toss to cover.
4. Place on a baking tray, skin side down and roast in the oven for 20 minutes or until golden and crispy on the outside.
5. Serve immediately

*You can also make this recipe with sweet potato

SIMPLE PEAS & ONIONS

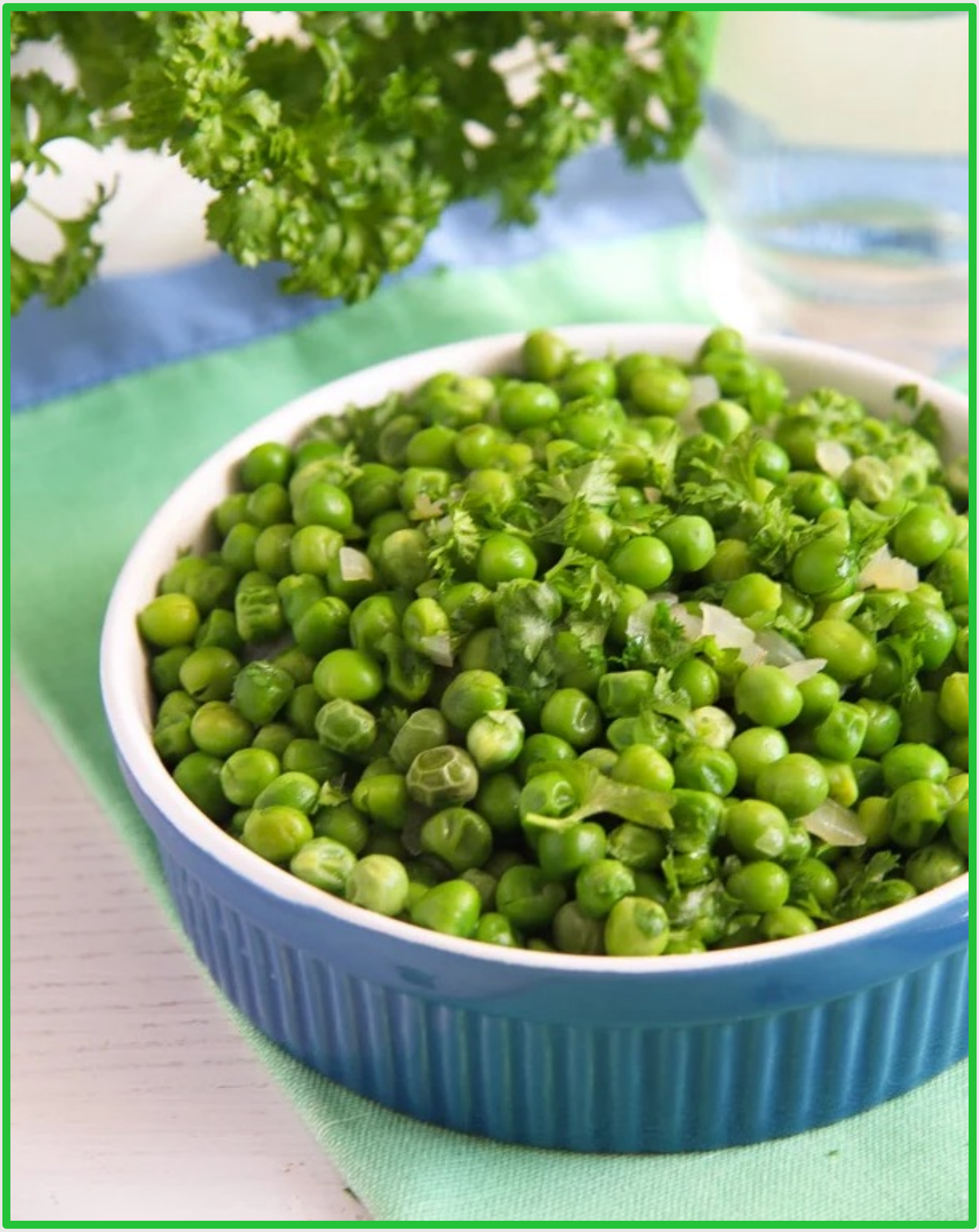

SIMPLE PEAS & ONIONS

INGREDIENTS

- 1 small onion
- 1 tablespoon olive oil
- 450 g frozen peas
- 60ml water
- Salt and black pepper
- Small bunch of parsley (optional)

MAKES 4 SERVINGS
7g of Protein/serving

INSTRUCTIONS

1. Chop the onion very finely. Heat the oil in a saucepan and cook the onion until translucent and softer, about 3 minutes.
2. Add the peas, water and a little salt, stir and bring to a boil. Cover the saucepan, leaving a small crack open, turn down the heat and simmer the peas for about 3-4 minutes or until cooked to your liking. Do not overcook.
3. Check the salt, add some freshly ground black pepper and sprinkle with the chopped parsley. Serve immediately.

ROAST POTATO WEDGES

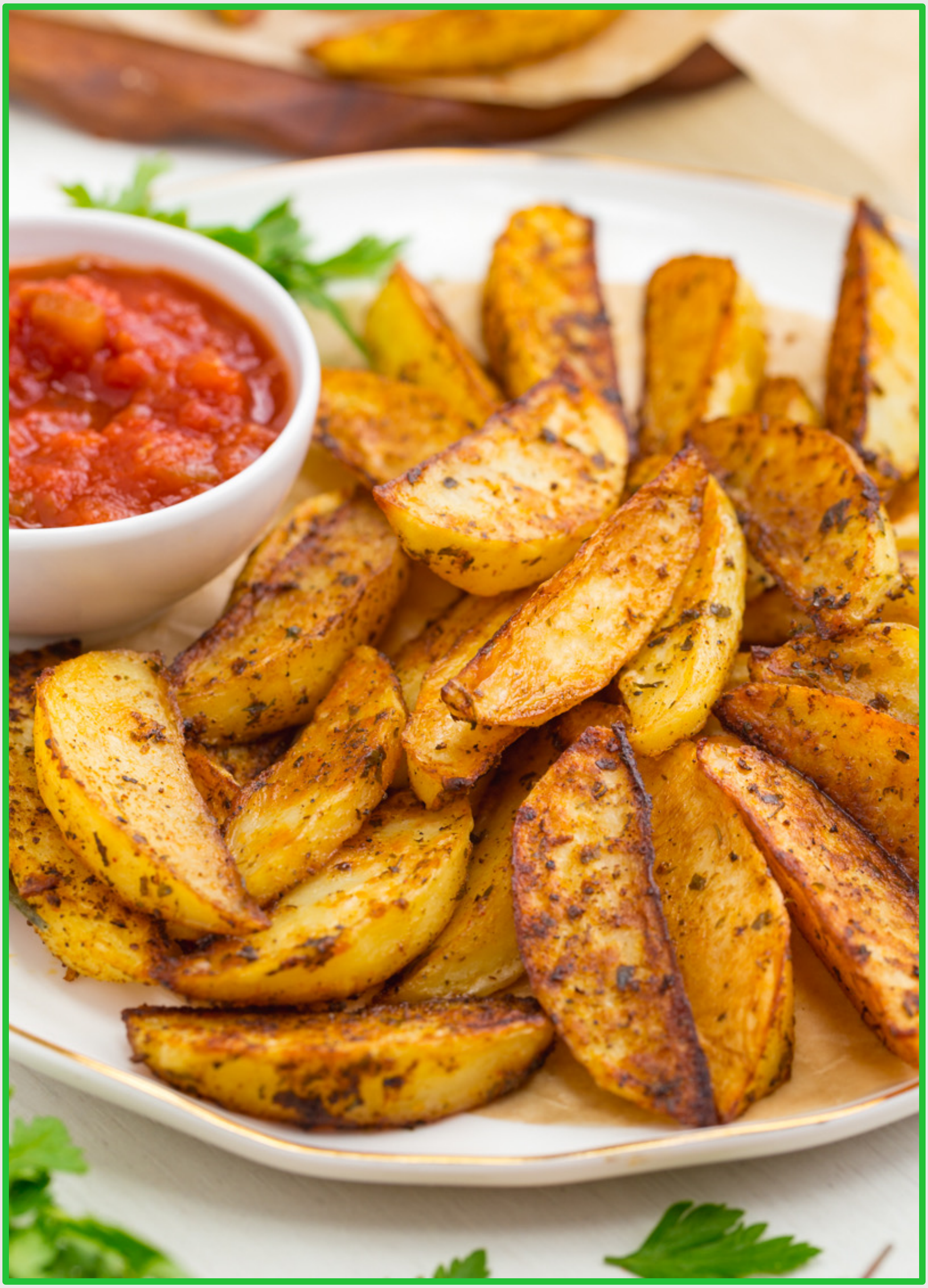

ROAST POTATO WEDGES

INGREDIENTS

- 4 medium potatoes (cut into wedges)
- 2 tbsp. extra virgin olive oil
- Salt and black pepper
- Mixed herbs & spices of choice (a tasty mix can include garlic powder, cumin, oregano, Italian seasoning, onion powder, chili powder or even curry powder)

MAKES 2 SERVINGS
0g of Protein/serving

INSTRUCTIONS

1. Preheat the oven to 200 degrees C.
2. Put the potato wedges in a bowl add the olive oil and mix to coat the wedges.
3. Add the salt, pepper and herbs & spices and toss to cover.
4. Place on a baking tray, skin side down and roast in the oven for 25 minutes or until golden and crispy on the outside.
5. Serve immediately

*You can also make this recipe with sweet potato

QUICK FROZEN VEG STIR-FRY

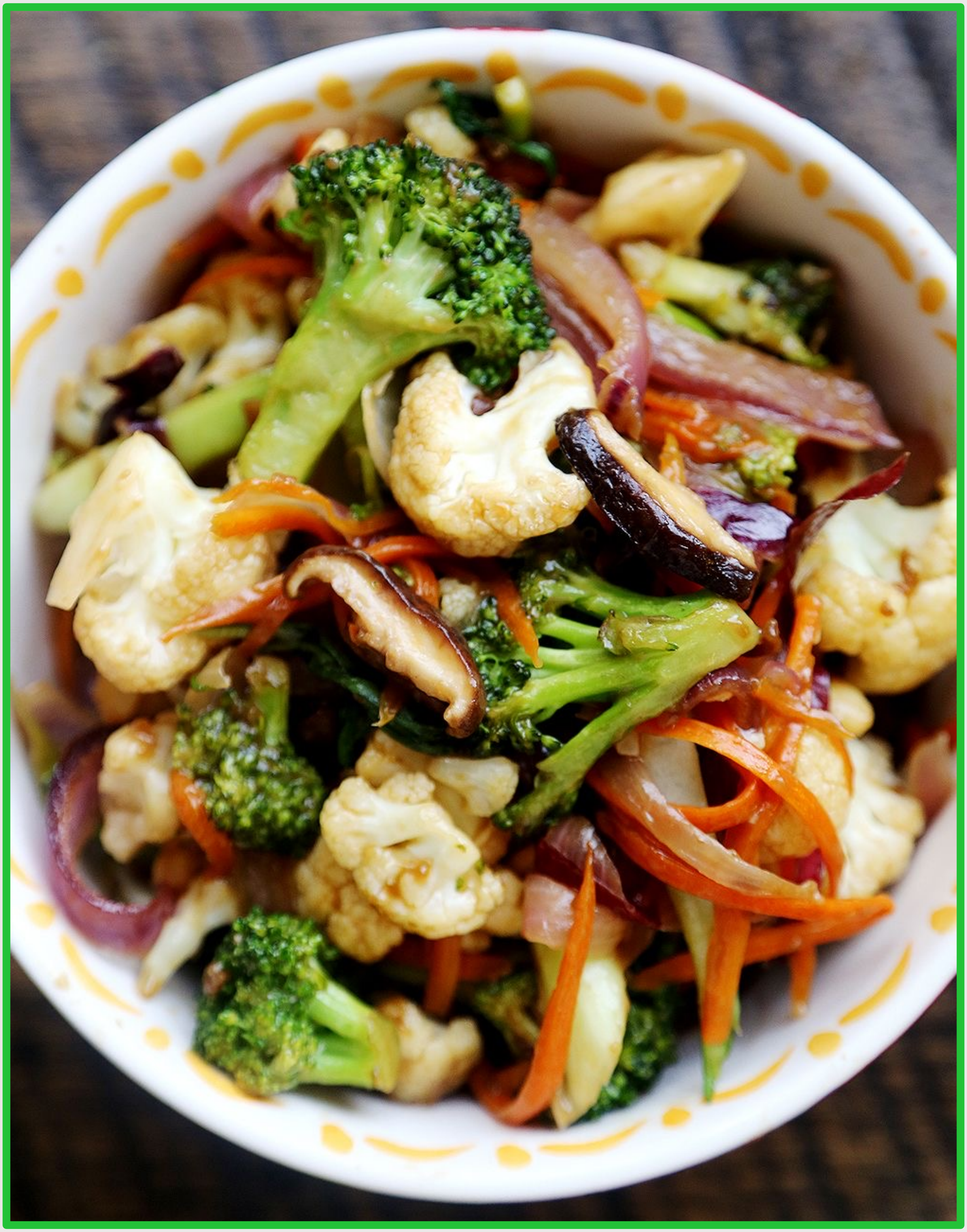

QUICK FROZEN VEG STIR-FRY

INGREDIENTS

- 200g frozen veg (broccoli, cauliflower, carrots, peas etc.)
- 1 tablespoon olive oil
- Black pepper
- 1 tbsp. Soya sauce
- Garlic powder

MAKES 1 SERVING

0g of Protein/serving

INSTRUCTIONS

1. Put the veg into a microwave-proof bowl and microwave for about 4 minutes or until just tender.
2. Heat the olive oil in a frying pan over a medium heat, add the veg and toss well in the olive oil for 1-2 minutes.
3. Add the soya sauce, pepper and garlic powder and stir to coat the veg.
4. Continue stirring for 1 minute and serve immediately.

*This works fine with any veg and fresh veg is perfect too.

ROAST VEGETABLE AND BEAN BAKE

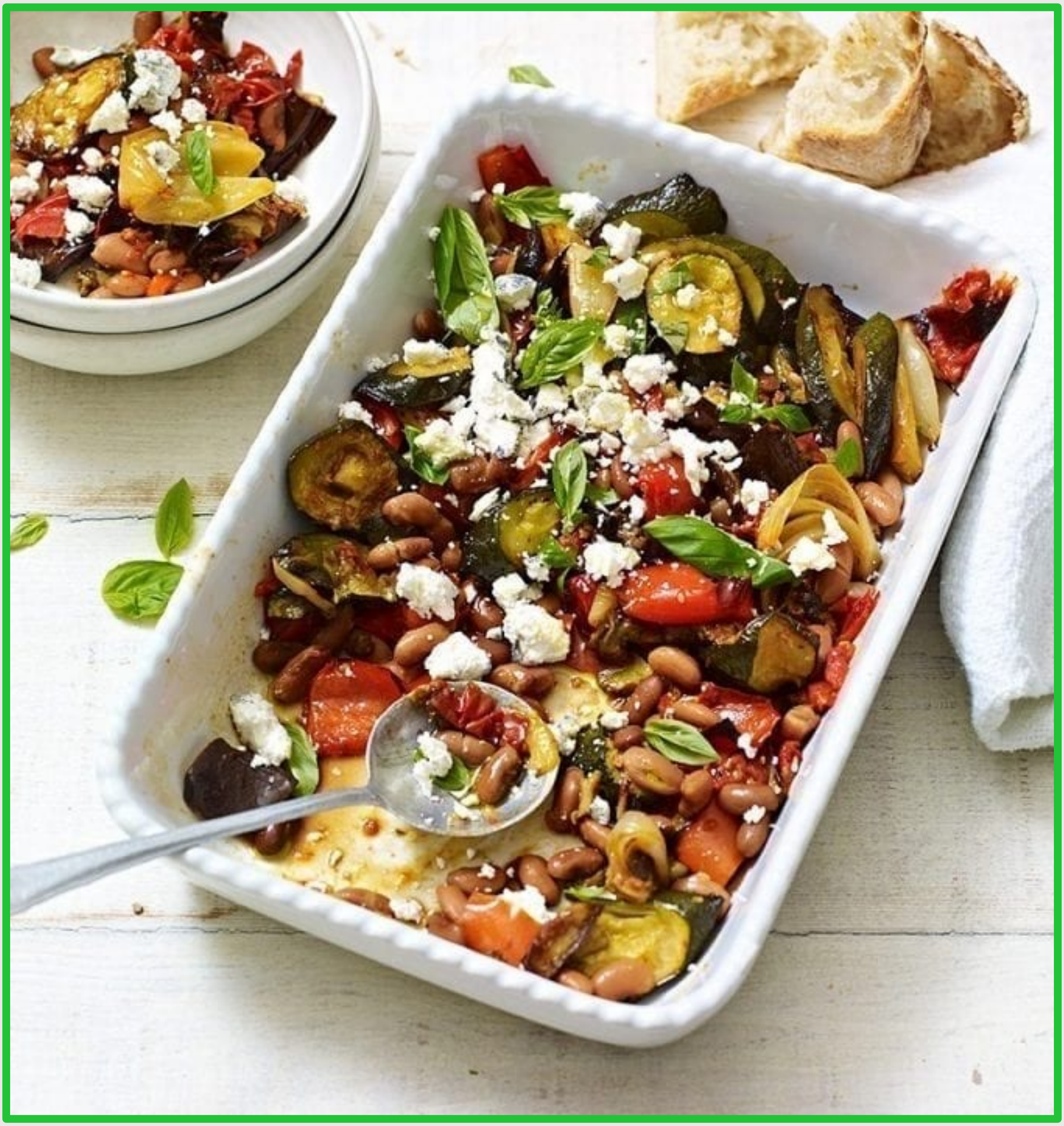

ROASTED VEGETABLE AND BEAN BAKE

INGREDIENTS

- 2 garlic cloves, finely chopped
- 2 aubergines, cut into 2cm pieces
- 500g courgettes, cut into 1cm slices
- 2 large onions chopped into 2cm chunks
- 3 red peppers, cut into 2cm pieces
- 400g cherry tomatoes
- 5 tbsp olive oil
- 2 x 400g tins beans (cannellini, chickpeas, black etc.) drained and rinsed
- 100g reduced fat (50%) cheddar-style cheese, grated
- 1 tbsp balsamic vinegar (optional)
- Handful of basil leaves to serve (optional)

MAKES 4 SERVINGS

16g of Protein/serving

INSTRUCTIONS

1. Heat the oven to 200° C/180° C fan. Put the onion, garlic, aubergines, courgettes, red peppers and tomatoes into 2 large roasting dishes. Drizzle over 4 tbsp oil, season with salt and pepper, then toss to coat. Roast for 40 minutes.
2. Stir the cannellini beans into the roasted veg, sprinkle over the grated cheese then roast for 5 minutes more.
3. Drizzle over the remaining 1 tbsp olive oil and the balsamic vinegar at the table. Sprinkle with the fresh basil leaves, then serve.

HEALTHY COLESLAW

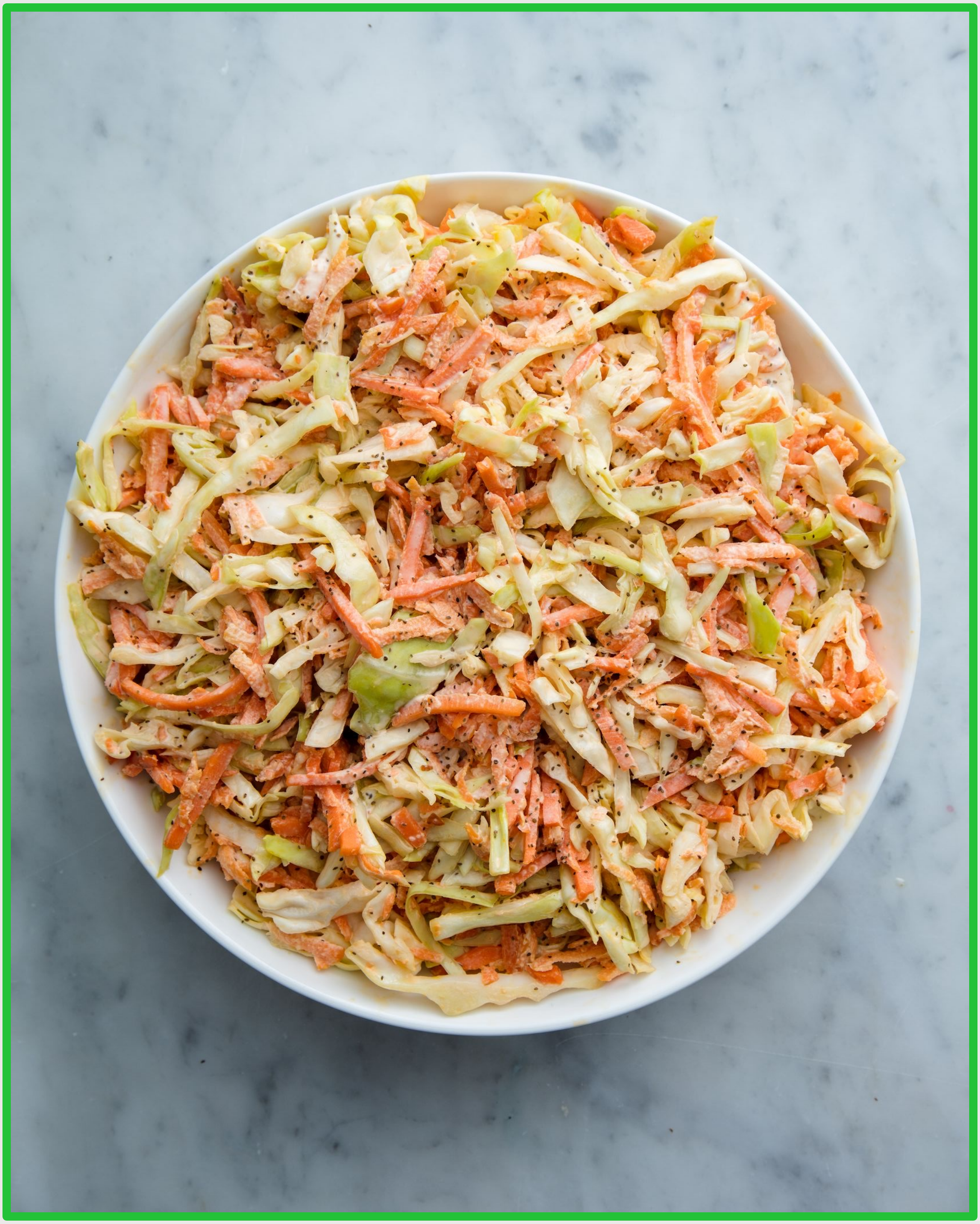

HEALTHY COLESLAW

INGREDIENTS

- ½ savoy or white or red cabage (or a mix of all 3), cored and shredded
- 1 apple, cored and grated (optional)
- 2 carrots, peeled and grated
- ½ onion, peeled and finely sliced
- 100g fat-free Greek yoghurt
- Juice of half a lemon
- 2 tsp vinegar
- 2 tsp mustard of choice (wholegrain, Dijon etc) (optional)

MAKES 6 SERVINGS
1g of Protein/serving

INSTRUCTIONS

1. Mix the cabbage, apple, carrots and onion in a large bowl.
2. In a separate bowl, mix the yogurt, lemon juice, vinegar and mustard. Season, then pour over the vegetables.
3. Give everything a good stir to coat in the dressing and eat immediately, or chill until you are ready to serve.
4. Can be stored in the fridge in a well sealed container

*This sauce is delicious as a side salad, in sandwiches or on burgers

TOMATO SALSA

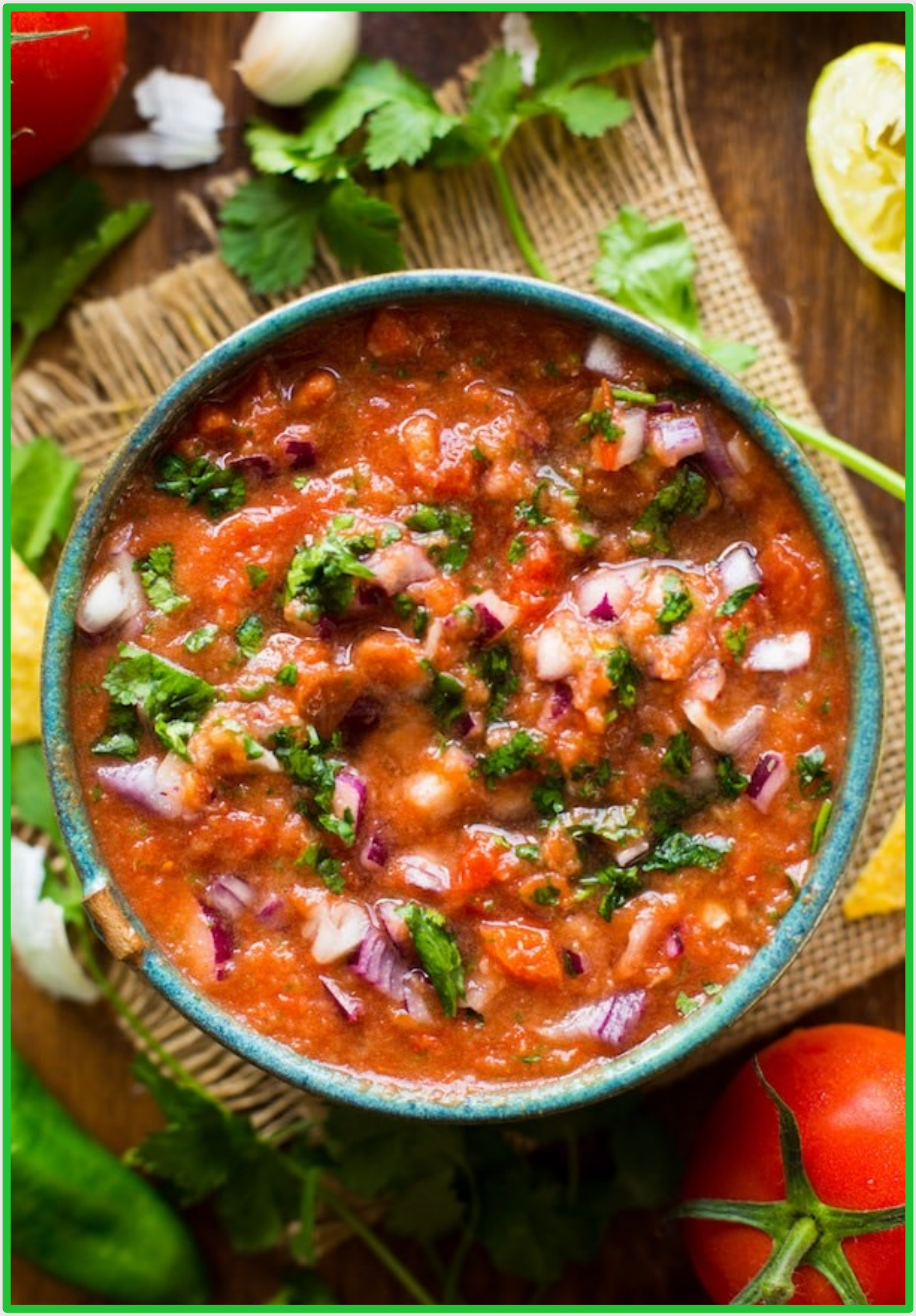

TOMATO SALSA

INGREDIENTS

- 3 medium sized, very ripe tomatoes
- 1 chilli pepper, seeds removed (optional)
- 1/4 medium onion (red or white), finely chopped
- Juice of half a lime
- 1 tbsp. fresh corriander roughly chopped (optional)
- Salt or to taste

MAKES 6 SERVINGS
0g of Protein/serving

INSTRUCTIONS

1. Chop the onion finely and place in a small bowl, covered with water (to help mellow the flavour)
2. Cut the tomatoes into quarters and remove the seeds. You can scoop out the seeds or cut the insides out with a knife.
3. Chop the tomatoes very finely
4. Drain off the water from the onions and add to the tomatoes in a bowl
5. Add the remaining ingredients, mix well and season to taste.

*This salsa is a great dip for thinly sliced vegetables or can be added to sandwiches, wraps and burgers

TZATZIKI SAUCE

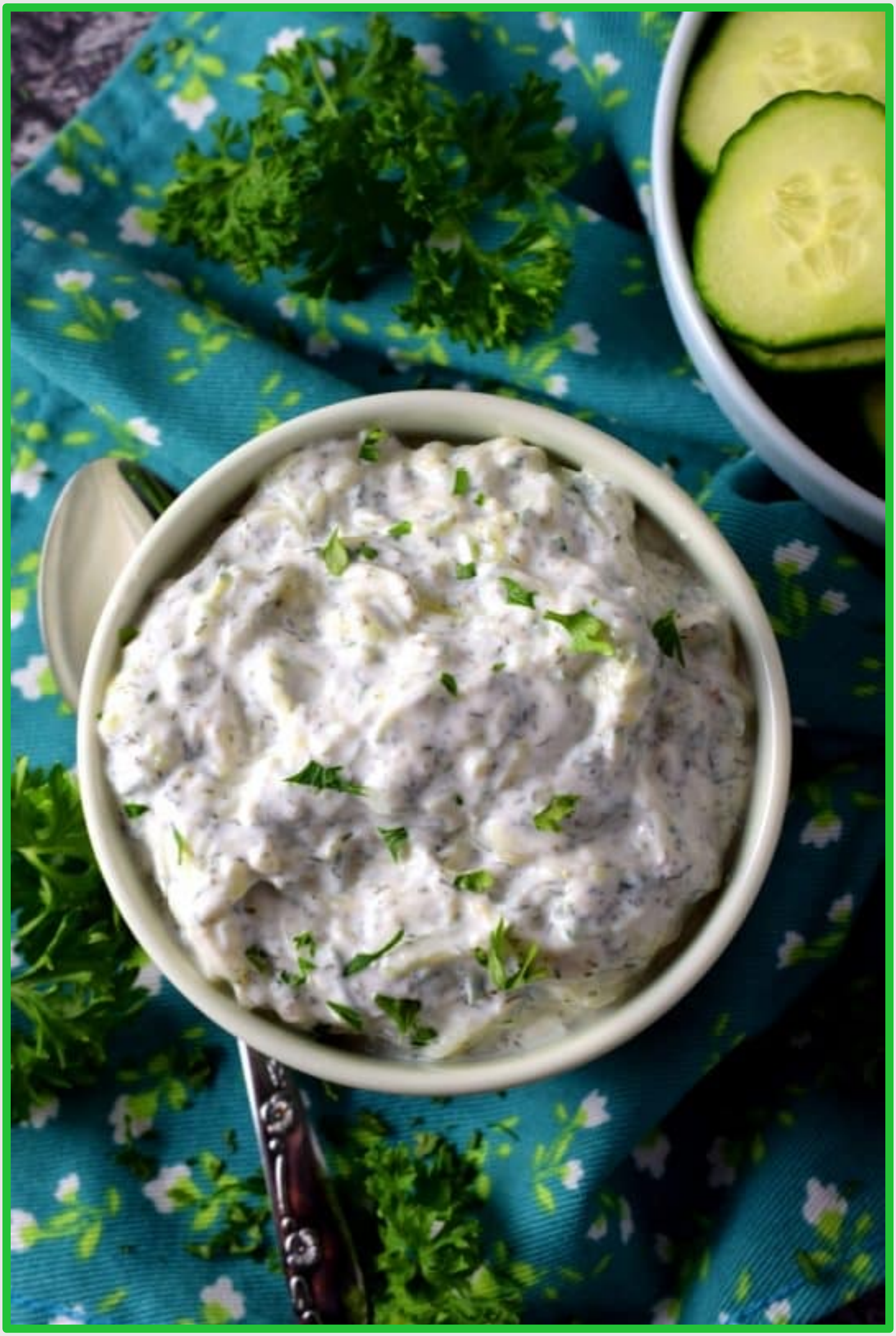

TZATZIKI SAUCE

INGREDIENTS

- 250g Fat-free Greek yogurt
- 1 large cucumber, seeded and finely grated*
- 2 cloves garlic, minced
- 3 teaspoons dried mint (optional)
- 1 tablespoon lemon juice
- 1/2 teaspoon salt
- 1/2 teaspoon ground black pepper

MAKES 10 SERVINGS

2g of Protein/serving

INSTRUCTIONS

1. Once you have shredded the cucumber, squeeze as much of the liquid out of the cucumber as you possibly can. Otherwise, your sauce will be too watery.
2. Add all of the prepared ingredients to a bowl and mix together. Transfer to a container with a tight fitting lid.
3. Refrigerate for one to two hours for best results.

*If the sauce becomes watery after refrigerating, in spite of the squeezed cucumber, simple whisk the sauce again to incorporate the water that may have settled on top of the sauce.

This sauce is delicious on salads, in sandwiches or as a dip for vegetables

PRAWNS & VEGGIE SPAGHETTI

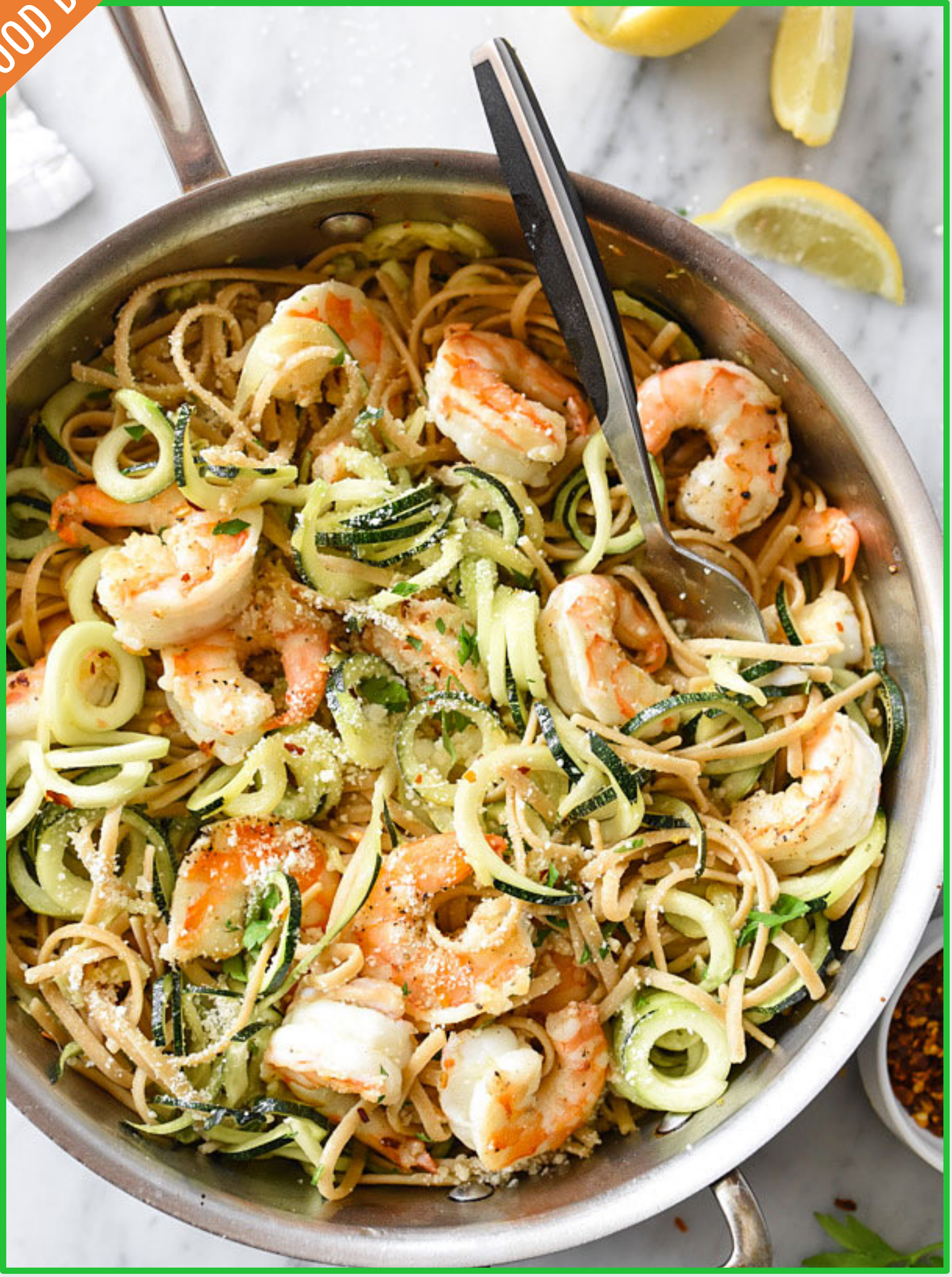

PRAWNS & WHOLEWHEAT SPAGHETTI

INGREDIENTS

- 300g raw frozen prawns (defrosted in fridge)
- 3 tbsp. extra virgin olive oil
- 2 large cloves garlic (pressed or minced)
- 2 handfuls of courgette noodles (or other vegetable noodle)
- Salt and black pepper
- 50g Wholewheat spaghetti
- 2 tbsps. Lemon juice
- 1 tsp. red chili flakes optional)
- 1 tbsp. grated Parmesan cheese
- Chopped parsley (optional)

MAKES 2 SERVINGS

26g of Protein/serving

INSTRUCTIONS

1. Add the prawns to a medium size bowl. Drizzle with 1 tablespoon of olive oil, add 1 clove of the minced garlic, salt and black pepper, toss and set aside.
2. Bring a large pot of salted water to a boil. Cook the spaghetti according to package directions and add the courgette noodles for the last minute. Use tongs to transfer the cooked spaghetti to a strainer and reserve the pasta water, brining it to a slow bubbling simmer.
3. In a large, high-sided frying pan, add 1 tablespoon of olive oil to the pan over medium heat. Place the shrimp in the pan and cook for 2 minutes on each side or just until opaque. Transfer the shrimp to a plate.
4. In the same pan, add 2 tbsp. of olive oil add the remaining minced clove of garlic, lemon juice, red chili flakes and 2 tbsp. reserved pasta water. Cook for 1 minute stirring once or twice.
5. Add the noodles, shrimp and 1/2 the Parmesan cheese and toss to coat.
6. Top with the remaining Parmesan cheese and parsley and serve.

HEALTHY FISH & CHIPS WITH MUSHY PEAS

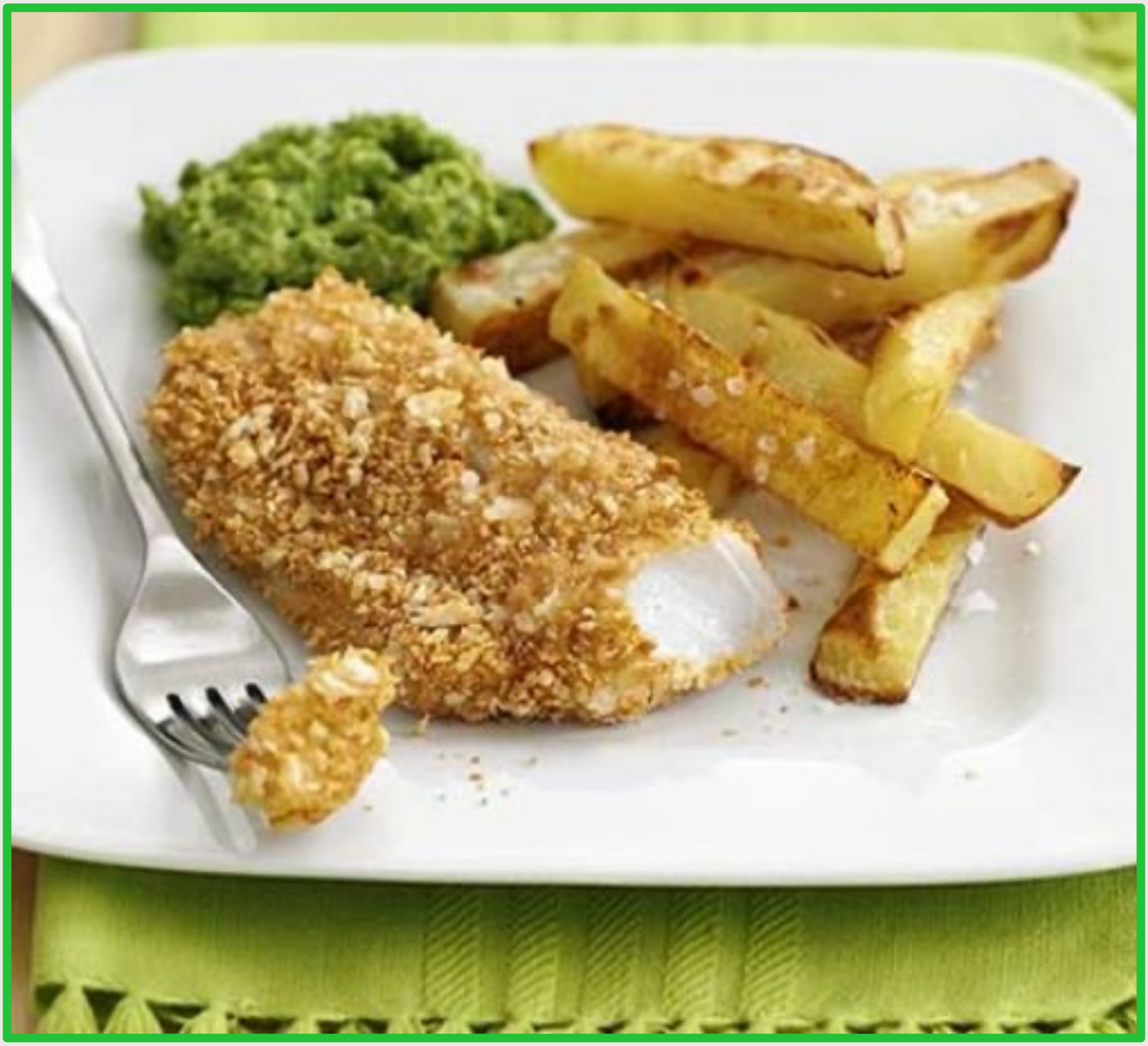

HEALTHY FISH & CHIPS WITH MUSHY PEAS

INGREDIENTS

- Potato wedges (page 27) (made without the spices)
- Simple peas & onions (page 25), mashed (half the recipe)
- salt and pepper
- 1 heaped tbsp. of paprika
- 50g flour
- 1 egg
- 2 slices of slightly stale toast, grated into breadcrumbs
- 1 tbsp. herbs (fresh/dry parsley or dill), finely chopped
- 1 lemon, zest and juice
- Pinch of pepper
- 2 white fish fillets (150g each)

MAKES 2 SERVINGS

35g of Protein/serving

INSTRUCTIONS

1. Preheat the oven to 190°C
2. Prepare the potatoes as per the recipe on page 27 (don't use the spices)
3. Lightly oil another baking tray/sheet with olive oil and place in the oven
4. While the potatoes are roasting, tip the flour into a bowl. Crack the eggs into a separate bowl and beat well with a fork. In a separate bowl mix the breadcrumbs, herbs, lemon zest and the pepper together.
5. Place the fish into the flour and coat evenly. Dip the fish into the beaten egg and then into the breadcrumb mix to cover.
6. Place the fillets on the hot baking sheet and bake for 12 – 15 minutes until they look golden brown
7. While the fish and chips are baking, make the peas as per the recipe on page 25 and mash with a fork when cooked.
8. Serve up the fish, chips and mushy peas (and a side salad for extra vegetables)

SALMON STEAKS & VEGETABLE RISOTTO

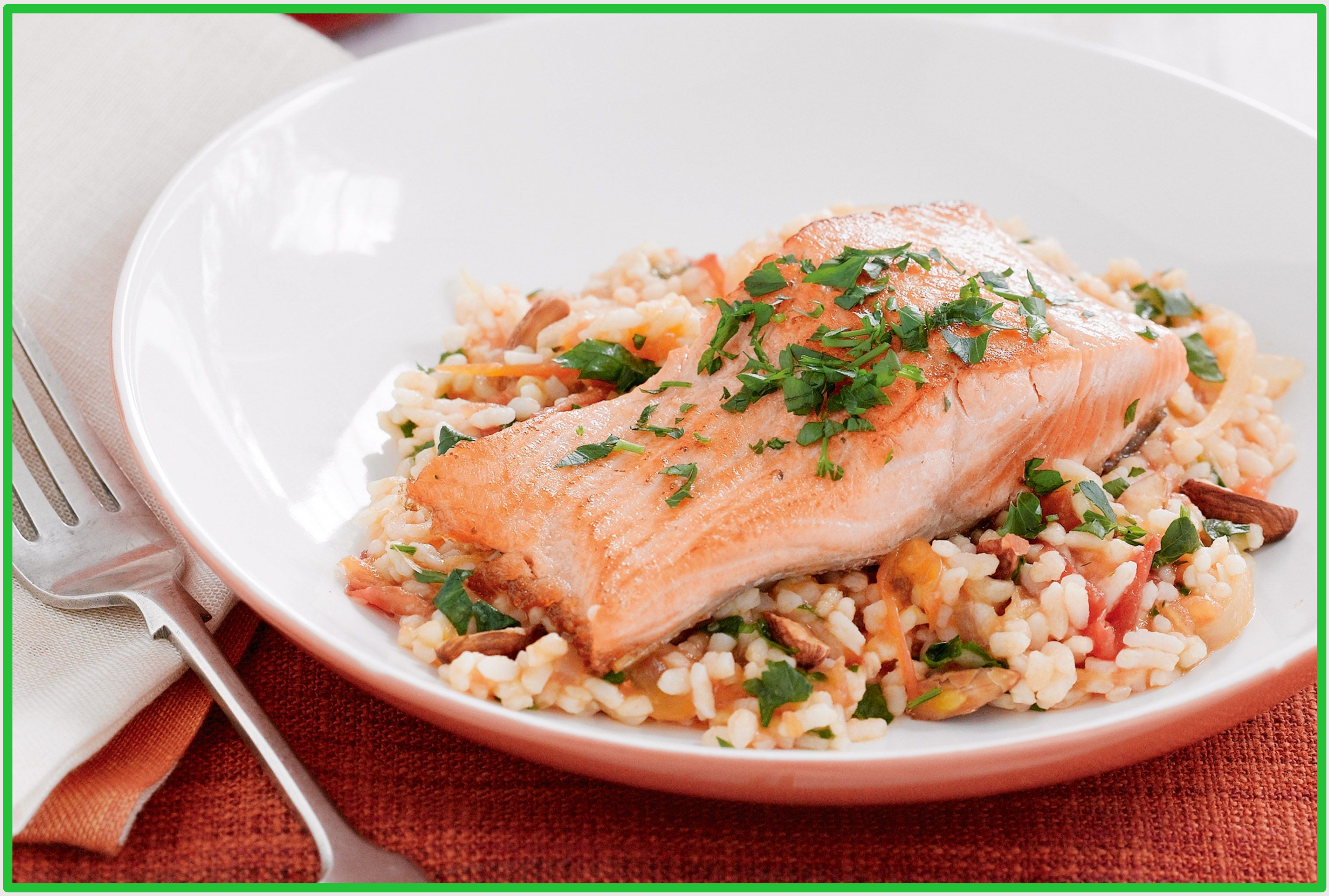

SALMON STEAKS & VEGETABLE RISOTTO

INGREDIENTS

- 1 tbsp vinegar
- 1 tbsp Dijon mustard
- 1 tbsp honey
- Pinch of pepper
- 2 salmon steaks (150g each)
- 3 tbsp olive oil
- 2 leeks, chopped
- 2 medium carrots (finely chopped)
- 150g brown risotto rice
- 750ml low-salt vegetable stock
- 2 celery sticks, chopped
- Handful of fresh dill, finely chopped

MAKES 2 SERVINGS

28g of Protein/serving

INSTRUCTIONS

1. Mix the vinegar, Dijon mustard, honey and seasoning together in a bowl and brush over the salmon
2. Leave the salmon to marinate in the fridge for an hour
3. Add 2 tbsp. olive oil to the pan over a medium heat and add leeks and carrots and cook for 5 minutes, stirring frequently until the leeks have softened then turn the heat down
4. Add the rice to the pan with a good splash of stock and continue adding the stock whilst stirring every so often until the rice is creamy then add the celery. Continue for about 40 minutes
5. Add the dill and 1 tbsp. olive oil to the risotto and season to taste
6. Heat another frying pan to a high heat and lightly brush with olive oil
7. Sear the salmon steaks on each side for 3 minutes and the steaks should be ready when they flake easily when pricked with a fork
8. Serve salmon on a bed of pearl barley risotto.

FISH PIE

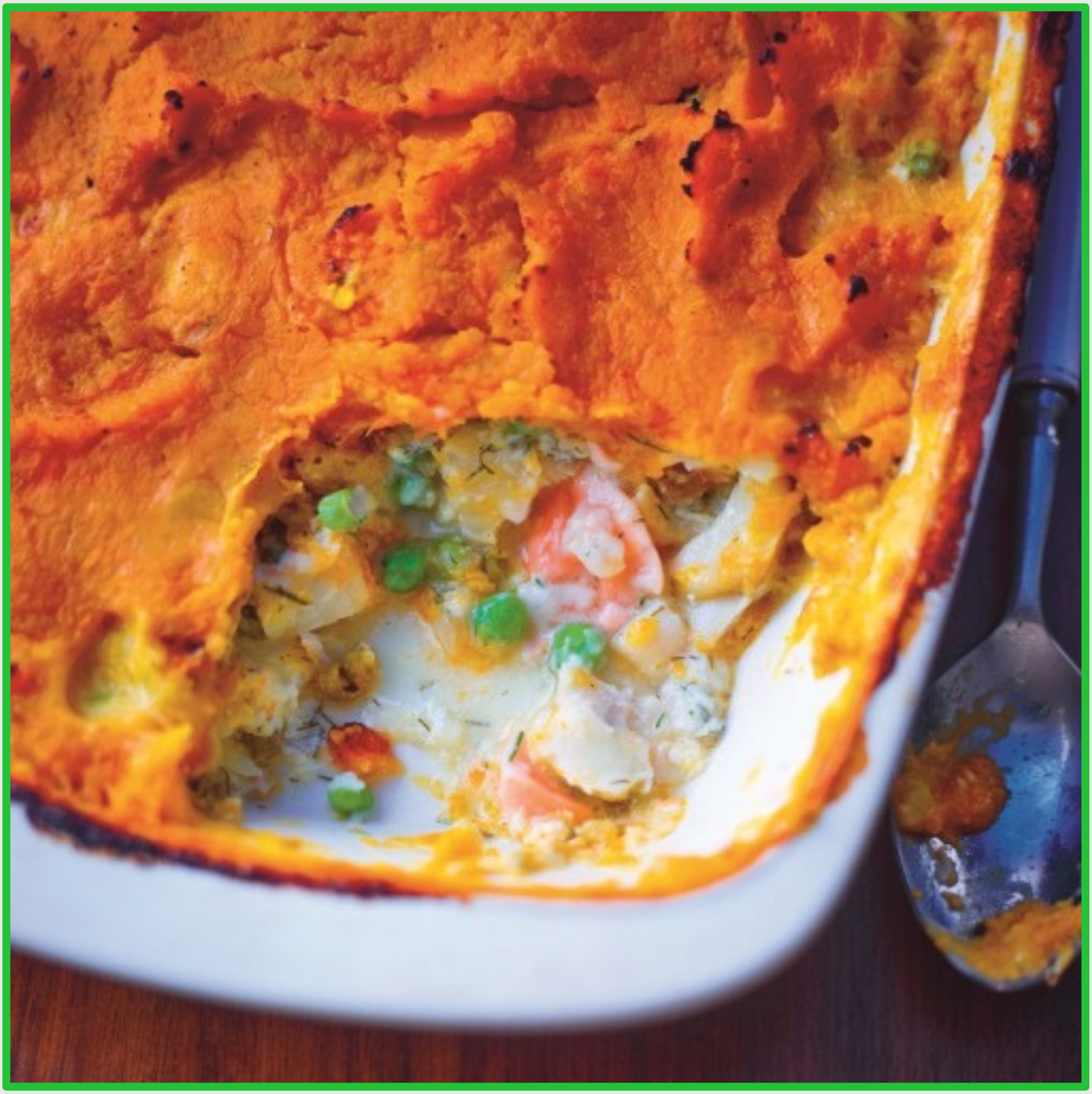

FISH PIE

INGREDIENTS

- 400g haddock or cod, cut into chunks
- 20g raw, frozen prawns
- 1 pack (180g) of Lightest cream cheese (Philadelphia)
- 1 tbsp. flour
- 4 medium sweet potatoes
- 1 medium onion (finely diced)
- 2 medium carrots (finely diced)
- 100g frozen peas
- 4 tbsp. olive oil
- 200ml vegetable stock
- 50g parmesan cheese (grated)
- Salt, pepper and 1 bay leaf

MAKES 4 SERVINGS

38g of Protein/serving

INSTRUCTIONS

1. Preheat the oven to 200°C
2. Peel and chop the sweet potatoes and cook in a microwave on high for 10-15 minutes or until soft (you can also steam them). Mash the potatoes and season with a little salt.
3. Heat the oil in a saucepan and cook the carrots and onions until soft.
4. In a separate bowl, mix the cream cheese and flour and then slowly and gradually add the stock until if forms a thick paste.
5. Add the fish, prawns, bay leaf and peas to the carrot and onion mix, stir well and then add the cream cheese/stock mix and parmesan and mix well. Allow to come to a gentle simmer and then remove from the heat.
6. Add the cooked fish and veg mixture to a baking dish and top with the mashed sweet potato
7. Bake in the oven for 20 minutes and the top starts to brown.
8. Serve with a side salad or some roasted vegetables

BAKED FISH

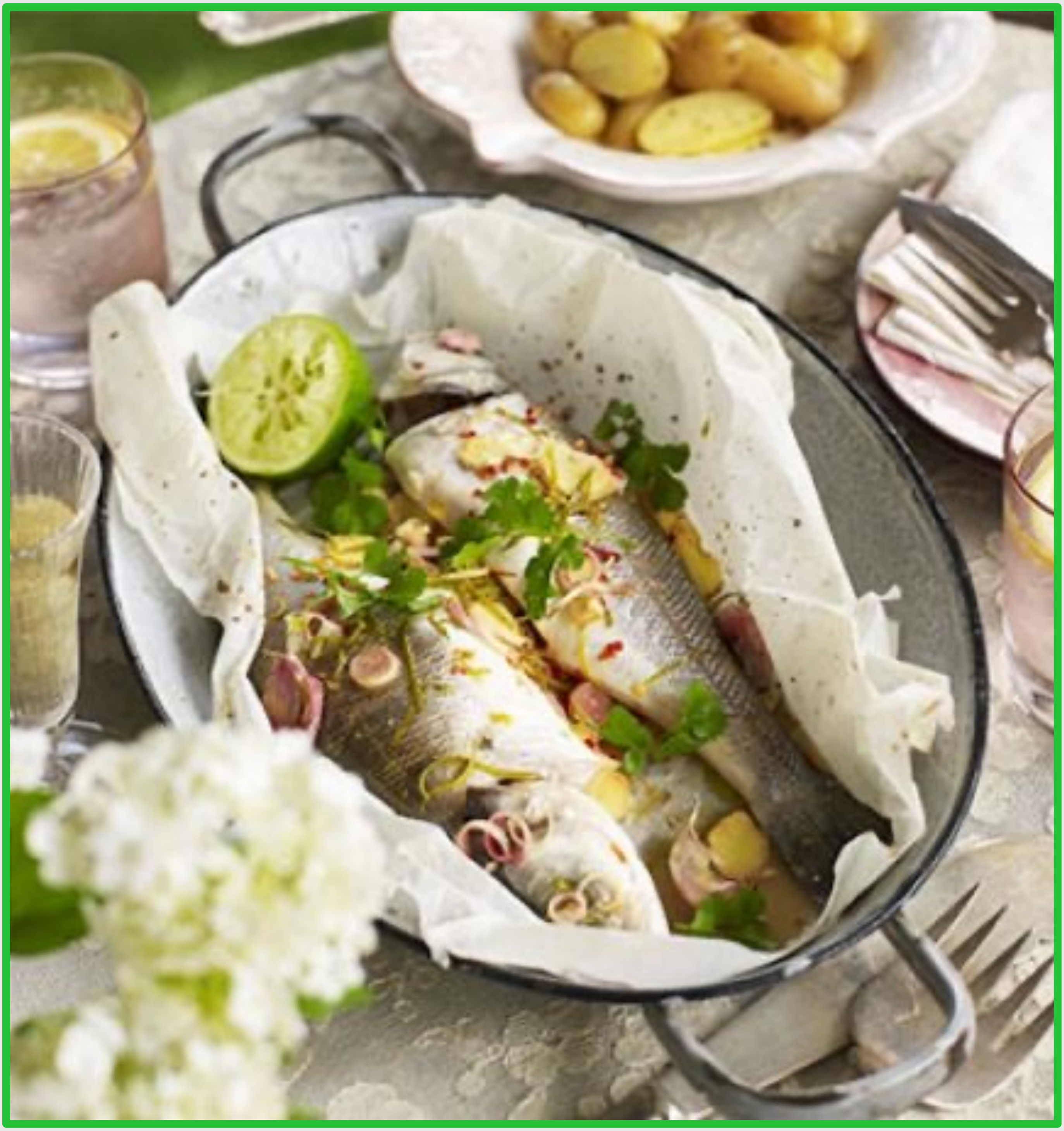

BAKED FISH PARCELS

INGREDIENTS

- 2 small whole fish (e.g. trout, mackerel etc) (gutted)
- 1 lemon cut into thin rings/slices
- 4 cloves of garlic (minced)
- Salt and pepper
- 1 tbsp. Olive oil
- Tin foil

INSTRUCTIONS

MAKES 4 SERVINGS
30g of Protein/serving

1. Preheat the oven to 200°C
2. Take two sheets of tinfoil, big enough to wrap each fish individually
3. Rub the inside and outside of the fish with olive oil, minced garlic, salt and pepper
4. Place the lemon slices in the fish cavity and place the fish on top of the foil sheets and close on top, making the shape of a tent.
5. Place the parcels on a baking tray and bake in the oven for 20 minutes
6. Serve with roasted vegetables or a side salad

ROASTED VEG AND SAUSAGE BAKE

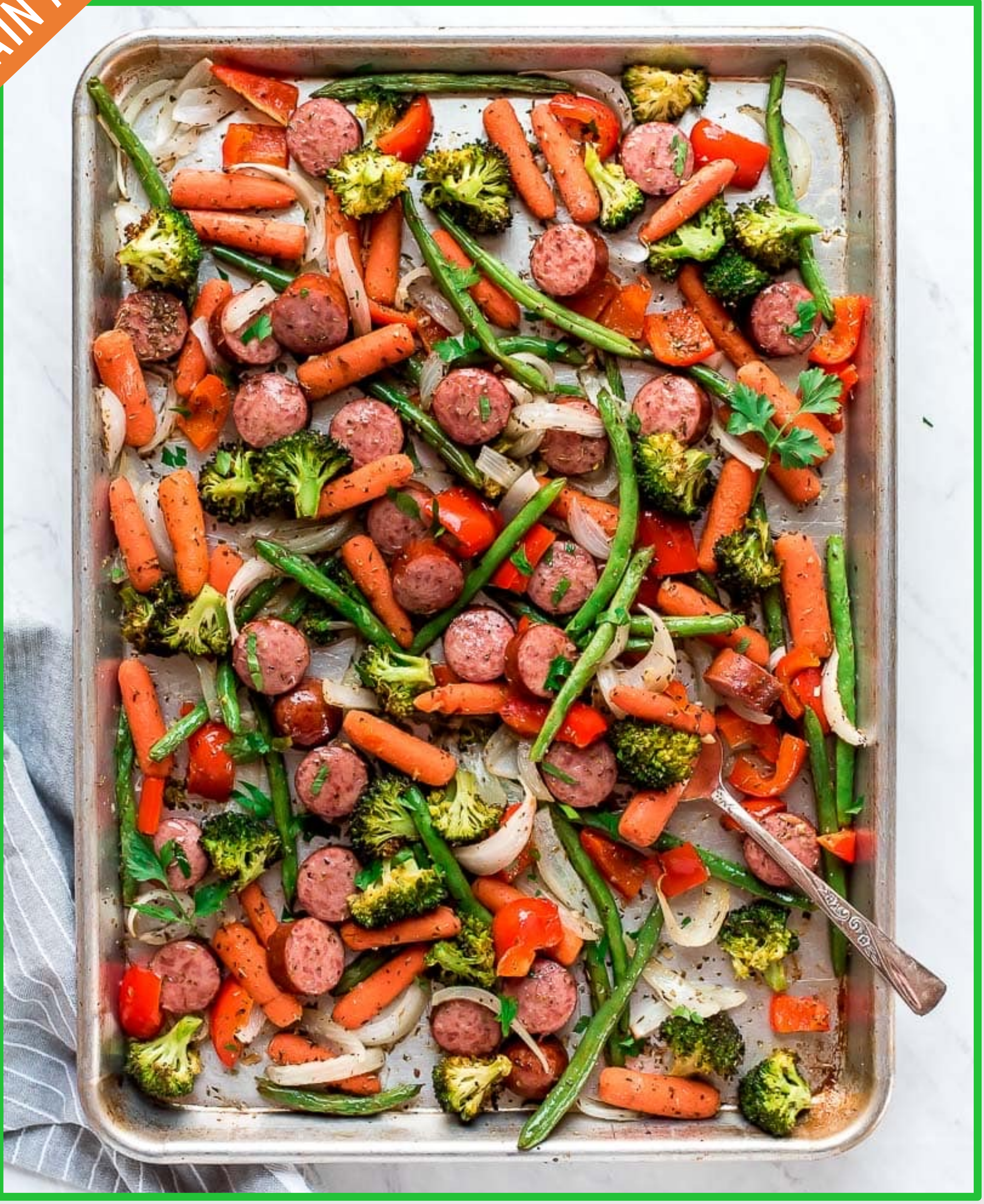

ROASTED VEGETABLE AND SAUSAGE BAKE

INGREDIENTS

- 370g low fat sausages (below 5% fat)
- 2 cups baby carrots (halved, if large)
- 2 cups broccoli florets
- 1 cup sliced onions
- 2 cups green beans
- 1 medium red bell pepper (seeded & cut into 1 inch pieces)
- 2 medium garlic cloves minced
- 3 tablespoons olive oil
- 2 tablespoons Italian seasoning/mized herbs
- 1/2 teaspoon table salt
- 1/2 teaspoon ground black pepper

MAKES 4 SERVINGS

16g of Protein/serving

INSTRUCTIONS

1. Preheat the oven to 200 degrees C.
2. Place the sausages on a plate and microwave for 5 minutes. This will set the sausages a little and make them easier to cut. You could also add them to boiling water for 5 minutes.
3. Cut the sausages into 1-2cm discs.
4. Place all vegetables and sausage into a bowl, drizzle with olive oil and sprinkle with Italian seasoning/mixed herbs, salt, and black pepper. Mix it all together with your hands.
5. Spread out the veggie/sausage mix on a 18x13-inch sheet pan that has been greased with a little olive oil or lined with baking parchment.
6. Bake for 20 to 25 minutes (stirring after 15 minutes) until the vegetables are cooked to fork tender.

PORK & VEGETABLE SKEWERS

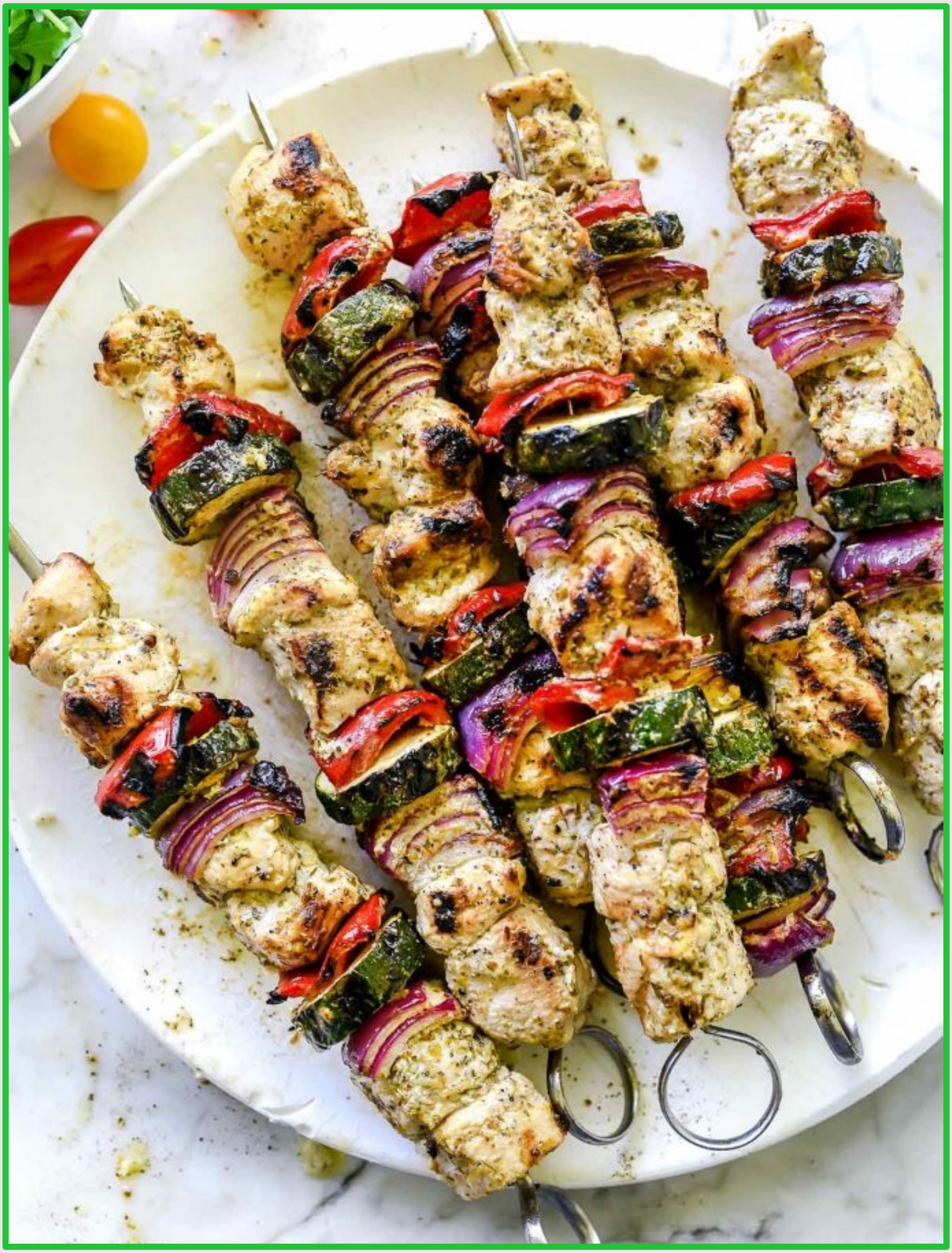

PORK & VEGETABLE SKEWERS

INGREDIENTS

- 250g pork loin or loin chops (cut into large cubes) **(or any meat or fish of choice)**
- ½ a recipe of tzatziki sauce (see page XX)
- 2 tbsp. extra virgin olive oil
- Salt and black pepper
- 1 tsp. mixed herbs
- Metal skewers or wooden skewers soaked in water overnight.
- 1 red onion (quartered into 1-inch pieces)
- 5-6 mushrooms (sliced into 1/4 inch slices)
- ½ red bell pepper (cut into 1-inch pieces)

MAKES 2 SERVINGS
33g of Protein/serving

INSTRUCTIONS

1. Place the pork cubes in a freezer bag or bowl and set aside.
2. Add the tzatziki sauce and marinate the pork for 30 minutes or up to 3 hours in the refrigerator.
3. When ready set the grill to medium high heat.
4. Add the chopped vegetables to a bowl and add the olive oil, salt, pepper and mixed herbs (if using) and toss to mix well.
5. Thread the pork onto the skewers alternating with the red onion, mushrooms and red pepper until you've reached the end of the skewer, ending with pork. Discard any of the remaining marinade that had the pork in it.
6. Place the skewers under the grill and turn often so each side browns and until cooked through, about 10-15 minutes or until the pork juices run clear. Serve warm.
7. Refrigerate leftovers for up to 3 days.

CHILI CON CARNE

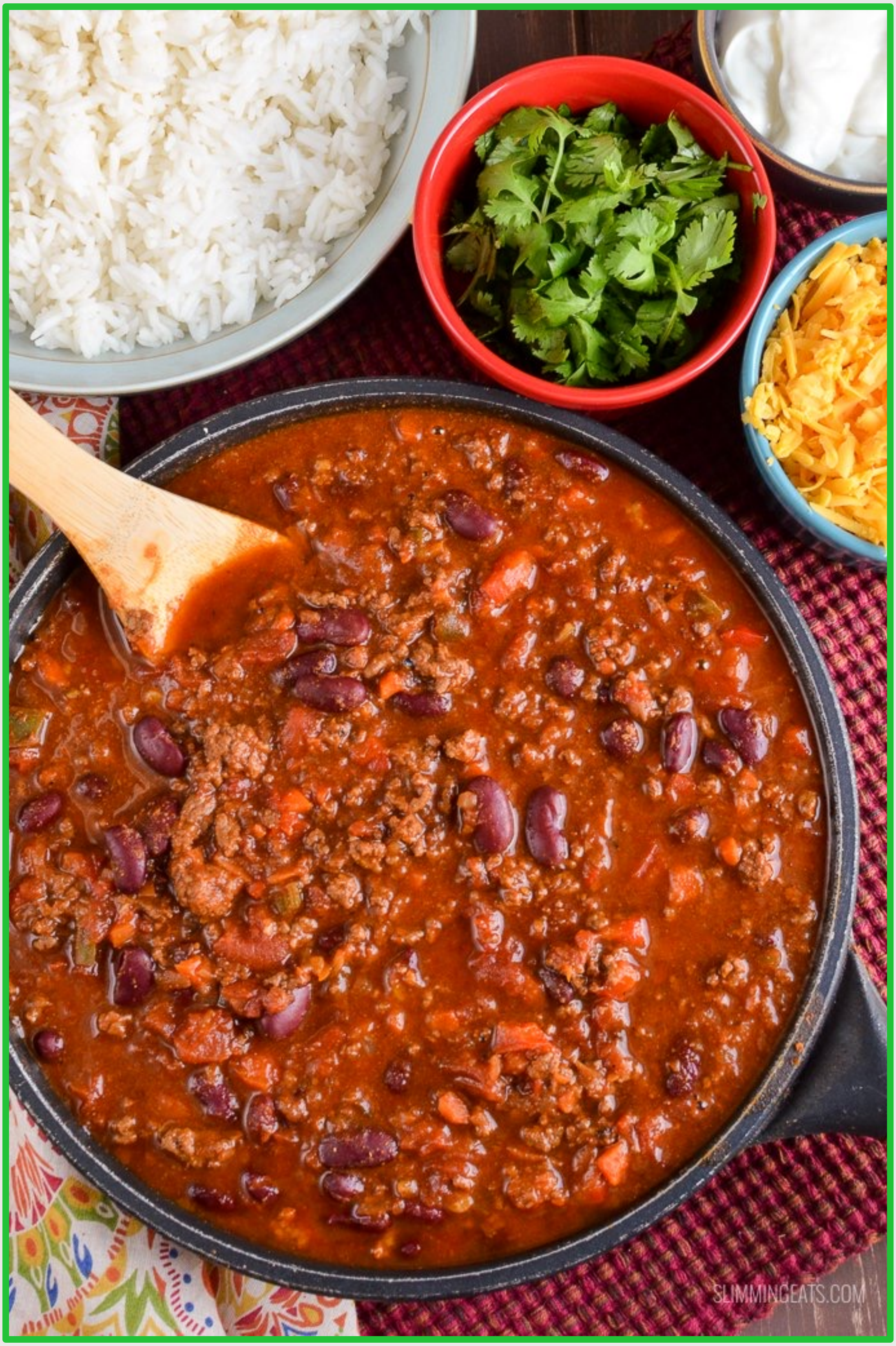

CHILI CON CARNE

INGREDIENTS

- 500g lean minced beef or chicken (<5% fat)
- 4 tbsp. extra virgin olive oil
- 1 onion (finely chopped)
- 1 red pepper (chopped)
- 4 small carrots (finely diced)
- 1 jar of chili con carne sauce
- 1 can of chopped tomatoes
- Salt and black pepper
- 1 large handful of spinach
- 1 400g tin of kidney beans (or beans of choice)
- 2 tbsp. grated reduced-fat cheddar cheese

MAKES 4 SERVINGS
31g of Protein/serving

INSTRUCTIONS

1. Add the chopped carrot to a microwavable bowl and pre-cook on high for 5 minutes (as carrot can take longer to cook than the other vegetables).
2. In a large frying pan or deep pot, heat the oil over a medium heat, add the onion and cook until soft
3. Add the minced meat and cook until brown, stirring constantly.
4. Add the carrots and red pepper and stir well.
5. Add the jar of chili con carne sauce, chopped tomatoes and the beans and allow to simmer. The dish is cooked now but the longer you allow it to simmer on a low heat the better the flavour.
6. When ready, season the dish with salt & pepper and add the spinach to the sauce and stir until wilted and mixed through.
7. Serve the chilli con carne over some brown rice, pasta, a baked potato or even some cooked vegetables.
8. Top with the cheese and serve.

WHOLEWHEAT SPAGHETTI BOLOGNESE

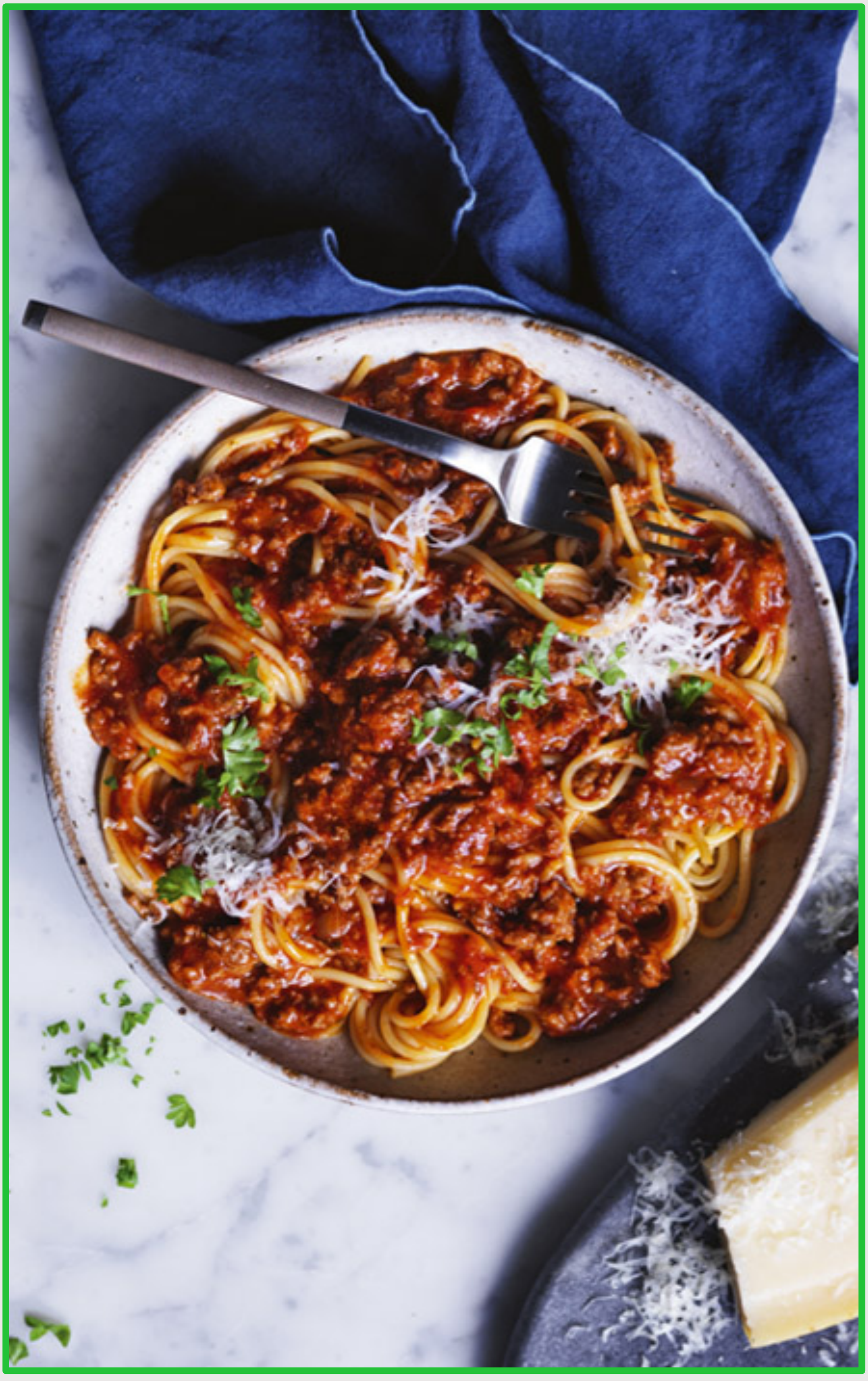

WHOLEWHEAT SPAGHETTI BOLOGNESE

INGREDIENTS

- 500g lean minced beef or chicken (<5% fat)
- 3 tbsp. extra virgin olive oil
- 1 jar of Bolognese sauce
- 4 handfuls of courgette noodles (or other vegetable noodle)
- 4 medium carrots (finely diced)
- Salt and black pepper
- 100g Wholewheat spaghetti
- 1 small onion (chopped)
- 1 large handful of spinach
- 1 400g tin of lentils (or beans of choice)
- 1 tbsp. grated Parmesan cheese

MAKES 4 SERVINGS
29g of Protein/serving

INSTRUCTIONS

1. In a frying pan, heat the oil over a medium heat, add the onion and cook until soft
2. Add the minced meat and cook until brown, stirring constantly.
3. Add the jar of tomato sauce, the lentils and the diced carrots and allow to simmer while you prepare the spaghetti.
4. Bring a large pot of salted water to a boil. Cook the spaghetti according to package directions and add the courgette noodles for the last minute. Use tongs to transfer the cooked spaghetti to a strainer.
5. Add the spinach to the pasta sauce and stir until wilted and mixed through.
6. Divide the noodles onto 4 plates and top evenly with the meat sauce.
7. Top with the Parmesan cheese and serve.

HEART HEALTHY BURGERS

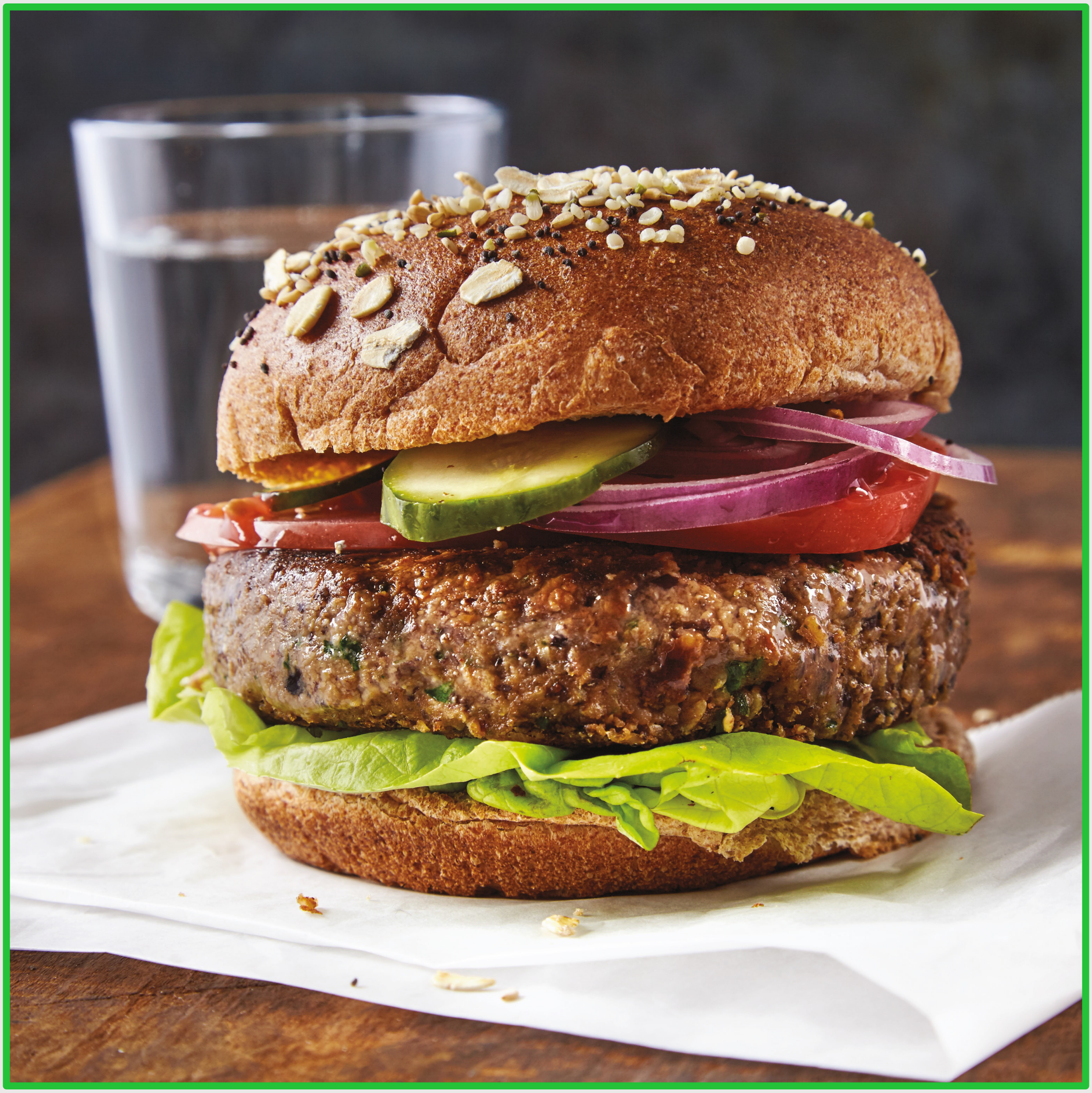

HEART HEALTHY BURGERS

INGREDIENTS

- 2 low fat beef burger patties (less than 5% fat)
- 2 wholemeal bread rolls
- 2 slices reduced-fat cheddar cheese
- Low-fat sauce of choice (ketchup, barbecue, sweet chilli etc.)
- Olive oil for greasing
- Large handful of salad leaves of choice (spinach, lettuce, rocket etc.)
- 1 cup of salad vegetables of choice (peppers, tomatoes, cucumber etc.) (sliced thinly)
- Pickles (sliced) (optional)
- 2 tbsp. extra virgin olive oil

MAKES 2 SERVINGS
41g of Protein/serving

INSTRUCTIONS

1. Prepare the salad by mixing the leaves and vegetables in a bowl with the olive oil and some salt and pepper.
2. Heat a frying pan to a medium heat with a little olive oil. Add the patties and cook on each side until browned and cooked to your liking.
3. While the burgers are cooking, slice the bread roll and toast under a grill.
4. 1 minute before the burgers are finished cooking, add a slice of cheese on top and cover the frying pan with a lid to melt the cheese slightly.
5. Place the burgers on the buns, add sauce of choice, pickles and enough salad to cover the burger and top with the other half of the bun
6. Serve immediately with the remaining salad.

*This would go great with some of the coleslaw (page 32) or tomato salsa (page 34)

CHICKEN FRIED RICE

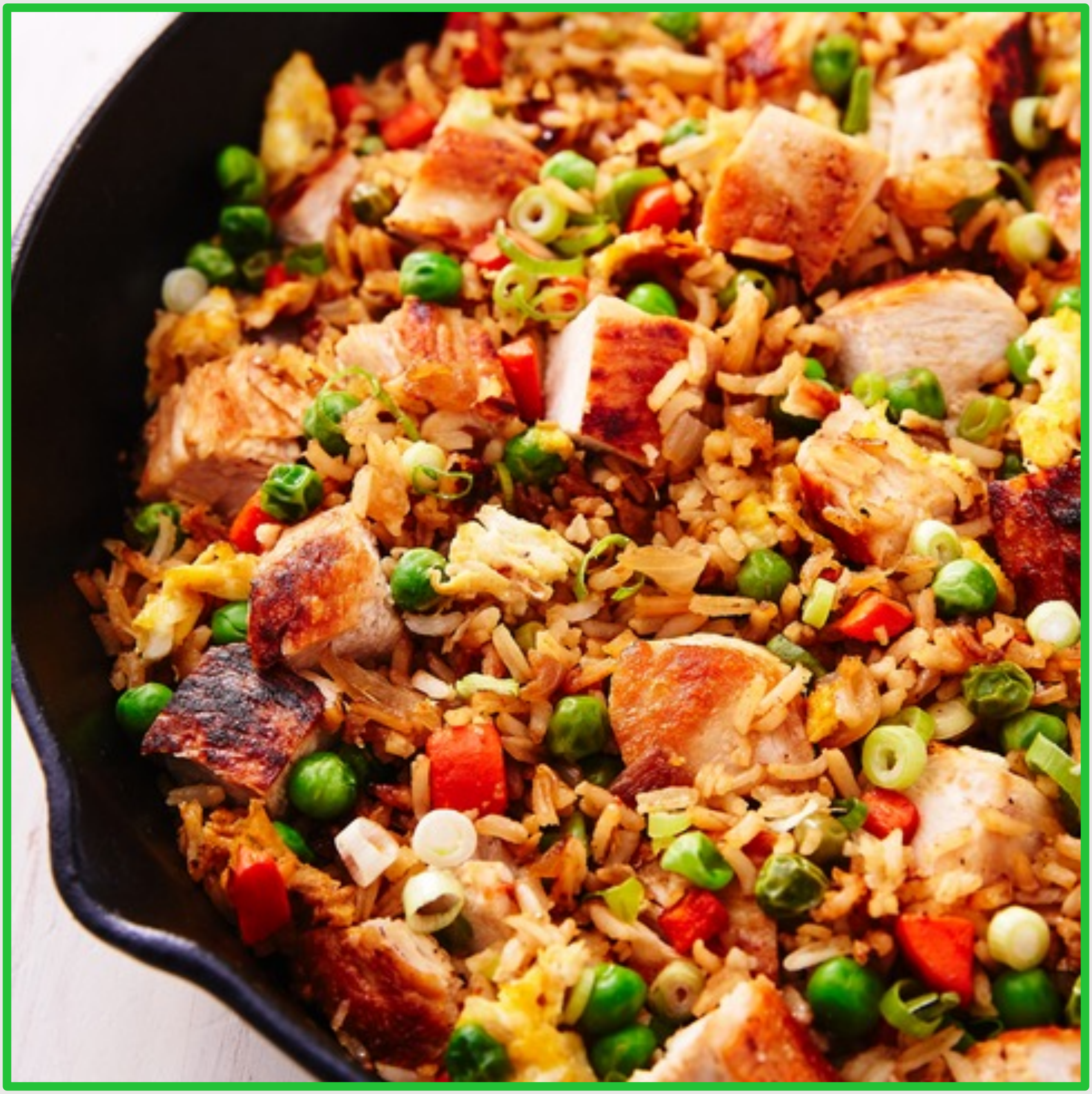

CHICKEN FRIED RICE

INGREDIENTS

- 4 tbsp. extra-virgin olive oil
- 500g chicken breasts (about 2 large breasts)
- Salt & black pepper
- 1 tbsp. sesame oil
- 1 medium onion, chopped
- 2 carrots, peeled and diced
- 3 cloves garlic, minced
- 1 tbsp. freshly minced ginger
- 4 c. cooked brown rice (or 2 microwave packs) (less if desired)
- 150g. frozen peas
- 2 large eggs, beaten
- 2 tbsp. soy sauce
- 2 green onions, thinly sliced
- 2 large handfuls of spinach

MAKES 4 SERVINGS

40g of Protein/serving

INSTRUCTIONS

1. In a medium skillet over medium heat, heat the olive oil.
2. Chop the chicken into bite-size chunks and season with salt and pepper, then add to skillet, and cook until golden and no longer pink. Remove from skillet and set aside.
3. To the same skillet, heat ½ a tablespoon sesame oil. Add the onion and carrots and cook until soft, 5 minutes. Add the garlic and ginger and cook until fragrant, 1 minute more.
4. Stir in the rice and peas and cook until warmed through, 2 minutes, stirring continuously.
5. Push rice to one side of skillet and add the remaining sesame oil to the other side. Add the eggs and stir until almost fully cooked, then fold eggs into rice. Add chicken back to skillet with soy sauce, spinach and green onions and stir to combine.

EASY CHICKEN OVEN BAKE

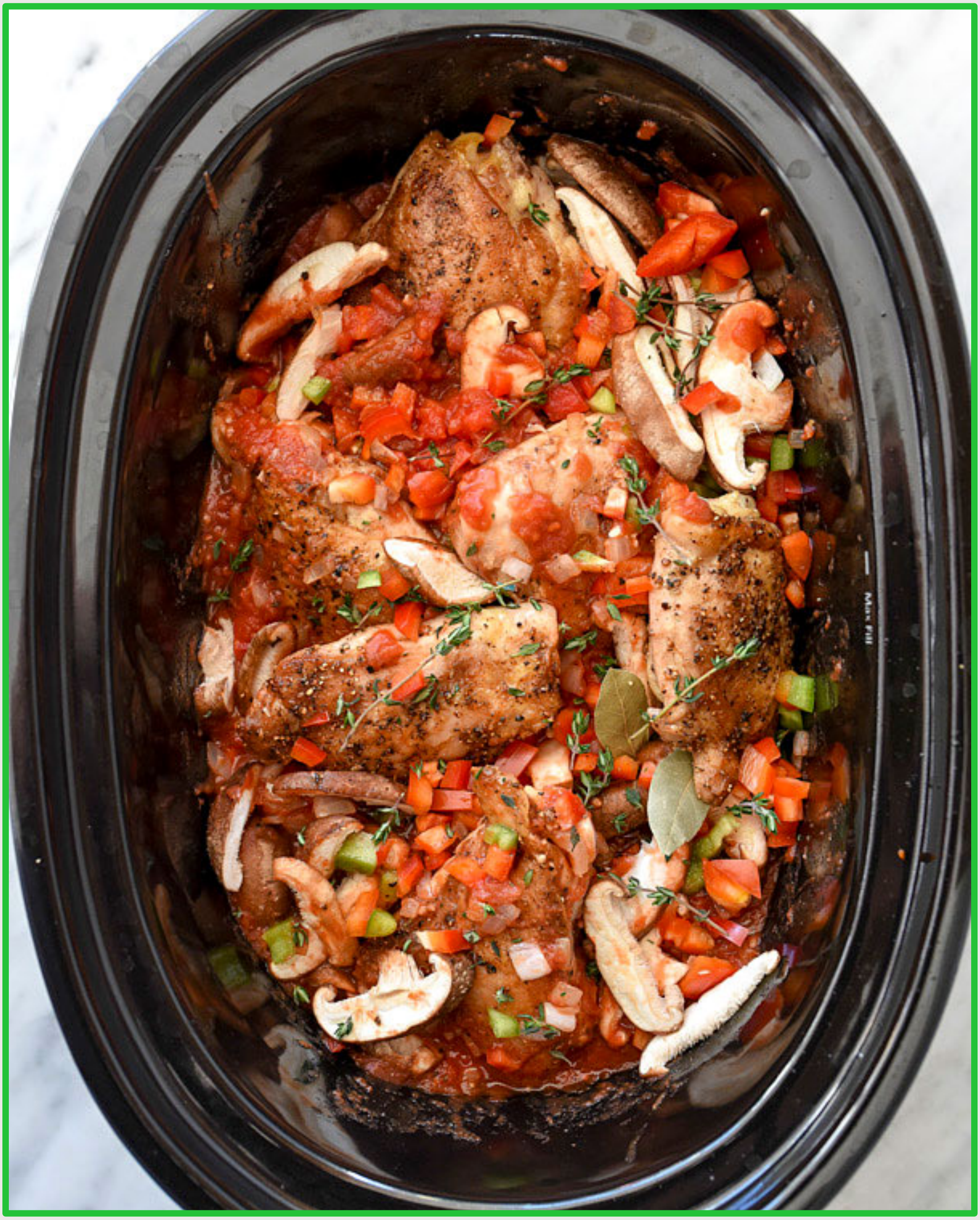

EASY CHICKEN OVEN BAKE

INGREDIENTS

- 500g of meat/fish of choice (lean stewing beef, pork loin, skinless chicken thighs, cod, salmon) (chopped into cubes/chunks)
- 1 500g jar of tomato-sauce (dolmio-style)
- 4 tbsp. extra virgin olive oil
- Salt and black pepper
- 2 medium carrots (chopped into small chunks)
- 1 green pepper (finely chopped)
- 1 medium onion (finely chopped)
- 1 stick celery (finely chopped)
- 100-200g of mushrooms (sliced)

MAKES 4 SERVINGS
30g of Protein/serving

INSTRUCTIONS

1. Pre-heat the oven to 180 degrees C.
2. Fry the meat in a frying pan with half the olive oil until browned on all sides but not fully cooked.
3. Add the meat or fish to an oven proof dish and add the remaining ingredients (including the olive oil). Season with salt and pepper and mix well.
4. Cover the dish with tinfoil and bake in the oven for 1 hour and thirty minutes.
5. Check the vegetables are cooked to your liking and remove dish from oven.
6. Serve immediately.

*These meals can frozen and defrosted later for a quick and easy supper

SCOUSE

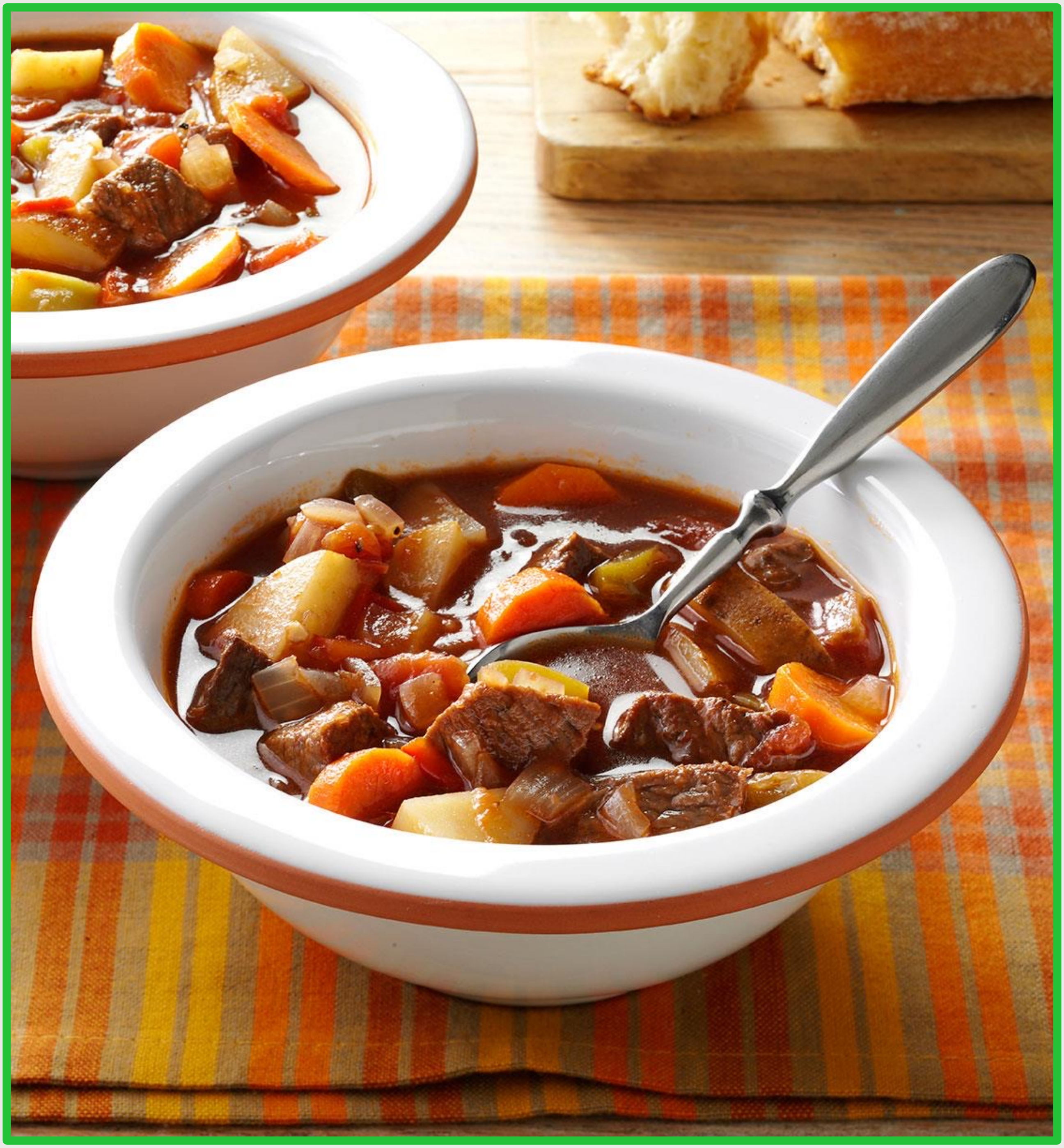

SCOUSE

INGREDIENTS

- 500g lean stewing beef (below 5%)
- 4 tbsp. olive oil
- salt and pepper
- 1 large onion, diced
- 1 beef stock cube
- 500ml hot water
- 2 bay leaves
- 4 large carrots, cut into 15mm chunks
- 6 medium potatoes, peeled and chopped into 2cm chunks
- Worcestershire sauce
- pickled red cabbage, to serve

MAKES 4 SERVINGS
27g of Protein/serving

INSTRUCTIONS

1. Dice the meat into whatever size you prefer.
2. Heat the oil in the pan to a medium temperature and add the meat. Season very well with salt and pepper. Stir until brown all over. Add the onions and stir on a medium heat for around 10 minutes; the onions must not brown.
3. Dissolve the stock cubes in the water, add to the pan and bring to the boil.
4. Add the bay leaves, stir, cover and when bubbling, reduce to medium heat. You want the stock to bubble but not violently for 1 hour.
5. Add the carrots and potatoes, turn up the heat until bubbling throughout and reduce slightly. Leave for another 45 minutes to 1 hour with the lid off the pan so it reduces.
6. The stew should reduce once the veg is added but you may need to add more water or perhaps turn the heat up to ensure the liquid is as thick as gravy.
7. Taste and season again if necessary (add 2 tbsp. of Worcestershire sauce or to taste). Serve in a bowl with the red cabbage and plenty of juice on the top.

EASY BBQ CHICKEN & VEG PIZZA

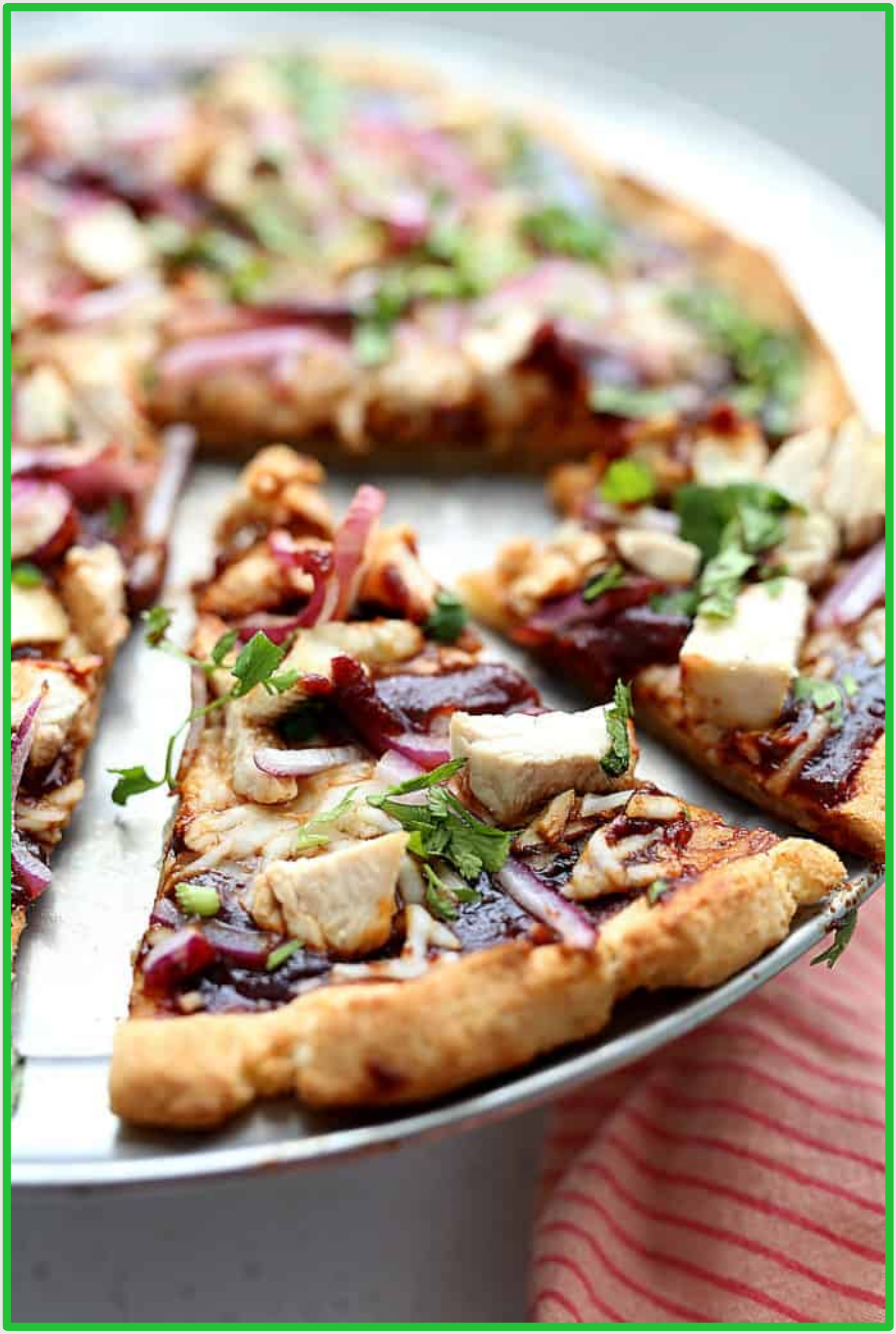

EASY BBQ CHICKEN & VEG PIZZA

INGREDIENTS

- 2 large wholemeal pitta breads (any wholemeal flat-bread will do)
- 2 tbsp. tomato paste or passata
- 2 tbsp. barbecue sauce
- 1 pack (125g) of reduced-fat mozzarella cheese (finely chopped)
- 40g reduced fat grated cheese
- Half a small onion, finely sliced
- Half a bell pepper, finely diced (or any vegetable of choice)
- 1 tbsp. olive oil
- 100g chicken breast (chopped)
- Salt, pepper and chopped basil

INSTRUCTIONS

MAKES 2 SERVINGS
36g of Protein/serving

1. Preheat the oven to 200°C
2. Heat the oil in a frying pan and quickly cook the chicken until it is cooked through. Add salt and pepper to taste and set aside.
3. Split the pittas length wise so you have two thin, pizza rounds from each pitta. Place them, inner surface facing up, on an oven tray
4. Mix the tomato paste/passata with the barbecue sauce and spread evenly over the pitta halves.
5. Sprinkle the cheeses evenly over the pitta halves, followed by the vegetables and the cooked chicken
6. Bake in the oven for about 10 minutes until the cheese has melted and started to take a golden color.
7. Serve immediately with a side salad.

CHICKEN & MUSHROOM RISOTTO

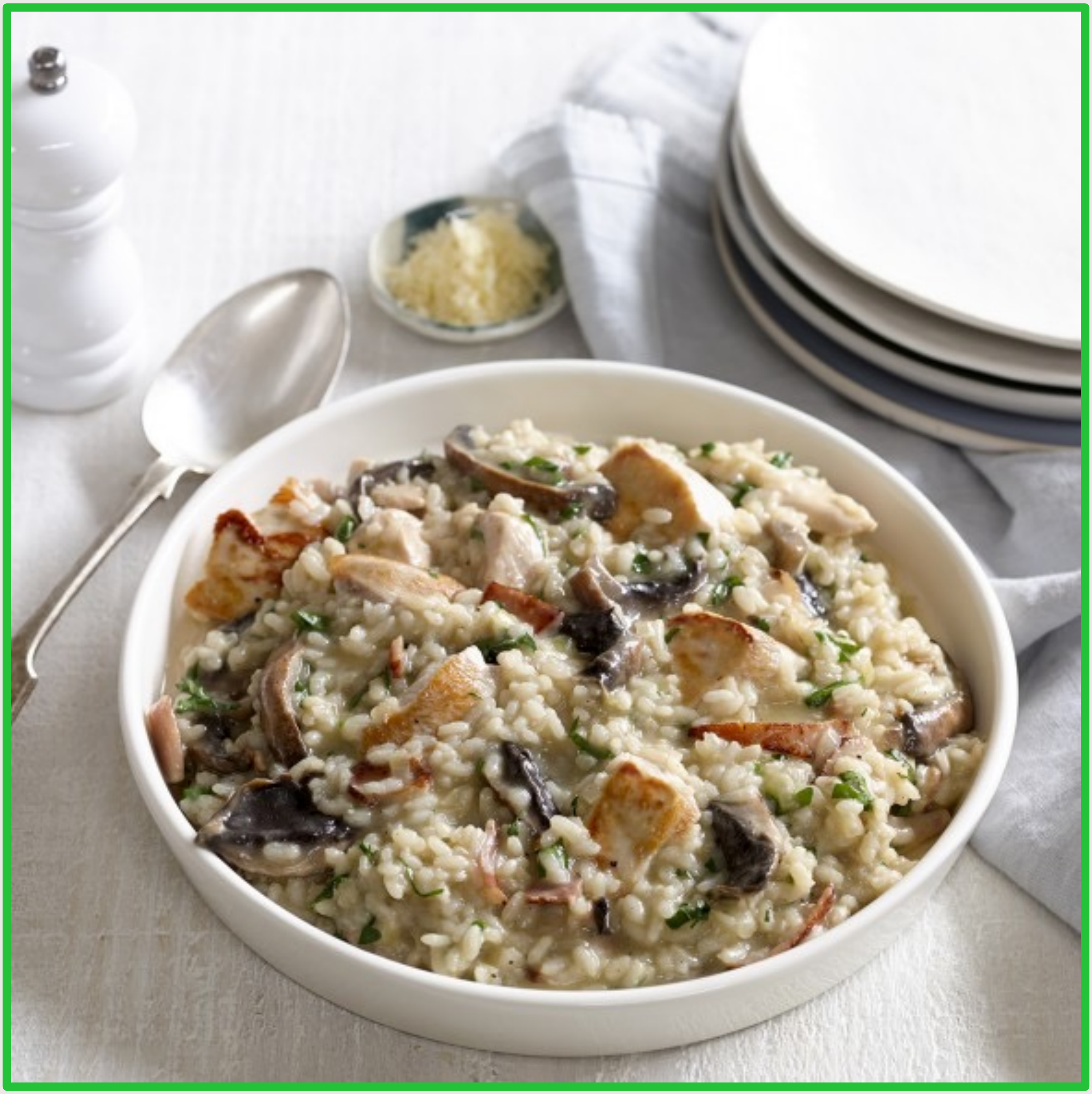

CHICKEN & MUSHROOM RISOTTO

INGREDIENTS

- 1 medium onion, peeled and chopped
- 4 tbsp olive oil
- 300g chicken breast, cut into pieces
- 2 celery stalks, finely diced
- 2 cloves of garlic, crushed
- 200g mushrooms, sliced
- ¼ cup of white wine (optional)
- 200g brown risotto (Arborio) rice
- 1L of low-salt vegetable or chicken stock
- 3 large handfuls of spinach, chopped
- 120g green beans
- ¼ cup of parmesan, grated
- 1 pack (180g) lightest cream cheese

MAKES 4 SERVINGS

33g of Protein/serving

INSTRUCTIONS

1. Fry the onion with 2 tbsp of olive oil in a wide, deep non-stick pan on a medium heat for 3-5 minutes
2. Add the chicken and fry until cooked through and meat turns white.
3. Add the celery, garlic and mushrooms and cook until soft. Add the wine (if using) and the rice to the pan
4. Add a ladle at a time of the stock to the rice & veg and continue doing so slowly until the liquid has almost evaporated/fully absorbed and continue stirring
5. Whilst waiting for the wine to absorb the liquids, steam the green beans in a separate saucepan for 3-5 minutes until tender
6. Slice green beans and add to the risotto when there is a little bit of stock left for the rice to absorb
7. When the rice is cooked through, season to liking, add the parmesan, cream cheese, spinach and 2 tbsp. of olive oil. Stir well and serve

HEALTY BANGERS & MASH

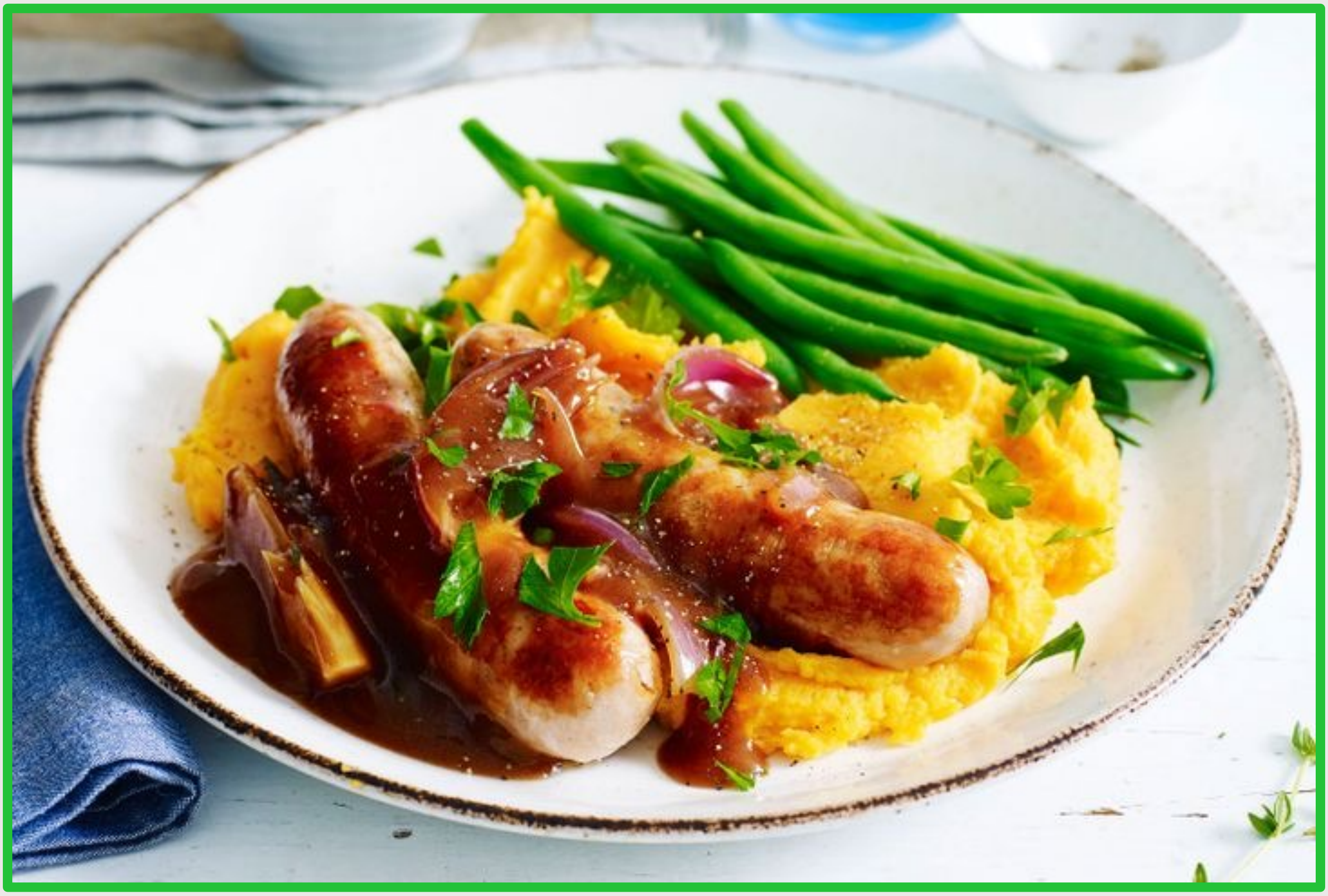

HEALTY BANGERS & MASH

INGREDIENTS

- 1 medium onion, peeled and chopped
- 400g white potatoes, cut into chunks
- 300g carrots, chopped
- 6 tbsp olive oil
- 2 onions, sliced
- 5 garlic cloves, sliced
- Salt and black pepper
- 720ml low-salt vegetable or chicken stock
- Pinch of black pepper
- Pinch of dried thyme
- 1 tbsp. tomato puree
- 8 very low-fat (below 5% fat) sausages
- 400g green beans (fresh or frozen)

MAKES 4 SERVINGS

23g of Protein/serving

INSTRUCTIONS

1. Cook potatoes and carrots in boiling water (or steam) for 20 – 25 minutes and drain well
2. Meanwhile, make the gravy in a large saucepan by adding 2 tbsp. olive oil to the pan, add the onion and the garlic and cook until golden adding a small amount of stock to prevent burning
3. Add the rest of the herbs, tomato puree the remaining stock to the onions and bring to the boil then reduce heat and simmer for 30 – 40 minutes
4. In a medium sized pan bring water to the boil and add in the green beans. Boil (or steam) for 4 minutes.
5. Mash up the potatoes, carrot, 4 tbsp. olive oil and garlic and reheat whilst constantly stirring
6. Cook the sausages in a 200° oven or 20 minutes until golden brown
7. Serve up the mash, sausages and green beans with the gravy

JERK CHICKEN WITH RICE & PEAS

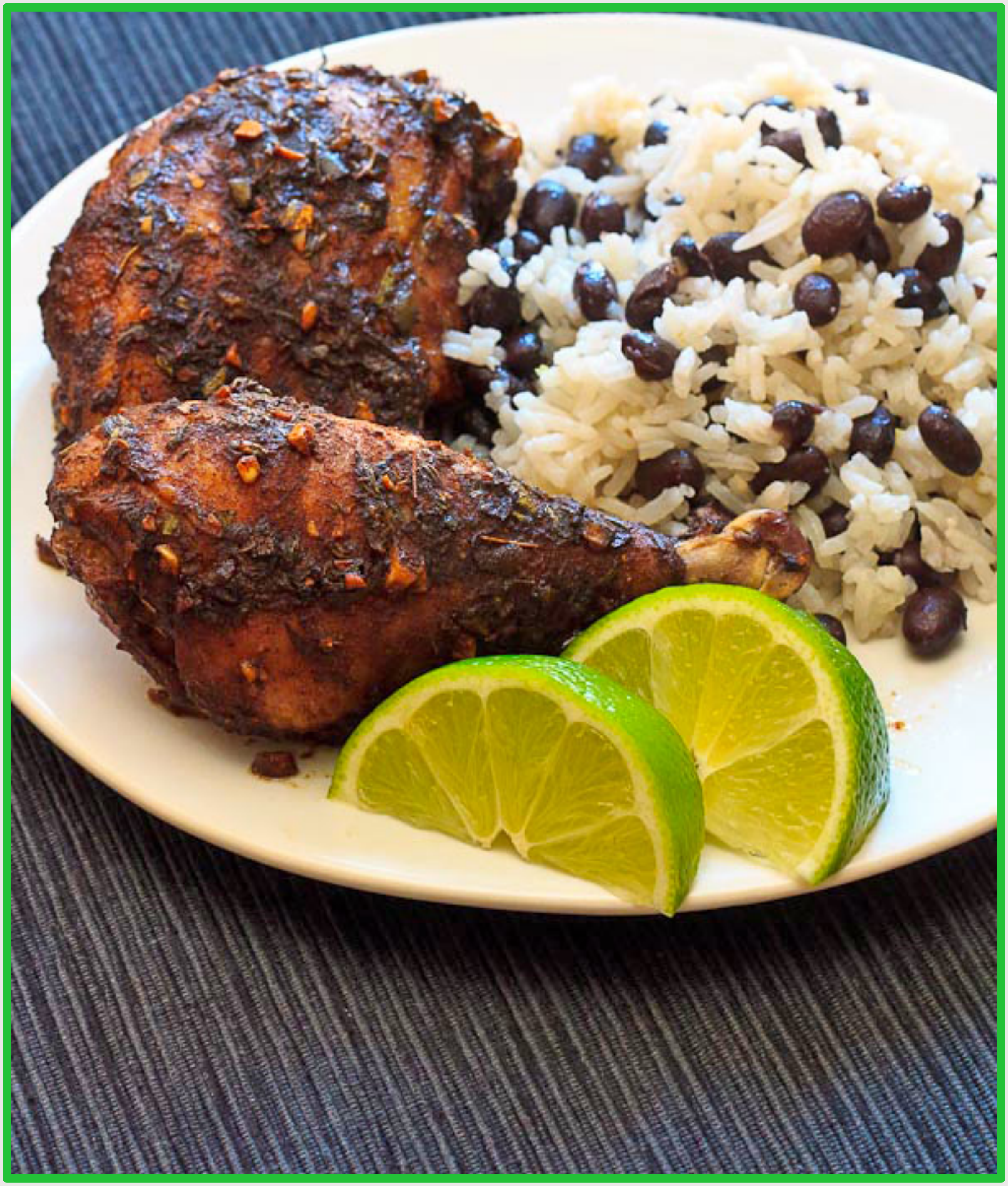

JERK CHICKEN WITH RICE & PEAS

INGREDIENTS

- 400g Chicken breast
- 1 Lime
- 200g Wholegrain basmati rice
- 1 can (400ml) Light coconut milk
- Bunch of spring onions, chopped
- 3 tsp Garlic granules
- Salt and black pepper
- 1½ tsp Dried thyme
- 1 x 400g tin kidney beans (drained and rinsed
- 100ml pre-made jerk marinade/sauce

INSTRUCTIONS

MAKES 4 SERVINGS
39g of Protein/serving

1. Preheat the oven to 180°C
2. Cut each chicken breast into 2 large strips and pour the marinade over the meat, ensuring it is well coated. Cover and leave to marinate in the fridge for an hour (or overnight)
3. Put the chicken pieces in a roasting tin with the lime halves and cook for 35-40 minutes until tender and cooked through.
4. While the chicken is cooking, rinse the rice in plenty of cold water, then tip it into a large saucepan with all the remaining Ingredients: except the kidney beans. Add 165ml cold water and set over a high heat. Once the rice begins to boil, turn it down to a medium heat, cover and cook for 25 minutes.
5. Add the beans to the rice, then cover with a lid. Leave off the heat for 5 minutes until all the liquid is absorbed.
6. Squeeze the roasted lime halves over the chicken and serve with the rice & peas.
7. Serve with a side salad or some healthy coleslaw

COTTAGE PIE

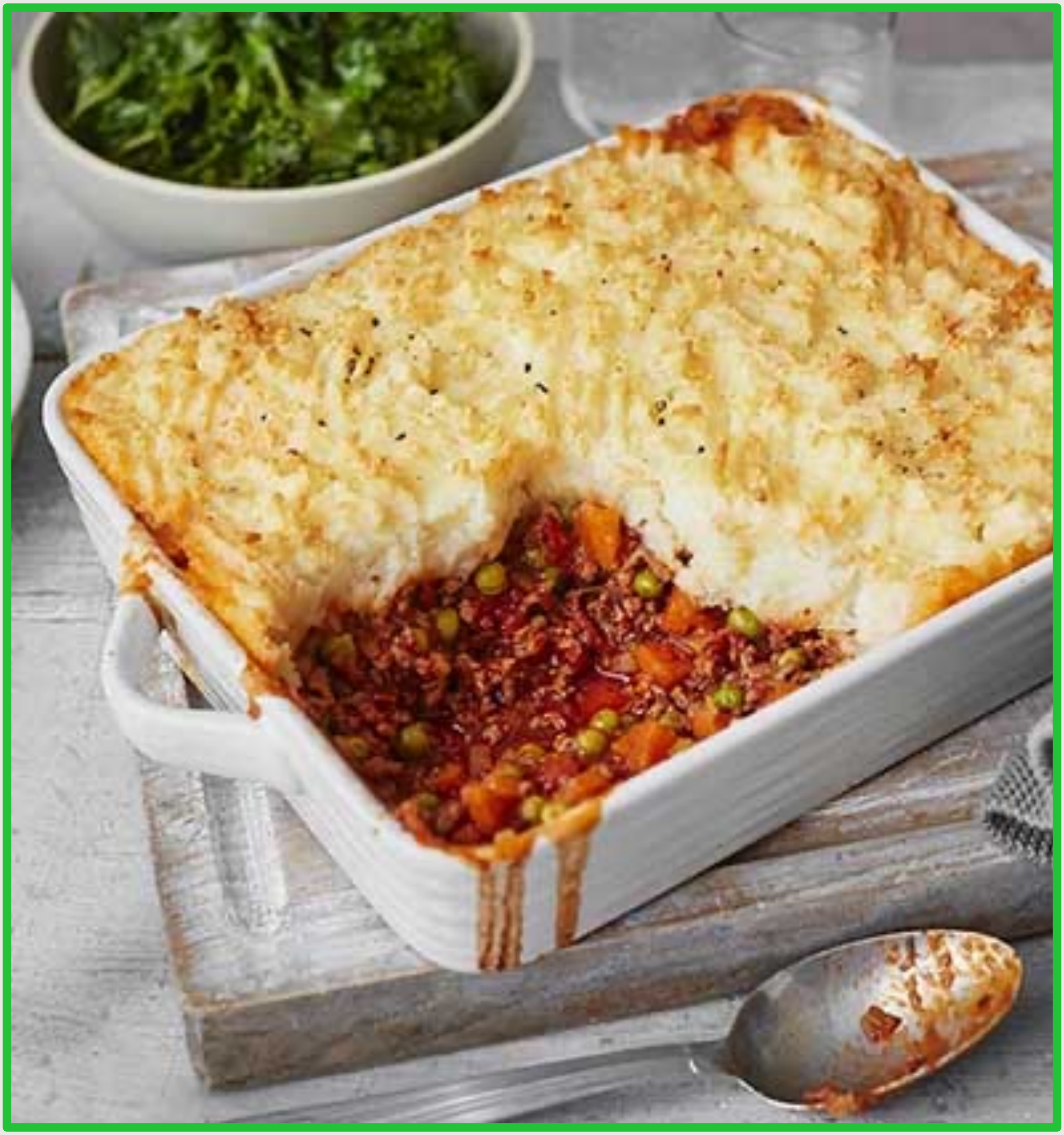

COTTAGE PIE

INGREDIENTS

- 3 large potatoes, chopped into large chunks
- 4 tbsp olive oil
- 6 spring onions finely sliced
- 4 carrots, grated
- 500g lean beef/turkey/pork mince (less than 5% fat)
- 40g reduced-fat cheddar cheese (grated)
- 2 tbsp flour
- 200g frozen peas
- 200ml low-salt vegetable stock
- 6 tbsp Worcestershire sauce
- 100g mushrooms, sliced
- 1 red chilli, finely sliced

MAKES 4 SERVINGS

32g of Protein/serving

INSTRUCTIONS

1. Microwave the potatoes for 4 minutes, rest for 2 minutes and then microwave for a further 4 minutes.
2. Meanwhile, heat olive oil in a large frying pan over a high heat. Add spring onions, carrot, mushrooms and chilli. Fry for 30 seconds, stirring almost constantly.
3. Add mince and cook until brown.
4. Sprinkle in the flour. Stir the mix with the rest of the ingredients and quickly follow with the peas and the beef stock
5. Reduce the heat and stir until the sauce has thickened.
6. Remove the pan from the heat and stir through the Worcestershire sauce.
7. Mash the potato, season with salt and pepper and spoon onto the pie mix and sprinkle with the cheese
8. Place into the oven and cook until the top is golden and crispy

GREEK YOGHURT "ICE CREAM" BARS

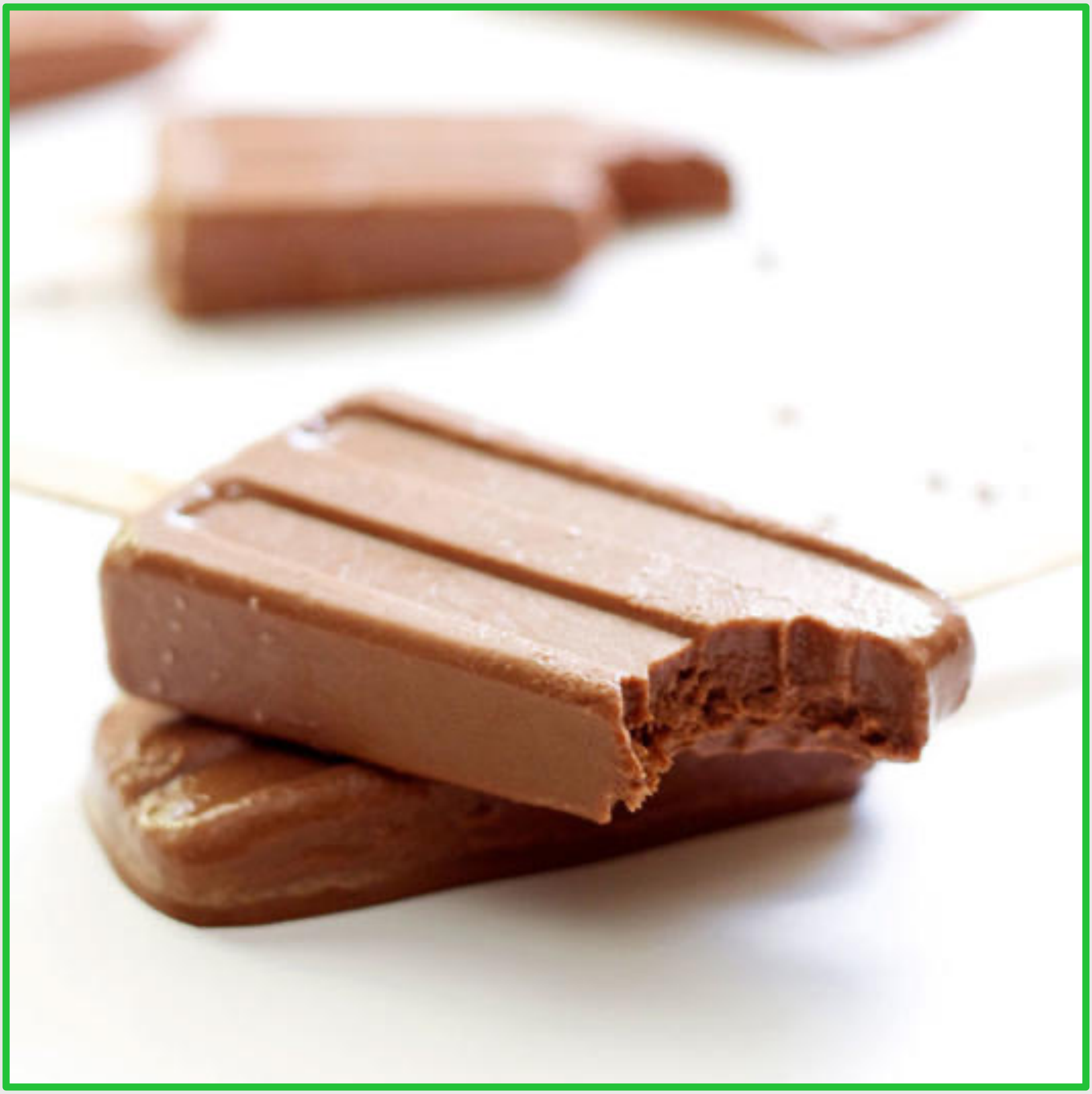

GREEK YOGHURT “ICE CREAM” BARS

INGREDIENTS

- 250g fat-free Greek yoghurt or quark
- 250mls semi skimmed milk
- 50g cocoa powder
- 2 tsp vanilla essence
- Granulated artificial sweetener of choice (to taste)
- Ice cream freezer moulds

MAKES 5 SERVINGS
6g of Protein/serving

INSTRUCTIONS

1. Add all the ingredients (except the sweetener to a bowl and mix well with a whisk.
2. Gradually add the sweetener and keep tasting until you get the right amount of sweetness.
3. Pour the mixture into moulds leaving a little space at the top for expansion in the freezer.

*Let the bars sit out at room temperature for 5-10 minutes before trying to release them from the mould. You can also run the moulds under hot water for a few seconds to release the bars.

CARROT CAKE MUFFINS

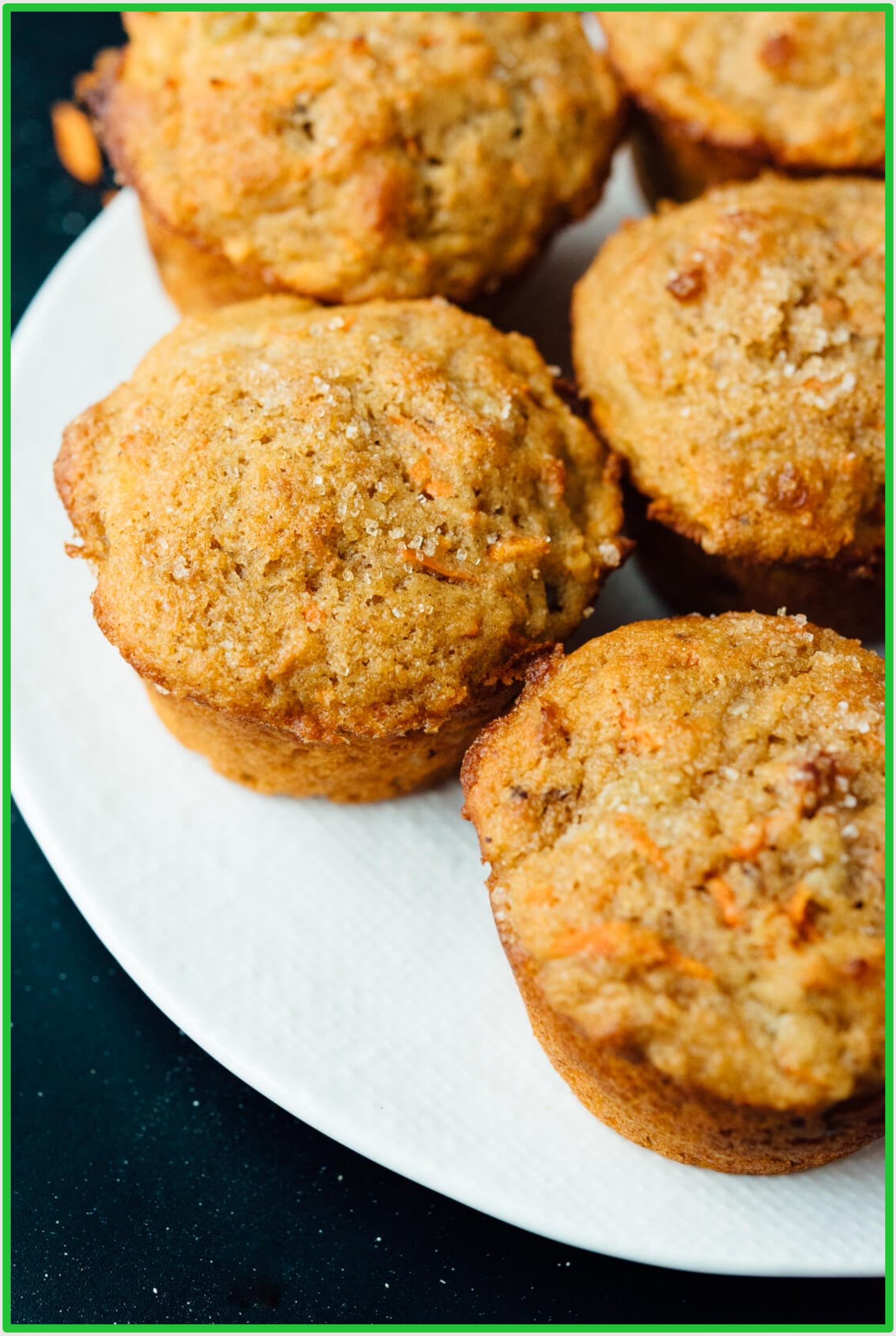

CARROT CAKE MUFFINS

INGREDIENTS

- 225g whole wheat flour
- 1 ½ teaspoons baking powder
- 1 teaspoon ground cinnamon
- ½ teaspoon baking soda
- ½ teaspoon salt
- ½ teaspoon ground ginger
- ¼ teaspoon ground nutmeg
- 2 cups peeled and grated carrots* (about 3 large or up to 6 small)
- 50g roughly chopped walnuts
- 75g raisins
- 80ml extra-virgin olive oil
- 1 cup granulated artificial sweetener
- 2 eggs
- 250g fat-free Greek yogurt
- 1 teaspoon vanilla extract

MAKES 12 SERVINGS
6g of Protein/serving

INSTRUCTIONS

1. Preheat oven to 220 degrees Celsius. Grease all 12 cups on your muffin tin with non-stick cooking.
2. In a large mixing bowl, combine the flour, baking powder, cinnamon, baking soda, salt, ginger and nutmeg. Blend well with a whisk. Add the grated carrots, raisins and chopped walnuts to the other ingredients and stir.
3. In a medium mixing bowl, combine the oil and sweetener and beat together with a whisk. Add the eggs and beat well, then add the yogurt and vanilla and mix well.
4. Pour the wet ingredients into the dry and mix with a big spoon, just until combined (a few lumps are ok). Divide the batter evenly between the 12 muffin cups. Bake muffins for 13 minutes, or until the muffins are golden on top and a toothpick inserted into a muffin comes out clean.

FRESH FRUIT & PROTEIN YOGHURT

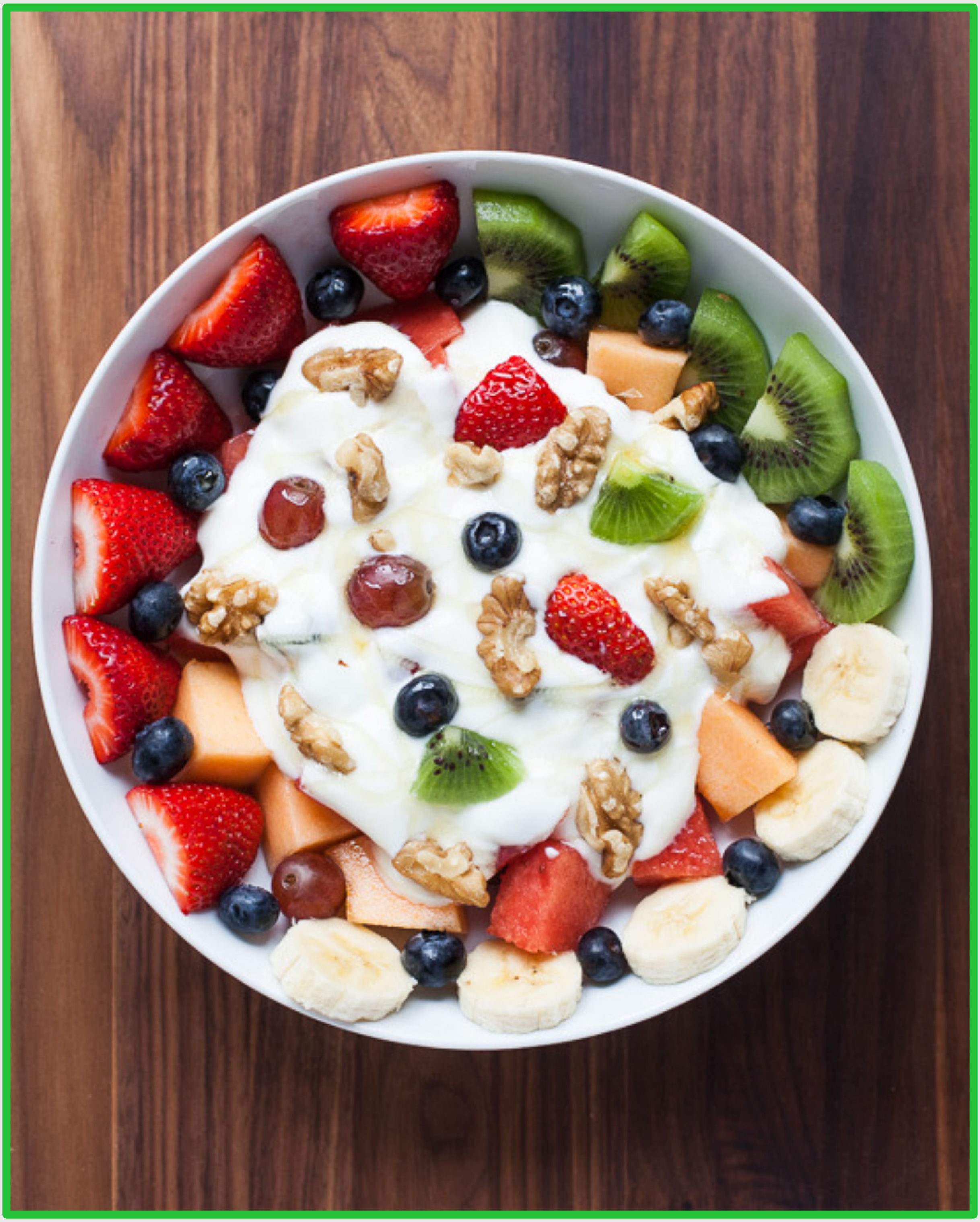

FRESH FRUIT & PROTEIN YOGHURT

INGREDIENTS

- 250g fat-free quark, skyr or Greek yoghurt
- 30g almonds or walnuts (or nuts of choice
- Artificial sweetener (to taste)
- 1-2 cups of mixed chopped fruit (apples, bananas, oranges, kiwis, peaches, pineapple, mango, grapes, pears, raspberries, strawberries, blueberries etc.

MAKES 1 SERVING
31g of Protein/serving

INSTRUCTIONS

1. Sweeten the quark or yoghurt with artificial sweetener to taste
2. Top with the chopped fruit and nuts and serve

*You can also add half a teaspoon of vanilla essence to the yoghurt for extra flavour

THE ULTIMATE LAZY MEAL

Sometimes, you want something very quick, easy and tasty to make and you might only have 5-10 minutes to make it. In that case, this "recipe" will become your best friend.

INGREDIENTS

Take one item (or a selection) from each box.

VEGETABLES
Frozen/Pre-chopped etc.

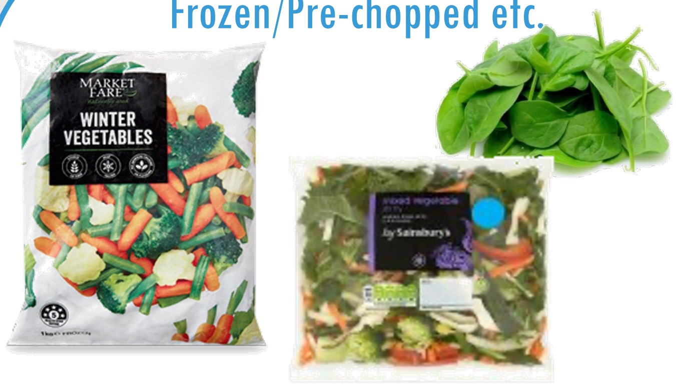

PROTEIN
Pre-Cooked/Frozen/Pre-chopped etc.

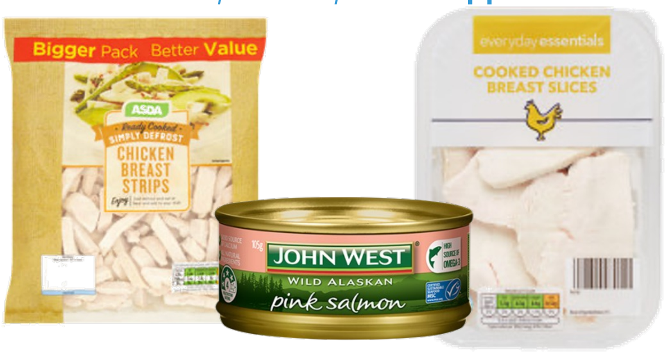

SOUP/SAUCE
1 portion (half a container)

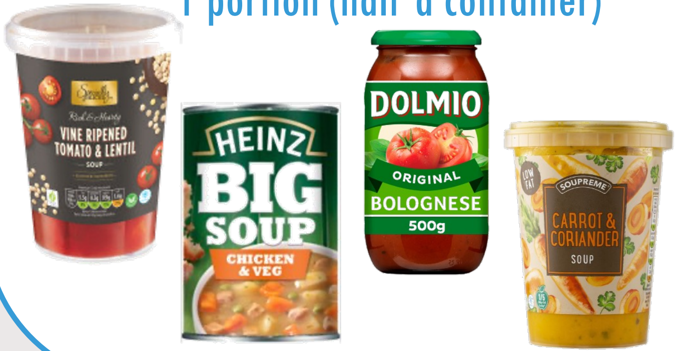

OPTIONAL

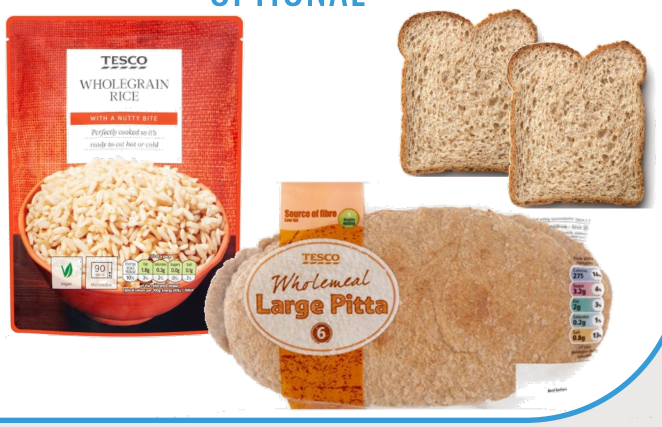

INSTRUCTIONS

1. Mix the chopped vegetables, protein and soup/sauce in a sauce pan or microwave bowl.
2. Heat (stove top or microwave) until cooked (5-10 minutes)
3. That's it. Serve with wholegrain bread or brown rice if hungry.

THE ULTIMATE LAZY MEAL

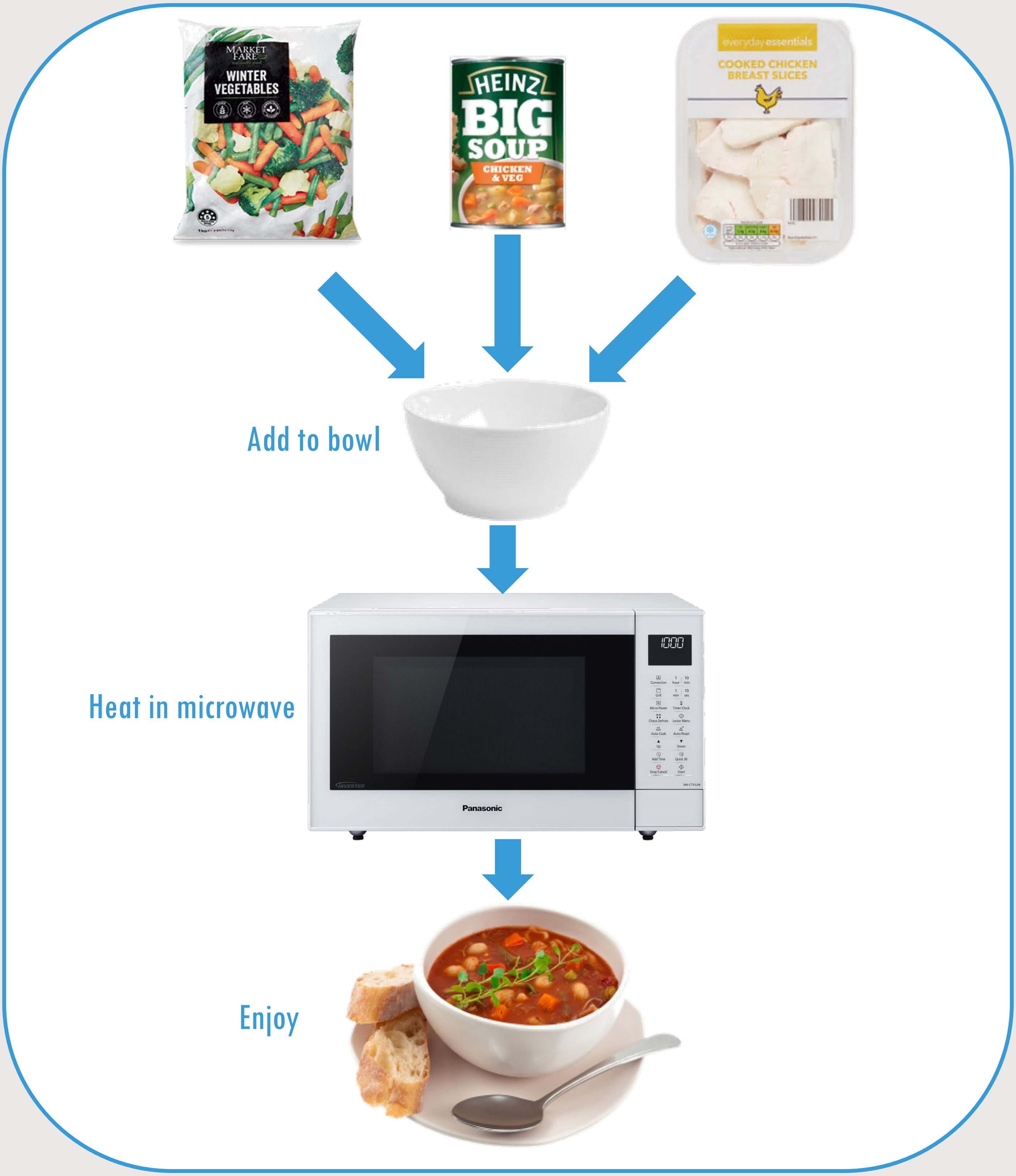

SNEAK MORE VEGGIES INTO YOUR MEALS

As you can see, vegetables are a really important part of all the recipes in this guide. The tips below will help you to include more vegetables into your other meals, easily and without affecting the taste, so you can get all the health benefits of lots of vegetables, every day.

Breakfast

Frittata it.

Eggs are a great, high-protein way to start the day. Mix eggs with veggies for a healthy and hearty breakfast

Add minced broccoli or cauliflower to scrambled eggs.

This veggie addition doesn't change the texture of eggs and fits in an entire serving of veggies (at least). Steam and purée or finely grate the veg to mix with scrambled eggs.

Bake with them.

Breakfast sweets can be packed with veggies too. Try making some travel-friendly bran muffins packed with zucchini and carrots in addition to the classic raisins, walnuts, and cinnamon.

Veg out on savory oatmeal.

Classic oatmeal might be topped with brown sugar and fruit, but oats can be savory too! Cook plain oats with water and add your choice of steamed or fried veggies. Top with an egg for extra protein and season with salt, pepper, or a sprinkle of Parmesan cheese.

Try pumpkin or butternut squash pancakes or waffles.

When the frying pan is heating, throw some pumpkin or squash purée into pancake or waffle mix to fit in an extra serving of veggies (and get a fun orange tinge, too).

Pasta & Grains

Make pasta dishes go green.

When spaghetti and meatballs is on the menu, add a load of extra veggies (like spinach, peppers and mushrooms) to the dish instead of opting for a boring side salad.

Experiment with veggie noodles.

Veggie noodles are quite easy to find in big supermarkets and allow you to skip the pasta altogether. (Or use roast spaghetti squash!) Add extra veggies to the sauce for an extra dose of nutrients.

Remember herbs are leafy greens too!

Add fresh herbs to any rice, pasta, or grain dish. Or whip up a quick homemade herb pesto to add to scrambled eggs or use as a sandwich spread.

Get fancy with shepherds pie.

It's a childhood favourite, but grown ups crave it too—don't lie! When you want some, add a load of fresh veggies for a dose of extra nutrients. Spinach, tomatoes, peas, and broccoli make great additions.

Sneak them in casseroles.

Anytime that casserole dish comes out of the cupboard, get the grater out too. Finely shredded carrot or summer squash can be added to virtually any casserole without changing taste or texture!

Smoothies

Add greens to breakfast smoothies.
A handful of spinach or kale blends well with any fruit smoothie. Try by blending 250g of fat-free quark or Greek yoghurt, 1 frozen banana, 2 handfuls spinach (and 1 tablespoon peanut butter if you like).

Slurp a carrot smoothie.
Grated carrots are easy-peasy to fit into any fruit smoothie. Bonus: Because we're using all parts of the veggie, none of the fibre is lost like in juices.

Sandwiches

Sub greens for wraps.
Lettuce makes a surprisingly good stand-in for bread and tortilla wraps.

Add veggies to grilled cheese.
Melted cheese between two slices of bread doesn't have much green value. Every time the cheesy craving strikes, throw in a few layers of veggies. Spinach, corn , tomato, and red onion make great additions.

Bulk up burgers.
Everyone loves a burger so try making them healthier by adding lots of greens like spinach and lettuce, and other veggies like tomatoes, pickles, shredded carrot, onions etc…

Pizzas

Add colour

Yes, a cheesy pizza is hard to pass up. But pizzas are a great vehicle for a big pile of veggies. Practically anything works, from greens and tomato to roasted squash or root vegetables.

Prepare a pizza salad.

If pizza is for dinner, throw a salad on top for a fun meal to eat, and an easy two-in-one dinner. A favourite? Rocket and spinach salad with tomatoes and Parmesan on top of a mixed veggie pizza.

Soups & Stews

Add veggie purée to chicken soup.

Making classic chicken soup? Add a can of puréed tomatoes, squash, or spinach. It will make for a thicker soup and also sneak in some extra veggies.

Improve on ready-made soup

Add your own vegetables; frozen, sliced, pureed to ready made soup to add even more fibre, vitamins and minerals.

Spice up chili.

Add carrot, sweet potato, or butternut squash purée, peppers and broccoli to any chili or stew recipe.
