# Supplementary material for: Acceptability of a high-protein Mediterranean-style diet and resistance exercise protocol for cardiac rehabilitation patients: Involving service users in intervention design using a mixed-methods participatory approach
Source: Front Nutr. 2023 Feb 14;10:1043391. doi: 10.3389/fnut.2023.1043391 (PMC9970995; doi:10.3389/fnut.2023.1043391)
Supplement: Supplementary Figure 1 — Study research plan. [file Data_Sheet_1.zip › Supplementary file 2 Exercise Guide.pdf]

YOUR PROGRAMME

For the next 12 weeks, you will complete a:

- full-body resistance (strength) training session
- 3 times a week

WHAT IS FULL BODY TRAINING?

Full-body training simply means that you will train a variety of muscle groups (chest, back, arms and legs) in one session.

HOW MANY EXERCISES WILL I DO?

Each session will consist of 6 different exercise including upper body pushes and pulls and lower body movements.

You will repeat the same set of exercises every other session the 12 weeks of your programme. This way you will know what to expect at every session.

HOW LONG WILL I REST BETWEEN EXERCISES

At least 1 minute but 2-3 minutes is fine. You should feel ready for your next set.

WHAT ARE SETS AND REPS?

You will hear and read the terms “sets and reps” regularly during this programme. These are terms used to describe the number of times you perform an exercise.

- A rep is the number of times you perform a specific exercise (lifting a weight once is “1 rep”)
- A set is a cycle of reps that you complete.

For example, suppose you complete 15 reps of a chest press. You would say you've completed "one set of 15 reps." A set can be any number of reps, so if you complete 10 reps of a bench press, you would say you've completed "one set of 10 reps," and if you complete just five reps, then that would be "one set of five reps."

HOW MANY SETS AND REPS SHOULD I DO?

For each exercise you will perform:

- 1-4 sets of
- 8-12 reps

You will intially start doing only 1-2 sets of each exercise. Your first session will be to establish what your starting weights should be for each exxercise. The number of sets will increase weekly until you can do 4 sets and will remain at 4 sets for the remaining sessions.

HOW MUCH WEIGHT SHOULD I USE?

At your first session, the trainers at your gym will teach you how to do the exercises and they will also tell you how much weight you should start with, depending on how much weight you can lift relatively comfortably.

Throughout the programme you should lift until **"near failure"** meaning until you can't lift the weight again with correct/good form. Your trainer will explain this fully but this is very, very important. To get the most out of this training you really need to train hard.

WILL MY WEIGHTS, SETS AND REPS STAY THE SAME?

No! The objective of this programme is to increase the weight gradually over time as your muscles get used to the weights you lift, with the objective of gaining strength over time.

For example, when you start, you may use 20kg on chest press.

- Your first week, you will do 1-2 sets of 8-12 reps
- Once you can do both sets for 12 reps, you will add another set
- When you can do all 3 sets for 12 reps, you will add another set
- When you can do all 4 sets for 12 reps, you will increase the weight on the machine to the next level (for example 25kg)
- The reps you can do with each set will probably drop but you won't increase the weight again until you can do all 4 sets for 12 reps
